# A phylogenomic approach to reconstruct interrelationships of main clupeocephalan lineages with a critical discussion of morphological apomorphies

**DOI:** 10.1186/s12862-018-1267-1

**Published:** 2018-10-23

**Authors:** Nicolas Straube, Chenhong Li, Matthias Mertzen, Hao Yuan, Timo Moritz

**Affiliations:** 1 Institut für Zoologie & Evolutionsbiologie, Friedrich-Schiller-Universität Jena, Erbertstraße 1, 07743 Jena, Germany; 2 Zoologische Staatssammlung München, Staatliche Naturwissenschaftliche Sammlungen Bayerns, Münchhausenstraße 21, 81247 Munich, Germany; 3 Key Laboratory of Exploration and Utilization of Aquatic, Genetic Resources, Shanghai Ocean University, Ministry of Education, Shanghai, 201306 China; 4 Deutsches Meeresmuseum, Katharinenberg 14-20, 18439 Stralsund, Germany

**Keywords:** Otomorph characters, Euteleostei, Alepocephaliformes, Synapomorphies, Target gene capture, Next generation sequencing, Evolmarkers

## Abstract

**Background:**

Previous molecular studies on the phylogeny and classification of clupeocephalan fishes revealed numerous new taxonomic entities. For re-analysing these taxa, we perform target gene capturing and subsequent next generation sequencing of putative ortholog exons of major clupeocephalan lineages. Sequence information for the RNA bait design was derived from publicly available genomes of bony fishes. Newly acquired sequence data comprising > 800 exon sequences was subsequently used for phylogenetic reconstructions.

**Results:**

Our results support monophyletic Otomorpha comprising Alepocephaliformes. Within Ostariophysi, Gonorynchiformes are sister to a clade comprising Cypriniformes, Characiformes, Siluriformes and Gymnotiformes, where the interrelationships of Characiformes, Siluriformes and Gymnotiformes remain enigmatic. Euteleosts comprise four major clades: Lepidogalaxiiformes, Protacanthopterygii, Stomiatii, and Galaxiiformes plus Neoteleostei. The monotypic Lepidogalaxiiformes form the sister-group to all remaining euteleosts. Protacanthopterygii, comprising Argentini-, Esoci- and Salmoniformes, is sister to Stomiatii (Osmeriformes and Stomiatiformes) and Galaxiiformes plus Neoteleostei.

**Conclusions:**

Several proposed monophyla defined by morphological apomorphies within the Clupeocephalan phylogeny are confirmed by the phylogenetic estimates presented herein. However, other morphologically described groups cannot be reconciled with molecular phylogenies. Thus, numerous morphological apomoprhies of supposed monophyla are called into question. The interpretation of suggested morphological synapomorphies of otomorph fishes is strongly affected by the inclusion of deep-sea inhabiting, and to that effect morphologically adapted Alepocephaliformes. Our revision of these potential synapomorphies, in the context that Alepocephaliformes are otomorph fishes, reveals that only a single character of the total nine characters proposed as synapomorphic for the group is clearly valid for all otomorphs. Three further characters remain possible apomorphies since their status remains unclear in the deep-sea adapted Alepocephaliformes showing developmental lag and lacking a swim bladder. Further, our analysis places Galaxiiformes as sister group to neoteleosts, which contradicts some previous molecular phylogenetic studies. This needs further investigation from a morphological perspective, as suggested synapomophies for several euteleostean lineages are challenged or still lacking. For the verification of results presented herein, a denser phylogenomic-level taxon sampling should be applied.

**Electronic supplementary material:**

The online version of this article (10.1186/s12862-018-1267-1) contains supplementary material, which is available to authorized users.

## Background

With approximately 32,000 species, the teleost fishes comprise about half of the vertebrate species, with representatives in almost any aquatic environment from montane habitats to the deep-sea. With the progress in gene sequencing technologies, several phylogenetic hypotheses have been published for Teleostei in recent years advancing from single gene alignments to mitochondrial genomes and multi-locus approaches increasing in the taxonomic diversity analyzed. However, major discrepancies are evident between morphology and molecular phylogenetics [1–6] and between different DNA sequence based datasets. Further, several deep phylogenetic nodes remain enigmatic [2]. In the course of this paper, we are referring to the classification suggested in [2], if not indicated otherwise.

**Fig. 1:**
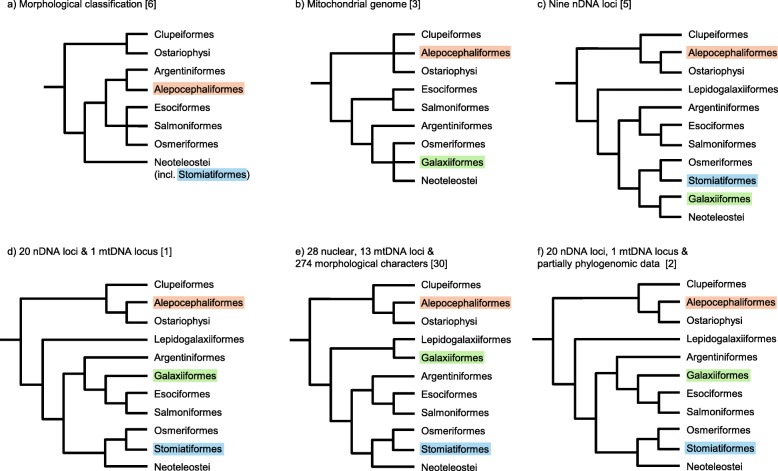
Summary of previous phylogenetic estimates and classifications of Clupeocephalan fishes. Colours indicate taxa with variable phylogenetic positions

Phylogenetic analyses suggest that Teleostei comprises three main lineages: Osteoglossomorpha, Elopomorpha and Clupeocephala with the latter being the largest by far. The monophyly of the supercohort Clupeocephala is evidenced by both morphological and molecular data [1, 2, 5–7]. Wiley and Johnson [6] suggested Clupeocephala to contain two major lineages, the Otomorpha and Euteleostei (Fig. 1a). Otomorpha are suggested to comprise the Clupei (herrings and allies, also referred to as Clupeomorpha) and Ostariophysi. The diverse subcohort Ostariophysi includes the most species rich and predominantly fresh water inhabiting lineages, the Cypriniformes (carps) as well as the Siluriformes (catfishes). Further, the clade comprises Gonorynchiformes (milkfishes), Characiformes (characins and allies) and Gymnotiformes (neotropical knifefishes) (Fig. 1). With more than 10,000 described species, the Ostariophysi pose a noteworthy part of today’s Clupeocephalan overall diversity. The situation gets complicated by results from molecular phylogenetic studies providing strong evidence that Alepocephaliformes are Otomorpha [1, 2, 5, 8–10] (Fig. 1b,c). Contrasting all other otomorph fishes, Alepocephaliformes represent a group of deep-sea fishes, which show extensive morphological adaptations to their habitat making a morphological comparison to other taxa within Otomorpha difficult. The first detailed morphological investigation on the systematic position of alepocephaliformes [11] placed them as sistergroup to the Argentinoidei (marine smelts and allies), which was adopted in the classification of [6], placing the Alepocephaloidei in the order Argentiniformes among euteleost fishes. Therefore, molecular phylogenetic analyses challenge proposed morphological synapomorphies on at least five phylogenetic levels rendering higher-level taxa Euteleostei, Otomorpha, Protacanthopterygii, Argentinoidei and Alepocephalodei either poly- or paraphyletic.

A sister-group relation of clupeomorphs and ostariophyseans was first proposed by Lecointre and Nelson [12]. The respective taxonomic unit, the Otomorpha [6], also named Otocephala [13] or Ostarioclupeomorpha [14], was soon established, but morphological evidence supporting the monophyly of the group remained scarce [6, 12, 14, 15]. The following nine characters have been discussed as possible apomorphies for otomorphs: (1) ossification of autopalatine early in ontogeny- within the palatoquadrate cartilage three bones autogenously ossify. The ancestral teleost condition is an autopalatine, which forms clearly later than metapterygoid and quadrate [16]. Arratia and Schultze [16] found that the autopalatine in *Denticeps*, *Dorosoma* and *Chanos* ossifies at the same ontogenetic stage. In salmonids, it ossifies slightly later compared to the otomorph palatoquadrate ossification sequence and the authors suggested an early ontogeny of an autopalatine as synapomorphy for Clupeocephalans. In more primitive taxa such as Osteoglossomorpha or Elopomorpha, the ossification of the autopalatine takes place significantly later. Subsequent studies proposed that this character was not apomorphic for clupeocephalans but otomorphs [12–15]. Later, Arratia [7, 17] revised this statement and re-defined the character as apomorphic for the Clupeocephalans again. (2) Fusion of medial extrascapulars with parietals- the absence of a separate median sensory canal bone in the supratemporal commisure, resulting in a canal in the parietal and supraoccipital bones, was first reported from clupeomorphs and regarded as apomorphic for this group [18]. Such a condition is usually interpreted as a fusion of the medial extrascapular to the parietal [12]. A respective fusion without inclusion of the supraoccipital was also reported from some species of Gonorynchiformes [19], Cypriniformes, and Characiformes [20, 21]. This led to the conclusion that the character is of apomorphic state in Otomorpha [6, 12, 15].

(3) Ossified epicentrals- ossified epicentrals are documented already in the Elopomorpha [22], which excludes this character as an apomorphy for Otomorpha. (4) Connection of swim bladder and ear - the otophysic connection, i.e. the connection between swim bladder and inner ear, fundamentally differs in Clupeomorpha and Otophysi [23–25]. The evolutionary sequence between these two states is unknown. Furthermore, gonorynchiform species do not show an otophysic connection [23, 25], but only show adaptations, which can be interpreted as ancestral conditions of an otophysic connection of the Ostariophysi-type: the division of the swim bladder into an anterior and posterior chambers and an enlarged first rib. This leads to the conclusion that this character cannot be regarded as an apomorphy for otomorphs. Alepocephalids lack a swim bladder and and thus cannot contribute to evaluate this character. (5) Anterior chamber of swim bladder partly or completely covered by silvery peritoneal tunic- a completely or partially silvery peritoneal cover of the anterior chamber of the swim bladder [23] was discussed as possibly apomorphic for Ostariophysi [24]. Clupeomorph swim bladders do not show an anterior-posterior division as in Ostatariophysi, however, within their single chambered swim bladder, the anterior part is also covered with a silvery peritoneal tunic. Therefore, this character is regarded as apomorphy for otomorphs [26]. (6) Heamal spines anterior to second preural centrum fused to centra early in ontogeny- the character “hemal spine of preural-centrum 3 (PU3) and anterior vertebrae fused with their respective centra” [26] usually is supplemented with the information that the character is already developed “from a young juvenile stage on” [6, 14, 24]. Fink and Fink [24] found that also clupeomorphs have all hemal spines fused to their centra and interpreted this condition as hint to a “relationship between Clupeomorpha and Ostariophysi”. Later, however, the Fink and Fink [24] listed the same character as an ostariophysean apomorphy disregarding the clupeomorph condition [24]. The interpretation of this character in the light of recent phylogenetic hypothesis is difficult as the deep-sea dwelling alepocephalids show reductions and developmental lag in ossifications. (7) Presence of a pleurostyl- a pleurostyl is found in clupeoids and Ostariophysi [27, 28]. Most clupeiforms and Ostariophysi have a pleurostyl. The sister-taxon of all clupeiforms, *Denticeps,* as well as fossil stem-group representatives of Ostariophysi, however, do not show this character. In herring-like fishes, it is a fusion of the first uroneural to the (first) preural centrum [27, 28]. Respective fusions in the caudal skeleton of Ostariophysi are more comprehensive and include additionally both ural centra and the hypural 2 [24]. (8) Lack of cartilaginous connection between the bases of hypurals 1 and 2- the lack of a cartilaginous connection between bases of the hypurals 1 and 2 at any ontogenetic stage was proposed as apomorphy for otomorphs [6, 14, 15]. (9) Fusion of hypural 2 with compound centrum. A fusion of hypural 2 with the first ural centrum is present in Otophysi and Clupeomorpha [6, 24, 27, 28]. Such a fusion is, on the other hand, absent from all gonorynchiforms except *Gonorynchus* in which, however, caudal element fusions are extensive [25].

In summary, morphological evidence supporting the taxon otomorpha is scarce. Further, several of the proposed apomorphic characters are doubtful and have already been critically discussed [6, 7, 12, 13].

With advances in sequencing technology, phylogenetic analyses shifted from analysing morphological matrices to sequence alignments with growing number of genes and taxa included in the analyses. Based on molecular phylogenetic results, Betancur-R. et al. [2] define the Euteleostei (referred to as Eutleosteomorpha in [2]) to comprise several newly defined clades in their new classification of bony fishes, for example the Lepidogalaxiiformes, a taxonomic unit comprising a single extant species only, the West-Australian salamander fish *Lepidogalaxias salamandroides*. Molecular phylogenetic analyses suggest this species to form the sister taxon to all other euteleost lineages [29]. Besides its unexpected phylogenetic position suggested by molecular data, it also displays numerous noteworthy morphological characters indicating strong specialization and, contrasting molecular phylogenetics, a close phylogenetic relationship with Galaxiiformes [30]. Following Betancur-R. et al. [1, 2], the sister clade of Lepidogalaxiiformes is unnamed including three major clades: the Protacanthopterygii as sister to a clade comprising Stomiatii and Neoteleostei. The Protacanthopterygii sensu Betancur-R. et al. [1, 2] comprise Argentiniformes, Galaxiiformes, Salmoniformes (salmons) and Esociformes (pikes and mudminnows) (Fig 1c). It should be noted that, contrasting [1, 2], Near et al. [5] found the Galaxiiformes to form a sister group relationship with neoteleosts, i.e. the order was not clustering along with the Protacanthopterygii sensu Betancur-R. et al. [1, 2] (Fig. 1d, f). From a morphological perspective, another noteworthy result from DNA sequence data is the sister group relationship of Osmeriformes (smelts) with Stomiatiformes (dragonfish) forming the Stomiatii (Fig. 1c, d e, f). Morphological studies considered dragonfishes hitherto as neoteleost fishes sharing proposed neoteleost synapomorphies, especially in branchial arch musculature and tooth attachment type [6]. Wiley and Johnson [6] commented that homology of these characters has not been evaluated from an ontogenetic perspective in Stomiatiformes and such information is still lacking. Hence, the classification in Betancur-R. et al. [1] challenged the morphological monophyly of neoteleosts and calls the suggested synapomorphic characters into question. Results presented in Betancur-R. et al. [1, 2] reproduce neoteleost fishes as sister to the Stomiatii/ Protancanthopterygii clade. In their phylogenetic tree reconstruction, however, this split lacks high node support, confirming the split between Stomiatii and Neoteleosts. A similar situation is apparent in the sister group relationship of Protacanthopterygii and the Stomiatii/ neoteleost clade. With updating the classification of bony fishes [2], Protacanthopterygii are sister to a clade now comprising Stomiatii and Neoteleostei. Among neoteleosts, Ateleopodiformes (jellynose fishes) are sister to all further higher-level taxa referred to as Eurypterygia (Cyclosquamata, Ctenosquamata, Acantomoprhata, Euacantomorphacea, and Percomorphata).

In a recent and extensive study, Mirande [31] combines both morphological and molecular data to re-infer the major phylogenetic relationships within Acanthopterygii. The parsimony-based phylogenetic estimates [31] are only partially in accordance with studies analyzing solely molecular data, demonstrating the contrarious phylogenetic signals by morphological and molecular data. The final hypothesis of Mirande [31] recovers the Clupeocephala with high Bremer support, while the Otomorpha, including the Alepocephaliformes as sister to the remaining otomorph taxa, appear only weakly supported. The well-supported euteleosts show two major clades, one comprising Lepidogalaxiiformes and Galaxiiformes as sister groups to all remaining euteleost lineages. The monophyly of the Lepidogalaxiiform/ Galaxiiform clade is only weakly supported. A sister group relationship of Protacanthopterygii (sensu Betancur-R. et al. [1, 2]) and Stomiatii is not well supported, while the monophyly of neoteleosts appears underpinned based on Bremer support (Fig. 1e).

In summary, several taxonomic entities proposed in previous phylogenetic studies analyzing molecular data are not supported by presently available morphological data. In times of next generation sequencing, researchers started to conduct phylogenomic level approaches to resolve difficult phylogenetic questions such as otophysan interrelationships [32, 33].

Here, we apply a targeted gene capture approach using a set of curated RNA baits to attain a phylogenomic-level dataset of potential ortholog loci to test, if we can sequence a sufficient number of genes from non-model organisms to resolve deep phylogenetic nodes on ordinal level within the evolutionary oldest Clupeocephalan lineages. In this study, we test if (1) we can resolve the otomorph phylogeny and (2) discuss previously suggested morphological synapomorphies of Otomorpha in the light of our phylogenomic reconstruction. (3) New insights in to the phylogeny of deep phylogenetic nodes of Euteleostei are discussed with regard to previous phylogenetic reconstructions and morphological characters.

## Results

**Fig. 2:**
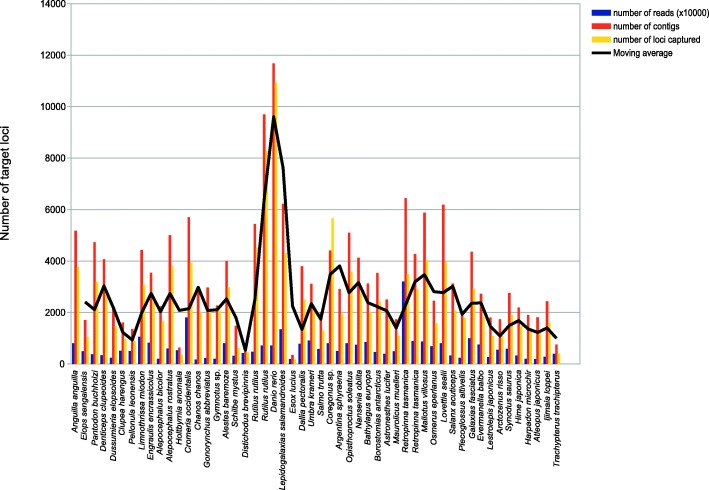
Summary of target capture success. X-axis: target species; y-axis: number of target loci captured per specimen

Sequencing of 52 taxa resulted in an average number of 6.3 million paired-end reads per specimen. After adaptor and quality trimming of reads, per taxon reads were blasted [34] against bait sequences to create gene bins. Reads mapped to target loci were *de-novo* assembled resulting on average in 3400 contigs per specimen. After reciprocally blasting specimen contigs against the reference genome, which was used for bait design, 368 loci were identified as potential paralogs and excluded from further analysis steps. 13,681 loci were available for the cross-contamination check. The highest percentage of potential cross-contamination between pair of taxa among these loci was only 1.44%, which meant there was no cross-contamination among our samples. (Additional file 1: Table S2). The latter were excluded from further analyses. The search for phylogenetically informative loci applying the Matrix Reduction Software MARE [35, 36] identified 838 most informative loci. Additional checks for orthology [37, 38] identified four further loci flagged as potential paralogs (Additional file 1: Table S3) and were exlcuded from subsequent analysis. Another five loci were excluded, which contained less than four taxa. These 829 most informative loci were phylogenetically analysed totaling 202,922 possible sites per specimen in the concatenated nucleotide alignment. RAxML found 117,046 distinct alignments patterns and a proportion of gaps and undetermined characters of 60.3%. The alignments are available for download at the Dryad data repository [39]. See Fig. 2 and Additional file 1: Table S1 for a summary of target capture success.

### Phylogenetic analyses

The results from Partitionfinder and PartitionfinderProtein [40–42] and best-fitting partitioning schemes for the maximum likelihood analyses of the concatenated datasets are available for download at the Dryad data repository [39]. ESS values derived from the Phylobayes [43] analyses are further listed in Additional file 1. Overall, results were indicating high quality runs, as the comparisons of bipartition frequencies is around 0.1 for both runs (Additional file 1).

**Fig. 3:**
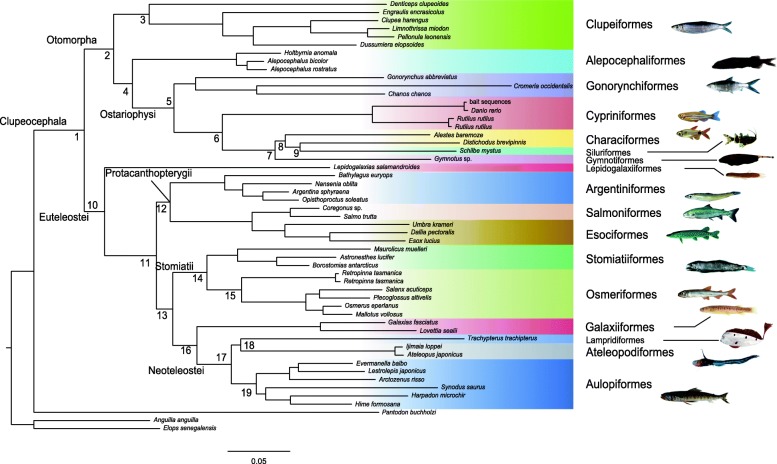
Phylogenetic reconstruction based on concatenated DNA sequence alignments of 52 taxa (see Additional file 1: Table S1) using RAxML [42] and best partitioning scheme resulting from a Partitionfinder analysis [40, 41]. Numbers at nodes refer to Table 1. Tree re-rooted with Elopomorpha (*Elops senegalensis* and *Anguilla anguilla*)

Different phylogenetic estimates in this study are widely congruent with few exceptions, which will be subject of the discussion. Please refer to Fig. 3 and Table 1 for tracking the results listed below. After rooting all resulting trees with the two elopomorph taxa *Elops senegalensis* and *Anguilla anguilla*, *Pantodon buchholzi*, representing the Osteoglossomorpha, forms the sister group of the monophyletic and well-supported Clupeocephala. Clupeocephala are split in two major clades, Otomorpha, comprising Clupeiformes, Alepocephaliformes and Ostariophysi (*Rutilus* and *Danio* (Cypriniformes), *Gonorynchus, Cromeria* and *Chanos* (Gonorynchiformes), *Gymnotus* (Gymnotiformes), *Alestes* and *Distichodus* (Characiformes) and *Schilbe* (Siluriformes), and euteleosts including *Lepidogalaxias* (Lepidogalaxiiformes), *Galaxias* and *Lovettia* (Galaxiiformes), *Esox*, *Dallia* and *Umbra* (Esociformes), *Bathylagus, Nansenia, Argentina* and *Opisthoproctus* (Argentiniformes), *Salmo* and *Coregonus* (Salmoniformes), Stomiatii (*Osmerus*, *Mallotus*, *Salanx* and *Plecoglossus* representing Osmeriformes) and *Borostomias, Astronesthes* and *Maurolicus* representing the Stomiatiformes). Monophyletic Neoteleostei are part of the euteleosts and represented by several species of Aulopiformes, Ateleopodiformes and *Trachypterus* (Lampridiformes).

**Table 1:** Overview of information on node numbers provided in Fig. 3

Node number	node description	bootstrap support maximum likelihood analysis concatenated AA alignments, partitioned	bootstrap support maximum likelihood analysis concatenated amino acid alignments, non partitioned	bootstrap support maximum likelihood analysis concatenated DNA alignments, partitioned	bootstrap support maximum likelihood analysis concatenated DNA alignments, non partitioned	phylobayes analysis based on concatenated AA alignments	phylobayes analysis based on concatenated DNA alignments	ASTRAL species tree based on AA alignments	ASTRAL species tree based on DNA alignments	IC/ICA values plotted on ML tree based on the non-partitioned DNA dataset
		Additional file 1: Figure S1	Additional file 1: Figure S2	Additional file 1: Figure S3	Additional file 1: Figure S4	Additional file 1 Figure S5	Additional file 1: Figure S6	Additional file 1: Figure S7	Additional file 1: Figure S8	n.a.
1	Split Otomorpha/ Euteleostei	100	100	100	100	1	1	1	1	0.404/ 0.404
2	Split Clupeiformes/ (Ostariophysi; Alepocephaliformes)	n.a.	n.a.	100	100	n.a.	1	n.a.	1	0.621/0.021
3	radiation Clupeiformes	100	100	100	100	1	1	1	1	0.545/ 0.545
4	Split Alepocephaliformes/ Ostariophysi	80 (Clupeiformes)	80 (Clupeiformes)	100	100	1 (Clupeiformes)	1	0,94 (Clupeiformes)	0,88	0.599/ 0.599
5	Split Gonorynchiformes/ Otophysa	100	100	100	100	1	1	1	n.a.	0.705/ 0.705
6	Split Cypriniformes/ (*Gymnotus*, *Schilbe*, Characiformes)	100	100	100	100	1	1	1	1	0.710/0.710
7	Split (Gymnotus/ (Characiformes/ *Schilbe*)	100	100	n.a.	n.a.	n.a.	n.a.	n.a.	n.a.	0.316/ 0.316
8	Split (Schilbe (*Alestes*/ * Distichodus))*	65	63	n.a.	57	n.a.	n.a.	n.a.	n.a.	0.025/ 0.025
9	Split (*Distichodus*/ * Schilbe)*	52	43	63	55	n.a.	0,77	0,48.	n.a.	−0.119/ -0.119
10	Split *Lepidogalaxias*/ remaining Euteleostei	100	100	100	100	1	1	1	1	0.603/ 0.603
11	Split “Protacanthopterygii”/ (Stomiatii, Galaxiiformes, Neoteleostei)	100	100	100	100	n.a.	1	1	1	0.661/ 0.661
12	Split Argentiniformes/ (Salmoniformes, Esociformes)	88	95	87	77	n.a.	0,98	0,55	0,97	0.505/ 0.505
13	Split Stomiatii/ (Galaxiiformes, Neoteleostei)	100	100	100	100	1	1	1	1	0.480/ 0.480
14	Split Stomiatiformes/ Osmeriformes	100	100	100	100	1	1	1	1	0.679/ 0.679
15	Split Retropinnidae/ remaining Osmeriformes	100	100	100	100	1	1	1	1	0.864/ 0.864
16	Split Galaxiiformes/ Neoteleostei	100	100	100	100	1	1	0,76	1	0.254/ 0.254
17	Split *Trachypterus*/ (Ateleopodiformes/ Aulopiformes)	100	100	100	100	n.a.	n.a.	n.a.	1	0.316/ 0.316
18	Split Ateleopodiformes/ Lampridiformes	n.a.	n.a.	92	87	n.a.	n.a.	n.a.	0,87	0.797/ 0.797
19	Split (*Synodus*/ *Harpadon, Hime*)/ (*Evermanella, Arctozenus, Lestrolepis*)	100	100	100	100	1	1	n.a.	n.a.	0.211/ 0.211

Within Otomorpha, we recovered three major clades, Clupeiformes, Alepocephaliformes, and Ostariophysi. Clupeiformes are sister to a clade comprising Alepocephaliformes and Ostariophysi (Fig. 3). Alepocephaliformes as sister to Ostariophysi is well-supported by all analyses based on nucleotide alignments, whereas amnio acid based analyses result in Alepocephaliformes as sister to a clade comprising Clupeiformes and Ostariophysi (Table 1). Different types of phylogenetic analyses and datasets (i.e. concatenated amino acid versus concatenated DNA alignments and coalescent analyses) partially show weak node support for the phylogenetic placement of Alepocephaliformes as sister to Ostariophysi (Fig. 3, Table 1). The ASTRAL [44] species tree computed from maximum likelihood trees based on amino acid single loci alignments result in a topology where Alepocephaliformes are sister to a clade including Clupeiformes and Ostariophysi (Additional file 1: Figure S7). In several phylogenetic analyses *Gonorynchus* forms a distinct lineage as sister-group to all remaining Ostariophysi (Table 1; Additional file 1: Figures S6 & S8). In all other analyses (Table 1), monophyletic Gonorynchiformes are clearly supported as the sister group of Otophysi (Table 1; Additional file 1: Figures S1-S5, S7).

All analyses recover Cypriniformes as sister to a clade comprising Characiformes, *Gymnotus* (Gymnotiformes) and *Schilbe* (Siluriformes) with high node support (Table 1), however, the relationships within the latter clade differ between analyses. While several analyses result in *Gymnotus* forming the sister lineage to a clade comprising *Alestes*, *Distichodus* and *Schilbe* (Table 1; Additional file 1: Figures S3, S4, S8), i.e. rendering Characiformes paraphyletic, only the concatenated amino acid dataset recovers interrelationships as suggested from morphology e.g. [24] and a comprehensive recent phylogenomic study [32] (Table 1, Additional file 1: Figures S1–2).

Regarding euteleost lineages, the phylogenetic analyses recover monospecific *Lepidogalaxias* as sister-group to all remaining euteleost groups (Table 1, Fig. 3, Additional file 1: Figures S1–8). The major euteleost clade comprises protacanthopterygians sensu [1, 2] excluding Galaxiiformes, i.e. Argentiniformes sister to a clade comprising Esociformes and Salmoniformes. This clade is sister to a clade including Stomiatii sensu Betancur-R. et al. [1, 2], Galaxiiformes and Neoteleostei. All analyses performed here support monophyletic Stomiatii comprising Stomiatiformes and Osmeriformes. *Retropinna* is sister to a clade comprising further osmeriforms representing the major families. The Stomiatii are the sister clade to Galaxiiformes and Neoteleostei. The Galaxiform samples (*Galaxias* and *Lovettia*) included in this study form the sister taxon to the neoteleost lineage in all analyses with high node support (Table 1) contradicting results presented in Betancur-R. et al. [1, 2].

The following splits are variable in different types of phylogenetic analyses. Protacanthopterygii (excluding Galaxiiformes) appears paraphyletic in the analysis of the concatenated amino acid alignment (Additional file 1: Figure S2) using the Bayesian inference (Additional file 1: Figure S5). In these analyses, Esociformes are sister to a clade including Argentiniformes, Osmeriformes, Galaxiiformes, Stomiatiformes, and neoteleost fishes, while all other analyses reconstruct a phylogeny as shown in Fig. 3, however, node support values for the split are partially low (Table 1).

Within neoteleosts, our results show two possible cladograms. Either two clades, where Aulopiformes is sister to a clade comprising Ateleopodiformes and *Trachypterus* (Table 1, Additional file 1: Figures S3, S4, S8) or *Trachypterus* as sister to remaining neoteleost lineages included in this study (Table 1, Additional file 1: Figures S1, 2, unresolved in S5 and S6).

Except for few nodes, bootstrap and posterior node support values are high, i.e. above 95% or 0.95, respectively (Table 1). Computed IC and ICA values [42, 45–47] do not indicate conflicting bipartitions except for the nodes, which will be discussed below and are also marked with low node support values (Table 1). The overall relative tree certainty is 0.48 indicating low incongruence among trees. The AU test performed in CONSEL [48–51] ranks the phylogenetic estimates based on the concatenated nucleotide datasets higher than all other bifurcating trees, where the tree estimate based on the partitioned nucleotide alignment ranks highest (Additional file 1: Table S4).

## Discussion

### Molecular and morphological evidence for the Otomorpha

All phylogenetic reconstructions performed in this study readily result in well-supported Otomorpha as sister clade to Euteleostei and include Alepocephaliformes, as previously found in molecular studies [1–5, 10]. However, the phylogenetic placement of the alepocephaliforms within Otomorpha remains not completely ascertained as reflected in weak node support and low IC and ICA values indicating incongruence (Table 1). Future studies should include a denser taxon sampling covering the different inter- and intra-otomorph lineages to verify results presented herein.

Our review of hitherto proposed synapomorphic morphological characters in the light of the phylogenetic reconstruction (Fig. 3) indicates that morphological evidence supporting Otomorpha including Alepocephaliformes is presently limited. After taking into account previous reviews of morphological characters [6, 12, 13, 17, 26], nine characters have been discussed as possible apomorphies (see above) for Otomorpha. Three do not withstand thorough investigation, even without considering the inclusion of alepocephaliforms, i.e. the early ossification of the autopalatine which is apomorphic for clupeocephalans [7, 17], the ossified epicentrals, which are plesiomorphic [22], and the otoyphsic connection, which principally differs in clupeomorphs and ostariophysi [18, 23, 24] in a way that no transitional states seem likely. For the latter, the fossil record and the condition in Gonorynchiformes further contradict a possible synapomorphic state. Therefore, six characters remain as candidates to characterize the Otomorpha without including alepocephaliforms: (1) parietals fused with extrascapulars, (2) anterior part of swim bladder with silvery peritoneum, (3) fusion of haemal spines anterior of preural centrum 2 with their centra from an early stage on, as well as three characters dealing with the caudal fin skeleton: (4) the absence of a united cartilaginous basis of hypurals 1 and 2, (5) fusion of hypural 2 with the compound centrum, and (6) the presence of a pleurostyl.

**Fig. 4:**
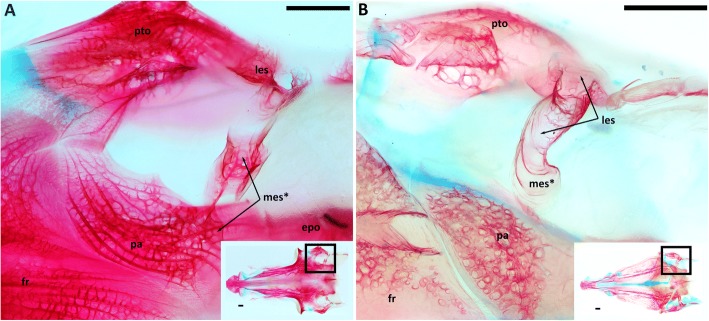
Dorsal view on the lateral neurocranium with focus on medial extrascapular (marked with an arrow) and parietal (Character 2). Cartilages are blue and bones are red. **a**: *Holtbyrnia anomala,* 144 mm SL, **b**: *Normichthys operosus*, 97 mm SL. Abbreviations: epo, epioccipital; fr, frontal; les, lateral extrascapular; mes, medial extrascapular; pa, parietal; pto, pterotic. Scale bar =1 mm

Discussing the six characters in Alepocephaliformes reveals the difficulties arising when analysing such a morphologically highly specialized taxon. Fig. 4 (A and B) shows the dorsal view on the posterior right part of the neurocranium in two alepocephalid species focusing on medial extrascapulars. The ossified canals of the occipital commisure can be interpreted as the remnants of extrascapulars. They are not fused with the parietals in any analysed alepocephalid species, exemplified in Fig. 4. This challenges the synapomorphic state of character 1. In Lecointre and Nelson [12], a fusion of the extrascapular with the parietal in two alepocephalids, i.e. *Leptoderma* and *Rouleina* is described. A more detailed sample of alepocephalid species is necessary to fully evaluate character 1 and clarify, if this poses a convergence in *Leptoderma* and *Rouleina* with clupeomorphs and ostariophysi or a reversal in other alepocephaliforms species. Alepocephaliformes lack a swim bladder, which is likely owed to their deep-sea inhabiting lifestyle. Therefore, the details of the swim bladder such as character 2 can generally not be analysed. This does not exclude the possibility, that this character is still a synapomorphy, as it may have been reduced in the course of adapting to deep-sea conditions and could well be present in the common ancestor.

**Fig. 5:**
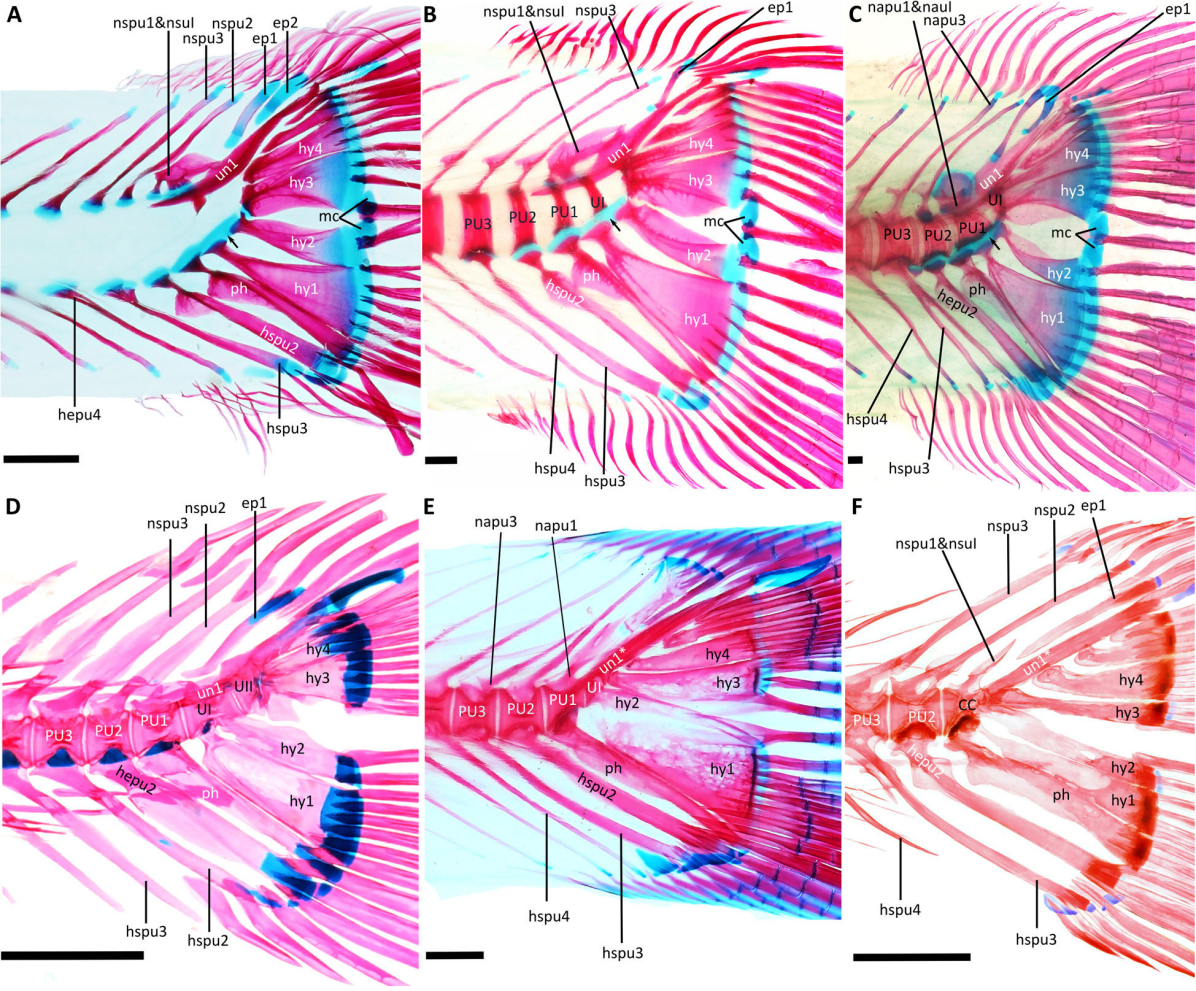
Caudal skeleton, **a** and **b**: *Holtbyrnia anomala*, 55 mm SL, 118 mm SL, **c**: *Maulisia argipalla*, 115 mm SL, **d**: *Denticeps clupeoides,* 31 mm SL (epineurals and epipleurals were removed), **e**: *Clupea harengus,* 83 mm SL, **f**: *Dawkinsia tambraparniei,* 27 mm SL (epineurals and epipleurals were removed). Cartilages are blue and bones are red. The star marks the uroneural, which is fused with the compound center and thus is a pleurostyl (Character 7). The arrow in **a-c** marks the common cartilaginous base of the hypurals 1 and 2 (Character 8). Abbreviations: CC: compound centrum = PU1 + UI + UII + napu1 + nauI + un1 + hy2; ep, epural; hepu, haemal arch of preural centrum; hspu, haemal spine of preural centrum; hy, hypural; mc, medial cartilage; napu, neural arch of preural centrum; nau, neural arch of ural centrum; nspu, neural spine of preural centrum; nsu, neural spine of ural centrum; ph, parhypural; PU, preural centrum; U, ural centrum; un, uroneural; un*, uroneural fused with the preural centrum (**e**) or compound centrum (**f**). Scale bar =1 mm

Despite their tendency for a delayed ossification in development, alepocephaliforms show an early fusion of heamal spines with their centra anterior to preural centrum 2 (character 3) (Fig. 5b, c). Therefore, character 3 seems to be a valid apomorphy for otomorphs [6, 14, 24]. On the other hand, maybe due to the long persisting cartilages in alepocephaliform development, a continuous cartilaginous basis of hypurals 1 and 2 is clearly visible in *Holtbyrnia* and *Normichthys* (Fig. 5a-c) as well as *Xenodermichthys* and *Maulisia*. This state likely excludes character 4 as possible apomorphy for otomorphs. This cartilage also separates hypural 2 from the compound centrum avoiding a fusion (character 5). However, if the slow ossification sequence in alepocephaliforms is interpreted as apomorphic for this group, the situation in alepoecphaliforms could be the result of a reversal. In summary, the status of characters 4 and 5 remain questionable. The situation is clearer for the pleurostyl (character 6) which is clearly absent in alepocephaliforms (Fig. 5a-c). It is further absent from *Denticeps* (Clupeiformes, Fig. 5d), several fossil clupeiforms [52–54] and gonorynchiforms [25]. All this indicates that pleurostyles in Clupeoidei and Ostariophysi have convergently evolved [13, 14].

### Molecular and morphological evidence for otophysean interrelationships

Figure 3 shows Ostariophysi splitting in two major clades where Gonorynchiformes are sister to the Otophysa (Characiformes, Cypriniformes, Gymnotiformes, and Siluriformes). Within Otophysa, Cypriniformes are sister to a clade comprising Characiformes *Alestes* and *Distichodus* as well as *Gymnotus* sp. representing the Gymnotiformes and *Schilbe* as a representative taxon of Siluriformes. Only recently, the complex phylogeny of this clade has been in the focus of phylogenomic level analyses [32, 33, 55]. A major point of discussion of these studies is the monophyly of Characiformes. While [32, 55] present a phylogeny in congruence to morphological data, [33] do not recover Characiformes as monophyletic.

In our study, the analyses of concatenated amino acid data (Table 1, Additional file 1: Figures S1, S2) reflect the interrelationships of *Gymnotus* and *Schilbe* as sister clade to monophyletic Characiformes. In all other analyses these interrelationships are mixed up showing for example a sister group relationship of *Distichodus* (Characiformes) and *Schilbe* (Siluriformes) or remain unresolved (Additional file 1: Figures S3, S4, S5, S6, S7, S8). We conclude that we did not sample enough variation of these lineages capturing insufficient phylogenetic signal for resolving these interrelationships with confidence. Figure 3 shows a phylogeny derived from the maximum likelihood analysis of concatenated nucleotide alignments, which were identified as significantly more likely compared to other phylogenetic estimates by the AU Test [48–51] (Additional file 1: Table S4). However, only the phylogenetic reconstructions based on the concatenated amino acids (Additional file 1: Figure S1 and S2) align with morphological synapomorphies suggested to characterize these clades. Regarding suggested synapamorphic characters, the Weberian apparatus was considered to characterize the Otophysa as taxonomic unit by Rosen and Greenwood [23] as well as Greenwood [56]. Further, four major clades (i.e. Cypriniformes, Characiformes, Gymnotiformes and Siluriformes) and their interrelationships were strongly supported by several morphological characters considered apomorphic [6, 26]. Contrasting, results of previous molecular phylogenetic studies did not support the monophyly of characiforms [33, 57–61], or the sister-group relation of siluriforms and gymnotiforms, which is strongly supported by several morphological apomorphies [24].

### Phylogeny of Euteleostei

Our analyses clearly recover monophyletic Euteleostei. So far, only three apomorphies were listed in a previous morphological review for the Euteleostei [6]: (1) a stegural, (2) caudal median cartilages, and (3) a unique pattern of supraneural shape and development. The first two characters are challenged by alepocephaliforms clustering among Otomorpha. Alepocephaliforms show a stegural, which is a uroneural 1 with anterodorsal membranous outgrowth [6], and caudal median cartilages (Fig. 5a-c). Presently only the unique supraneural pattern (pattern 2 in Johnson and Patterson [13]) remains as synapomorphic character for the Euteleostei, as alepocephaliforms do not show the respective character state.

The *Lepidogalaxias* lineage forms the sister taxon to all remaining euteleost fishes. This endemic West-Australian freshwater species unites several unique morphological characters and may actually be of key importance to understand the early evolution and extant diversity of euteleosts. Its unexpected phylogenetic position further calls morphological features into question, which were used to characterize interrelationships of *Lepidogalaxias* and galaxiids [29], as these characters are shared between both taxa, although they are showing no close phylogenetic relationship in any molecular phylogenetic analysis ([1–3, 5, 29, 55, 61], this study). This leads us to conclude that some of these characters are based on convergent evolution, while others may indeed be of plesiomorphic state. The phylogenetic position of galaxiids remains enigmatic. While Betancur-R et al. [1] suggest them to be part of the supergroup Protacanthopterygii, Near et al. [5] suggest Galaxiiformes to be the sister to all neoteleost lineages with high node support. Recently, in their phylogenomic level study, Hughes et al. [55] report on some cases of incongruence of gene trees and conflicting phylogenetic hypothesis. Here, Galaxiiformes form the sister group to neoteleosts in all analyses with high support. An estimate ICA value of 0.254 for this split suggests less incongruence compared to 0.078 in [55]. Our results are therefore in favour of Hughes et al.’s [55] hypothesis H1, which may be caused by novel sequencing information from the genus *Lovettia* (Aplochitonidae, respectively Aplochitoninae) representing the sister group of all other galaxiids [62]. Thus we suggest Galaxiiformes to form the sister group to neoeteleost fishes and that thus Protacanthopterygii do not include Galaxiiformes. They are likely of major importance for understanding the evolution of extant Neoteleostei. Development of characters through ontogeny of both lineages are crucial to identify synapomorphies, which may allow for an update for the morphological synapomorphies of neoteleost fishes.

As aforementioned, Protacanthopterygii sensu Betancur-R. et al. [1, 2] cannot be recovered, as Galaxiiformes do not cluster along with other Prothacanthopterygii in any of our analysis. Fig. 3 shows Argentiniformes as sister to a clade comprising Esoci- and Salmoniformes. This sister group relationship cannot always be recovered, as indicated by low node support via bootstrapping as well as low IC values indicating incongruence (Fig. 3, Table 1). The Bayesian inference analysis of the concatenated amino acid alignment contradicts these interrelationships and suggest Argentiniformes as sister to Stomiatii, Galaxiiformes and Neoteleosts (Table 1, Additional file 1: Figure S5). Contrasting, all other analyses of both amino acid and DNA based gene trees align with results from the maximum likelihood analysis from the concatenated DNA and alignment shown in Fig. 3 (Table 1, Additional file 1: Figures S1, S2, S3, S4, S6, S7, S8). Betancur-R. et al. [2] and Hughes et al. [55] discuss the difficult situation for Protacanthopterygii and characterize their classification as *sedis mutabilis* [2]. Due to the very different phylogenetic hypothesis published for the group, morphological evidence supporting the group is virtually lacking. Candidate characters are cartilaginous epicentrals, and simple (not forked) epineurals and epipleurals [6], which are subject of ongoing studies.

Stomiatii sensu Betancur-R. et al. [1, 2] are recovered in all our analyses. The sister group relationship of a mostly coastal marine and coastal freshwater lineage, the Osmeriformes, and an exclusively marine and comparably highly diverse deep-sea lineage, the Stomiatiformes, is noteworthy and somewhat parallels the relationship of Alepocephaliformes and clupeomorphs. Stomiatiformes share morphological characters of neoteleost fishes, prompting synapomorphies for the latter group. These characters mainly refer to the highly derived branchial musculature including a new muscle, the retractor dorsalis, present in Stomiatiformes and neoteleosts [13, 63–65]. Morphological evidence for a relationship of Osmeriformes and Stomiatiformes is presently still lacking. The family Retropinnidae contains several freshwater and brackish water species. Our phylogenetic reconstruction shows that *Retropinna* is sister to all other osmeriforms (Fig. 3). All phylogenetic analyses performed in this study result in a well-supported sister group relationship of Stomiatii to the galaxiiform-neoteleost clade (Table 1, Fig. 3, Additional file 1: Figure S1–8). Morphological synapomorphies for this clade are still lacking.

However, our resolution of some deep phylogenetic nodes within the Clupeocephalan phylogeny will help allowing for reviewing morphological apomorphies and identifying candidate characters for the description and subsequent classification in the context of clades presented in this study.

## Conclusions

Molecular phylogenies including the tree reconstruction presented herein have called numerous morphological apomoprhies of clades into question, as topologies derived from morphology and molecular data differ significantly. The interpretation of suggested morphological synapomorphies of otomorph fishes is strongly affected by the inclusion of deep-sea inhabiting Alepocephaliformes. Our revision of these potential synapomorphies reveals that only a single character of nine characters in total can be flagged as synapomorphy valid for otomorphs. Three further characters remain possible apomorphies since their status cannot be evaluated without ambiguity in Alepocephaliformes.

Our phylogenetic estimate of Euteleost lineages shows that Protacanthopterygii, sensu Betancur-R. et al. [2] comprising Esoci-, Salmoni-, Argentini-, and Galaxiiformes, cannot be recovered, as Galaxiiformes appear to be the sister to all neoteleost fishes and further, a common ancestor of Salmoni-, Esoci and Argentiniformes is not well supported. It is noteworthy that another study contemporaneously aiming to solve the actinopterygian phylogeny with a very similar approach as the analysis presented herein, results in the same challenging nodes in their phylogenetic estimate [55]. Some splits remain uncertain, as e.g. the composition and phylogenetic placement of Protacanthopterygii or the phylogenetic placement of Alepocephaliformes, a group not included in [55]. Our efforts to reconstruct deep phylogenetic nodes based on a phylogenomic level dataset of clupeocephalan fishes still reveals problematic divergence estimates and calls for the application of phylogenomic methods on datasets with an enhanced taxon sampling, which can strengthen some of the phylogenetic hypothesis presented herein. Regarding morphology, data on possible characters for several nodes in the present clupeocephalan tree are scarce. Therefore, subsequent morphological studies are required to understand character evolution, evolutionary driving forces and origin of species diversity in extant clupeocephalans.

## Methods

### Material

The major part of samples was collected during field trips and comprise muscle tissue or fin clips, respectively. The sampling aims for covering the major teleost lineages with a focus on Clupeocephala where neoteleosts, elopomorphs and osteoglossmorphs serve as an outgroup. Please see Additional file 1: Table S1 for an overview of samples analysed.

### Methods

#### Bait design

To retrieve blueprints for bait sequences, we used the online resource Evolmarkers [66, 67] to search for putative ortholog exon loci in publicly available reference genomes. In a first step, we searched the genome of the zebra fish, *Danio rerio* (Cypriniformes), for single-copy loci using standalone BLAST [34]. In a second step, the results were subsequently BLASTed [34] against further available bony fish genomes, at the time of bait design comprising *Anguilla anguilla*, *Oryzias latipes*, *Tetraodon nigroviridis*, *Lepisosteus oculatus*, *Gadus morhua*, *Gasterosteus aculeatus* and *Oreochromis niloticus*. Finally, only exon sequences with a single BLAST [34] hit in all analysed genomes were used for bait design. Custom RNA baits were manufactured by Arbor Biosciences (Ann Arbor, Michigan, USA) with a length of 120 nucleotides and 60 nucleotides overlap after padding sequence lengths totaling 39,049 unfiltered baits with a 2× flexible tiling density. After removing all baits with any soft-masked sequence, 38,318 baits were put into production.

#### Library preparation

Genomic DNA was extracted from tissue samples listed in Additional file 1: Table S1 using the Machery & Nagel blood and tissue kit®. The DNA content of the final eluate was measured using a Qbit® Fluorometer (Life Technologies) applying the broad range kit. Thereafter, 130 μl with a concentration of at least 3 ng/μl DNA were used for shearing the DNA to ~ 500 bp using a Covaris® Sonicator. Shearing success was checked using gel-electrophoresis. The following steps for Illumina (Illumina, Inc., San Diego, CA) library constructions are based on Li et al. [68] and comprise a size selection step for fragments > 500 bp, blunt end repair using polymerase, adaptor ligation, fill-in and a final amplification of libraries using the KAPA® library amplification kit. DNA content of libraries was measured using a Qbit® fluorometer applying the high sensitivity kit and further checked with gel-electrophoresis to check the size distribution of fragments.

#### Target capture

For the performance of interordinal target capture, amplified libraries from step 2.2 served as starting point for hybrid enrichment. All steps follow the protocol provided in Li et al. [68]. Summarizing, library fragments are hybridized to RNA baits, remaining fragments and unintentionally hybridized fragments are washed off. During hybridisation, blocking oligos are preventing adaptor to adaptor ligation, while human cot DNA serves to avoid repetitive elements to cause non-specific binding. We applied a touch down hybridisation with decreasing hybridisation temperature from 65° to 50 °C in steps of 11 h totaling 36 h of hybridisation. The captured library is again amplified, size selected [69] and used as a starting point for a second round of capture, which is shown to increase the number of genes captured [68, 70].

During the final amplification step, individual sequencing indices are implemented to the adaptors allowing for de-multiplexing of reads after sequencing on an Illumina MiSeq® instrumentation. We aimed for an average coverage of 6.6 million paired-end 250 basepair reads per sample.

#### Data analysis

##### Recovery of exon sequence alignments of phylogenetically informative loci

Sequencing reads were first checked for quality and low quality reads were excluded from further analysis with a cut-off value of 20. Adaptors were trimmed from reads using Trimgalore vers 03.07 [71, 72]. Thereafter, we followed the analysis pipeline introduced for target capture data in Yuan et al. [37]. Trimmed reads are first searched for replicate sequences, which are subsequently removed. For that, the first 20 bp of both reads are compared, if identical, they are removed. In a next step, the reads are BLASTed [34] against the bait sequences to sort the reads into corresponding gene bins. Next, reads are *de-novo* assembled into contigs using Trinity vers. 2.2.0 [73]. Output contigs are then separated into folders containing one or more than one contig. Where Trinity was creating more than a single contig sequence, Geneious® R7 was used to further assemble multiple contigs in an effort to create even longer contig sequences. For retrieving the best sequences of each gene in comparison to the bait sequences (query sequences were the bait sequences derived from the *Danio rerio* genome), we predicted the frame of each query sequence using a custom python script (predict.frame.py), which is available for download at the Dryad data depository [39], and trimmed stop codons from it. Subsequently, contigs were translated into amino acids. All contigs were reciprocally blasted against the query sequences to check for homologs, i.e. contigs showing the best blast hit out of the target region were excluded from further analytical steps. As we are performing target capture on inter-ordinal level, the rate of gene losses or duplications is unknown, therefore single copy genes identified in Evolmarkers [66, 67] are not necessarily single copy in phylogenetically distant taxa. Therefore, contigs, which did not pass the reciprocal blast screening, were excluded from further analyses. Finally, intron inserted sequences were merged and subsequently translated to amino acids. We used customized Perl scripts to batch align each gene bin file containing all captured taxa and the bait sequence using MAFFT [74, 75]. As cross-contamination poses a problem in NGS datasets (e.g. [76]), possible cross-contamination were checked using a custom perl-script [39], which uses p-distances computed from single loci alignments and information on taxon groups assumed to be closely related. These are then compared with more distantly related taxa. Potential cross-contamination is indicated by extremely small p-distances (equal to or smaller than 0.002) between distantly related taxa (Additional file 1: Table S2). Although p-distance between conserved loci can be extremely small between distantly related taxa, conservation cannot be ubiquitous among all loci. Thus, there is no cross-contamination between a pair of taxa, if percentage of potential cross-contamination between them among all loci is extremely low. Subsequently, cleaned single loci were concatenated using Geneious® R7. The concatenated sequences were checked for phylogenetically most informative loci using the software Matrix Reduction [35], which is based on the treelikeness computed for single loci [36]. After extracting 838 most informative loci [39], we used customized Perl scripts to batch align each gene bin file containing all captured taxa and the bait sequence using MAFFT [74, 75]. As an additional check for orthology, the 838 loci identified with the Matrix reduction software, were analysed using Orthograph vers. 0–6–3-1 [38] and the custom script reblast.pl (Additional file 1: Table S3). Applying the latter approach, another four loci were eliminated from the dataset. Finally, five loci were excluded as they contained less than four sequences.

### Phylogenetic analyses

#### Concatenated datasets

Concatenation was performed in Geneious R7 on 829 phylogenetically informative loci suggested from the MARE [35, 36] analysis (Additional file 1). We analysed two datasets, i.e. the amino acid alignments as well as the DNA alignments. For finding best-fitting partitions for the concatenated datasets, Partition finder vers. 2.1.1 [40–42] was used. A phylogenetic analysis based on maximum likelihood was performed in RAxML vers. 8.2.4 [42] incorporating the best-fitting partitioning schemes. RAxML [42] settings were applying the GTR GAMMA substitution model. Bootstrapping was halted automatically [77] using the fast hill-climbing algorithm.

As an alternative, we computed a phylogenetic tree using a Bayesian inference applying the CAT dirichlet process [78, 79] implemented in PhyloBayes vers. 4.1c [43]. Two chains were run in parallel and checked for convergence using the tracecomp and bpcomp scripts provided in PhyloBayes.

All abovementioned analyses were performed in CIPRES [80].

#### Coalescence analysis

For a comparison of phylogenies computed from the concatenated dataset and a coalescence-based approach, we further performed maximum likelihood tree searches on single DNA and amino acid loci alignments using RAxML [42] on batch for attaining a collection of gene trees from both amino acid and DNA datasets. Those were subsequently used for estimating the species tree in ASTRAL vers. 4.10.12 [44].

#### Computing tree certainty and performing AU test

We computed internode certainty (IC/ ICA) and tree certainty (TC/ TCA) values [45, 46] from partial gene trees from the gene trees depicted from the coalescence analysis as implemented in RAxML [42, 47] using the best tree resulting from the maximum likelihood analysis of the concatenated and best-partitioned amino acid alignments. This step was used to evaluate incongruence among trees.

For testing for significant differences of species trees and fully bifurcating trees based on concatenated alignments, we performed an AU test in CONSEL [48–51].

#### Morphological comparative material

Cleaned and double stained collection specimen:

Osteoglossiformes. Osteoglossidae: *Osteoglossum bicirrhosum* (Cuvier, 1829): DMM IE/11035, 95.5 mm SL.

Elopiformes. Elopidae: *Elops senegalensis* Regan, 1909: DMM IE/11008, 61.3 mm SL.

Clupeiformes. Denticipitidae: 3 *Denticeps clupeoides* Clausen, 1959: DMM IE/11417, IE11420, 29.2–41.1 mm SL. *Clupea harengus* Linnaeus, 1758: DMM IE/ 11,029 83.1 mm SL.

Alepocephaliformes. Alepocephalidae: *Alepocephalus bicolor* Alcock, 1891: DMM IE/9602, 192 mm SL, *Xenodermichthys copei* (Gill, 1884) DMM IE/10190, 110.1 mm SL. Platytroctidae: 5 *Holtbyrnia anomala* Krefft, 1980: DMM IE/10079, IE 10079, IE 6145, IE 4885, 55.99 mm - 144.4 mm SL; *Maulisia argipalla* Matsui & Rosenblatt, 1979: DMM IE/10459, 115.6 mm SL. *Normichthys operosus* Parr, 1951, DMM IE/11040, 97.1 mm SL; *Searsia koefoedi* Parr 1937: DMM IE/10191, 117.6 mm SL.

Gonorynchiformes. Gonorynchidae: *Gonorynchus abbreviates* Temminck & Schlegel, 1846, DMM IE/11730, 84,2 mm SL; Chanidae: *Chanos chanos* (Forsskl, 1775): DMM IE/11010, 72.18 mm SL; Kneridae: *Kneria stappersii* Boulenger, 1915*,* DMM IE/12025, 26.4 mm SL.

Cypriniformes. Cyprinidae: 2 *Dawkinsia tambraparniei* (Silas, 1954): DMM IE/12072, 27.8 mm SL, 28.6 mm SL.

Argentiniformes. Argentinidae: *Argentina silus* (Ascanius, 1775): DMM IE/11033, 103.2 mm SL; Bathylagidae: *Bathylagus euryops* Goode & Bean, 1896: DMM IE/11034, 96.3 mm SL.

Osmeriformes. Osmeridae: *Osmerus eperlanus* (Linnaeus, 1758), DMM IE/11090, 36.5 mm SL.

Salmoniformes: Thymallidae: *Thymallus thymallus* (Linnaeus, 1758) DMM IE/11820, 99.5 mm SL.

The specimens were cleared and double stained following [81, 82]. Specimens were transferred into 98% ethanol. Afterwards, cartilage was stained with Alican blue in 1:4 acetic acid and ethanol-solution for maximally 48 h. Thereafter, the specimens were transferred via a decreasing alcohol concentration in digestion solution with trypsin. As soon as specimens were cleared, the pigmentation of the skin was eliminated by a bleach bath of potassium hydroxide solution and addition of hydrogen peroxide. Subsequently, bones were stained with Alizarin-red. Finally, specimens were transferred into glycerine for increasing the transparency.

The dissected parts of the specimens were photographed with a Canon EOS 50D with a Sigma 105 mm lens and the software EOS Utility 3.0 (Canon). Stacking of images for obtaining advanced and extended focus images were done with software Helicon Focus 6. The images were edited in GIMP 2.8 and were compiled in Inkscape 0.92.1.

## Additional file

Additional file 1: Overview of samples, analyses protocols, and tree Figures S1-S8. (PDF 772 kb)
