# Supplementary material for: A phylogenomic approach to reconstruct interrelationships of main clupeocephalan lineages with a critical discussion of morphological apomorphies
Source: BMC Evol Biol. 2018 Oct 23;18:158. doi: 10.1186/s12862-018-1267-1 (PMC6199709; doi:10.1186/s12862-018-1267-1)

Additional file 1: Analyses protocols & detailed results

Table S1: Overview of samples analysed. DMM: Deutsches Meeresmuseum; ZSM: Zoologische Staatssammlung München; BPS: Musée d'Histoire naturelle.

| **Voucher specimen** | ***Species*** | # reads (x10000) | # contigs | # genes captured |
|---|---|---|---|---|
| DMM IE/9239 | *Elops senegalensis* | 490 | 1711 | 1044 |
| DMM IE/9474 | *Pantodon buchholzi* | 380 | 4731 | 3179 |
| DMM IE/11506 | *Denticeps clupeoides* | 340 | 4071 | 2886 |
| DMM IE/9760 | *Dussumieria elopsoides* | 240 | 2162 | 1477 |
| DMM IE/4611 | *Clupea harengus* | 510 | 1612 | 974 |
| DMM IE/9418 | *Pellonula leonensis* | 500 | 1358 | 891 |
| ZSM-PIS-044562 | *Limnothrissa miodon* | 1060 | 4430 | 3077 |
| DMM IE/6170 | *Engraulis encrassicolus* | 820 | 3546 | 2410 |
| DMM IE/9671 | *Alepocephalus bicolor* | 200 | 2240 | 1659 |
| DMM IE/5805 | *Alepocephalus brevipinni* | 600 | 5002 | 3814 |
| n.a. | *Holtbyrnia anomala* | 530 | 640 | 353 |
| DMM IE/10166 | *Cromeria occidentalis* | 1800 | 5700 | 3938 |
| DMM IE/9985 | *Chanos chanos* | 170 | 2878 | 2019 |
| DMM IE/9517 | *Gonorynchus abbreviatus* | 230 | 2969 | 2137 |
| ZSM32021 | *Gymnotus* sp. | 200 | 2273 | 2076 |
| DMM IE/6305 | *Alestes baremoze* | 800 | 3999 | 2977 |
| DMM IE/10925 | *Schilbe mystus* | 320 | 1485 | 617 |
| DMM IE/9036 | *Distichodus brevipinnis* | 420 | 735 | 449 |
| n.a. | *Anguilla anguilla* | 800 | 5181 | 3769 |
| DMM IE/5966a | *Rutilus rutilus* | 470 | 5441 | 4513 |
| DMM IE/5966b | *Rutilus rutilus* | 720 | 9693 | 8290 |
| DMM IE/12383 | *Danio rerio* | 720 | 11676 | 10929 |
| SAM-FISHY6:PU09-49LS-1 | *Lepidogalaxias salamandroides* | 1350 | 6228 | 4315 |
| DMM IE/15076 | *Esox lucius* | 190 | 344 | 185 |
| UW150693 | *Dallia pectoralis* | 780 | 3792 | 2502 |
| DMM IE/6000 | *Umbra krameri* | 910 | 3118 | 2178 |
| DMM IE/10013 | *Salmo trutta* | 580 | 2019 | 1298 |
| ZSM40048 | *Coregonus* sp. | 800 | 4410 | 5664 |
| DMM IE/5848 | *Argentina sphyraena* | 500 | 2915 | 1952 |
| BPS0624 | *Opisthoproctus soleatus* | 800 | 5096 | 3586 |
| BPS1997 | *Nansenia oblita* | 740 | 4126 | 2738 |
| DMM IE/4896 | *Bathylagus euryops* | 850 | 3124 | 2023 |
| DMM IE/6150 | *Borostomias antarcticus* | 460 | 3537 | 2430 |
| DMM IE/12416 | *Astronesthes lucifer* | 390 | 2505 | 1702 |
| DMM IE/12433 | *Maurolicus muelleri* | 490 | 1740 | 1098 |
| DMM IE/12145 | *Retropinna tasmanica* | 3200 | 6439 | 3482 |
| CL284 | *Retropinna tasmanica* | 890 | 4270 | 2904 |

| | | | | |
|---|---|---|---|---|
| DMM IE/6055 | *Mallotus villosus* | 870 | 5879 | 4036 |
| DMM IE/6198 | *Osmerus eperlanus* | 690 | 2452 | 1582 |
| DMM IE/12147 | *Lovettia sealii* | 800 | 6190 | 3962 |
| DMM IE/12429 | *Salanx acuticeps* | 330 | 3122 | 2074 |
| DMM IE/9685 | *Plecoglossus altivelis* | 240 | 2047 | 1795 |
| CL282 | *Galaxias fasciatus* | 1000 | 4356 | 2918 |
| DMM IE/5811 | *Evermanella balbo* | 750 | 2732 | 1829 |
| DMM IE/12414 | *Lestrolepis japonica* | 270 | 1800 | 1089 |
| DMM IE/4644 | *Arctozensus risso* | 550 | 1735 | 1089 |
| DMM IE/6171 | *Synodus saurus* | 590 | 2758 | 1901 |
| DMM IE/9594 | *Hime japonica* | 330 | 2198 | 1467 |
| DMM IE/9556 | *Harpadon microchir* | 200 | 1899 | 1253 |
| DMM IE/9556 | *Ateleopus japonicus* | 190 | 1813 | 1198 |
| BPS2503 | *Ijimaia loppei* | 280 | 2438 | 1598 |
| DMM IE/6653 | *Trachypterus trachipterus* | 390 | 757 | 400 |

Table S2: Results from cross contamination check using detect_contamination.pl

| Contaminated sample[1] | Contaminating source[2] | Percentage of contamination(%)[3] | Contaminated pair[4] | All pair[5] | Contaminating_source->Contaminated_sample[6] | Contaminated_sample->Contaminating_source[7] |
|---|---|---|---|---|---|---|
| Anguilla_anguillatm125 | Alepocephalus_brevipinnitm66merge | 0.23 | 4 | 1770 | 1 | 0 |
| Coregonus_spZSM13380 | Argentina_sphyraena029tm | 0.1 | 1 | 980 | 0 | 0 |
| Cromeria_occidentalistm583a | Bathylagus_euryopstm112 | 0.11 | 1 | 938 | 0 | 0 |
| Danio_rerioD15 | Alepocephalus_bicolor | 0.08 | 1 | 1310 | 1 | 0 |
| Alepocephalus_brevipinnitm66merge | Danio_rerioD15 | 1.2 | 38 | 3174 | 4 | 1 |
| Anguilla_anguillatm125 | Danio_rerioD15 | 1.44 | 45 | 3119 | 3 | 0 |
| Danio_rerioD15 | Borostomias_antarcticustm762 | 0.05 | 1 | 1962 | 0 | 0 |
| Galaxias_fasciatusCL282 | Coregonus_spZSM13380 | 0.08 | 1 | 1241 | 0 | 0 |
| Engraulis_encrassicolustm57 | Galaxias_fasciatusCL282 | 0.11 | 1 | 914 | 1 | 0 |
| Gonorynchus_abbreviatus098tm | Alepocephalus_brevipinnitm66merge | 0.08 | 1 | 1189 | 0 | 0 |
| Gonorynchus_abbreviatus098tm | Borostomias_antarcticustm762 | 0.12 | 1 | 842 | 0 | 0 |
| Gonorynchus_abbreviatus098tm | Danio_rerioD15 | 0.06 | 1 | 1712 | 1 | 0 |
| Gonorynchus_abbreviatus098tm | Dussumiera_elopsoides037tm | 0.17 | 1 | 603 | 1 | 0 |
| Gymnotus_spZSM32021 | Borostomias_antarcticustm762 | 0.14 | 1 | 694 | 0 | 0 |
| Gymnotus_spZSM32021 | Danio_rerioD15 | 0.06 | 1 | 1689 | 0 | 0 |
| Gymnotus_spZSM32021 | Galaxias_fasciatusCL282 | 0.12 | 1 | 817 | 0 | 0 |
| Gymnotus_spZSM32021 | Gonorynchus_abbreviatus098tm | 0.15 | 1 | 673 | 0 | 0 |

| | | | | | | |
|---|---|---|---|---|---|---|
| Hime_formosana137tm | Borostomias_antarcticustm762 | 0.15 | 1 | 656 | 0 | 0 |
| Hime_formosana137tm | Danio_rerioD15 | 0.09 | 1 | 1170 | 0 | 0 |
| Hime_formosana137tm | Gonorynchus_abbreviatus098tm | 0.17 | 1 | 583 | 0 | 0 |
| Hime_formosana137tm | Gymnotus_spZSM32021 | 0.21 | 1 | 480 | 0 | 0 |
| Lepidogalaxias_salamandroidesSAM | Bathylagus_euryopstm112 | 0.09 | 1 | 1072 | 0 | 0 |
| Lepidogalaxias_salamandroidesSAM | Coregonus_spZSM13380 | 0.12 | 2 | 1631 | 0 | 0 |
| Lepidogalaxias_salamandroidesSAM | Cromeria_occidentalistm583a | 0.1 | 2 | 1960 | 0 | 0 |
| Lepidogalaxias_salamandroidesSAM | Engraulis_encrassicolustm57 | 0.08 | 1 | 1234 | 0 | 0 |
| Lepidogalaxias_salamandroidesSAM | Galaxias_fasciatusCL282 | 0.06 | 1 | 1578 | 0 | 0 |
| Limnothrissa_miodon | Bathylagus_euryopstm112 | 0.12 | 1 | 854 | 0 | 0 |
| Limnothrissa_miodon | Lepidogalaxias_salamandroidesSAM | 0.06 | 1 | 1629 | 0 | 0 |
| Lovettia_sealii_CSIROtr | Bathylagus_euryopstm112 | 0.09 | 1 | 1054 | 0 | 0 |
| Lovettia_sealii_CSIROtr | Lepidogalaxias_salamandroidesSAM | 0.05 | 1 | 2142 | 0 | 0 |
| Mallotus_villosustm016 | Alepocephalus_bicolor | 0.11 | 1 | 893 | 0 | 0 |
| Mallotus_villosustm016 | Alepocephalus_brevipinnitm66merge | 0.06 | 1 | 1798 | 0 | 0 |
| Mallotus_villosustm016 | Bathylagus_euryopstm112 | 0.09 | 1 | 1062 | 0 | 0 |
| Mallotus_villosustm016 | Borostomias_antarcticustm762 | 0.14 | 2 | 1413 | 0 | 0 |
| Mallotus_villosustm016 | Clupea_harengustm100 | 0.19 | 1 | 538 | 0 | 0 |
| Mallotus_villosustm016 | Danio_rerioD15 | 0.03 | 1 | 3326 | 0 | 0 |
| Gonorynchus_abbreviatus098tm | Mallotus_villosustm016 | 0.42 | 5 | 1188 | 3 | 0 |
| Mallotus_villosustm016 | Gymnotus_spZSM32021 | 0.1 | 1 | 989 | 0 | 0 |

| | | | | | | |
|---|---|---|---|---|---|---|
| Mallotus_villosustm016 | Hime_formosana137tm | 0.12 | 1 | 857 | 0 | 0 |
| Maurolicus_muelleritm2013W | Mallotus_villosustm016 | 0.3 | 2 | 661 | 0 | 0 |
| Nansenia_oblitaBPS1997 | Mallotus_villosustm016 | 0.07 | 1 | 1431 | 0 | 0 |
| Opisthoproctus_soleatusBPS0624 | Alepocephalus_brevipinnitm66merge | 0.12 | 2 | 1728 | 0 | 0 |
| Opisthoproctus_soleatusBPS0624 | Alestes_baremozetm285 | 0.07 | 1 | 1334 | 0 | 0 |
| Opisthoproctus_soleatusBPS0624 | Anguilla_anguillatm125 | 0.12 | 2 | 1675 | 0 | 0 |
| Opisthoproctus_soleatusBPS0624 | Coregonus_spZSM13380 | 0.07 | 1 | 1515 | 0 | 0 |
| Cromeria_occidentalistm583a | Opisthoproctus_soleatusBPS0624 | 0.12 | 2 | 1715 | 1 | 0 |
| Opisthoproctus_soleatusBPS0624 | Danio_rerioD15 | 0.74 | 22 | 2957 | 3 | 0 |
| Dussumiera_elopsoides037tm | Opisthoproctus_soleatusBPS0624 | 0.13 | 1 | 767 | 1 | 0 |
| Osmerus_eperlanustm785 | Bathylagus_euryopstm112 | 0.2 | 1 | 494 | 0 | 0 |
| Osmerus_eperlanustm785 | Borostomias_antarcticustm762 | 0.14 | 1 | 721 | 0 | 0 |
| Osmerus_eperlanustm785 | Danio_rerioD15 | 0.08 | 1 | 1254 | 0 | 0 |
| Esox_luciustm052 | Osmerus_eperlanustm785 | 5.56 | 4 | 72 | 2 | 0 |
| Gonorynchus_abbreviatus098tm | Osmerus_eperlanustm785 | 0.18 | 1 | 569 | 1 | 0 |
| Osmerus_eperlanustm785 | Gymnotus_spZSM32021 | 0.22 | 1 | 464 | 0 | 0 |
| Osmerus_eperlanustm785 | Hime_formosana137tm | 0.22 | 1 | 446 | 0 | 0 |
| Osmerus_eperlanustm785 | Maurolicus_muelleritm2013W | 0.27 | 1 | 377 | 0 | 0 |
| Osmerus_eperlanustm785 | Nansenia_oblitaBPS1997 | 0.3 | 2 | 669 | 0 | 0 |

| | | | | | | |
|---|---|---|---|---|---|---|
| Retropinna_tasmanicaCL284a | Borostomias_antarcticustm762 | 0.08 | 1 | 1198 | 0 | 0 |
| Retropinna_tasmanicaCL284a | Chanos_chanostm1777 | 0.11 | 1 | 921 | 0 | 0 |
| Retropinna_tasmanicaCL284a | Danio_rerioD15 | 0.04 | 1 | 2383 | 0 | 0 |
| Retropinna_tasmanicaCL284a | Galaxias_fasciatusCL282 | 0.08 | 1 | 1266 | 0 | 0 |
| Gonorynchus_abbreviatus098tm | Retropinna_tasmanicaCL284a | 0.11 | 1 | 946 | 1 | 0 |
| Retropinna_tasmanicaCL284a | Gymnotus_spZSM32021 | 0.13 | 1 | 796 | 0 | 0 |
| Retropinna_tasmanicaCL284a | Hime_formosana137tm | 0.13 | 1 | 749 | 0 | 0 |
| Retropinna_tasmanicaCL284a | Lovettia_sealii_CSIROtr | 0.06 | 1 | 1551 | 0 | 0 |
| Retropinna_tasmanicaCL284a | Mallotus_villosustm016 | 0.12 | 2 | 1727 | 0 | 0 |
| Nansenia_oblitaBPS1997 | Retropinna_tasmanicaCL284a | 0.09 | 1 | 1169 | 1 | 0 |
| Retropinna_tasmanicaCL284a | Osmerus_eperlanustm785 | 0.23 | 2 | 884 | 0 | 0 |
| Retropinna_tasmanica_CSIROtr | Borostomias_antarcticustm762 | 0.07 | 1 | 1396 | 0 | 0 |
| Retropinna_tasmanica_CSIROtr | Chanos_chanostm1777 | 0.09 | 1 | 1116 | 0 | 0 |
| Retropinna_tasmanica_CSIROtr | Danio_rerioD15 | 0.03 | 1 | 3754 | 0 | 0 |
| Galaxias_fasciatusCL282 | Retropinna_tasmanica_CSIROtr | 0.06 | 1 | 1619 | 1 | 0 |
| Gonorynchus_abbreviatus098tm | Retropinna_tasmanica_CSIROtr | 0.09 | 1 | 1148 | 1 | 0 |
| Retropinna_tasmanica_CSIROtr | Gymnotus_spZSM32021 | 0.08 | 1 | 1258 | 0 | 0 |
| Retropinna_tasmanica_CSIROtr | Hime_formosana137tm | 0.11 | 1 | 880 | 0 | 0 |

| | | | | | | |
|---|---|---|---|---|---|---|
| Retropinna_tasmanica_CSIROtr | Lovettia_sealii_CSIROtr | 0.24 | 6 | 2514 | 0 | 0 |
| Retropinna_tasmanica_CSIROtr | Mallotus_villosustm016 | 0.09 | 2 | 2267 | 0 | 0 |
| Nansenia_oblitaBPS1997 | Retropinna_tasmanica_CSIROtr | 0.07 | 1 | 1474 | 1 | 0 |
| Retropinna_tasmanica_CSIROtr | Osmerus_eperlanustm785 | 0.21 | 2 | 952 | 0 | 0 |
| Alepocephalus_brevipinnitm66merge | Rutilus_rutilus2 | 0.69 | 13 | 1881 | 1 | 0 |
| Anguilla_anguillatm125 | Rutilus_rutilus2 | 1.02 | 18 | 1762 | 5 | 0 |
| Bathylagus_euryopstm112 | Rutilus_rutilus2 | 0.9 | 9 | 999 | 3 | 0 |
| Rutilus_rutilus2 | Borostomias_antarcticustm762 | 0.25 | 3 | 1209 | 0 | 0 |
| Rutilus_rutilus2 | Cromeria_occidentalistm583a | 0.27 | 5 | 1849 | 0 | 0 |
| Esox_luciustm052 | Rutilus_rutilus2 | 3 | 3 | 100 | 1 | 0 |
| Gonorynchus_abbreviatus098tm | Rutilus_rutilus2 | 0.18 | 2 | 1133 | 1 | 0 |
| Rutilus_rutilus2 | Gymnotus_spZSM32021 | 0.1 | 1 | 1029 | 0 | 0 |
| Rutilus_rutilus2 | Hime_formosana137tm | 0.13 | 1 | 778 | 0 | 0 |
| Rutilus_rutilus2 | Lepidogalaxias_salamandroidesSAM | 0.36 | 7 | 1942 | 0 | 0 |
| Limnothrissa_miodon | Rutilus_rutilus2 | 0.66 | 10 | 1504 | 3 | 0 |
| Rutilus_rutilus2 | Mallotus_villosustm016 | 0.06 | 1 | 1770 | 0 | 0 |
| Opisthoproctus_soleatusBPS0624 | Rutilus_rutilus2 | 0.64 | 11 | 1723 | 2 | 0 |
| Rutilus_rutilus2 | Osmerus_eperlanustm785 | 0.13 | 1 | 773 | 0 | 0 |
| Rutilus_rutilus2 | Retropinna_tasmanicaCL284a | 0.07 | 1 | 1363 | 0 | 0 |
| Rutilus_rutilus2 | Retropinna_tasmanica_CSIROtr | 0.05 | 1 | 1874 | 0 | 0 |
| Rutilus_rutilustm70 | Alepocephalus_bicolor | 0.09 | 1 | 1098 | 1 | 0 |

| | | | | | | |
|---|---|---|---|---|---|---|
| Rutilus_rutilustm70 | Alepocephalus_brevipinnitm66merge | 0.65 | 17 | 2628 | 2 | 2 |
| Anguilla_anguillatm125 | Rutilus_rutilustm70 | 0.58 | 15 | 2579 | 6 | 1 |
| Bathylagus_euryopstm112 | Rutilus_rutilustm70 | 0.58 | 8 | 1389 | 3 | 0 |
| Rutilus_rutilustm70 | Borostomias_antarcticustm762 | 0.24 | 4 | 1652 | 0 | 0 |
| Cromeria_occidentalistm583a | Rutilus_rutilustm70 | 0.22 | 6 | 2788 | 1 | 0 |
| Esox_luciustm052 | Rutilus_rutilustm70 | 1.77 | 2 | 113 | 1 | 0 |
| Gonorynchus_abbreviatus098tm | Rutilus_rutilustm70 | 0.14 | 2 | 1451 | 1 | 0 |
| Rutilus_rutilustm70 | Gymnotus_spZSM32021 | 0.07 | 1 | 1461 | 0 | 0 |
| Rutilus_rutilustm70 | Hime_formosana137tm | 0.1 | 1 | 1006 | 0 | 0 |
| Rutilus_rutilustm70 | Ijimaia_loppeiBPS2503 | 0.09 | 1 | 1090 | 0 | 0 |
| Rutilus_rutilustm70 | Lepidogalaxias_salamandroidesSAM | 0.13 | 4 | 3007 | 0 | 0 |
| Limnothrissa_miodon | Rutilus_rutilustm70 | 0.37 | 8 | 2175 | 1 | 0 |
| Rutilus_rutilustm70 | Mallotus_villosustm016 | 0.04 | 1 | 2753 | 0 | 0 |
| Opisthoproctus_soleatusBPS0624 | Rutilus_rutilustm70 | 0.28 | 7 | 2475 | 2 | 0 |
| Rutilus_rutilustm70 | Osmerus_eperlanustm785 | 0.1 | 1 | 1046 | 0 | 0 |
| Rutilus_rutilustm70 | Retropinna_tasmanicaCL284a | 0.05 | 1 | 1968 | 0 | 0 |
| Rutilus_rutilustm70 | Retropinna_tasmanica_CSIROtr | 0.03 | 1 | 3083 | 0 | 0 |
| Salanx_acuticepstmT02 | Ateleopus_japonicus133tm | 0.2 | 1 | 509 | 0 | 0 |
| Salanx_acuticepstmT02 | Borostomias_antarcticustm762 | 0.11 | 1 | 870 | 0 | 0 |
| Harpadon_microchir067tm | Salanx_acuticepstmT02 | 0.78 | 4 | 512 | 2 | 0 |
| Salanx_acuticepstmT02 | Maurolicus_muelleritm2013W | 0.22 | 1 | 448 | 0 | 0 |

| | | | | | | |
|---|---|---|---|---|---|---|
| Salanx_acuticepstmT02 | Retropinna_tasmanicaCL284a | 0.19 | 2 | 1052 | 0 | 0 |
| Salanx_acuticepstmT02 | Retropinna_tasmanica_CSIROtr | 0.08 | 1 | 1255 | 0 | 0 |
| Salmo_truttatm20154 | Alestes_baremozetm285 | 0.18 | 1 | 546 | 0 | 0 |
| Salmo_truttatm20154 | Anguilla_anguillatm125 | 0.15 | 1 | 657 | 0 | 0 |
| Salmo_truttatm20154 | Lovettia_sealii_CSIROtr | 0.15 | 1 | 684 | 0 | 0 |
| Salmo_truttatm20154 | Retropinna_tasmanica_CSIROtr | 0.14 | 1 | 740 | 0 | 0 |
| Salmo_truttatm20154 | Schilbe_mystustm551b | 36.9 | 69 | 187 | 8 | 4 |
| Umbra_krameritm62 | Alepocephalus_brevipinnitm66merge | 0.26 | 3 | 1157 | 0 | 0 |
| Umbra_krameritm62 | Anguilla_anguillatm125 | 0.27 | 3 | 1094 | 0 | 0 |
| Umbra_krameritm62 | Bathylagus_euryopstm112 | 0.15 | 1 | 682 | 0 | 0 |
| Umbra_krameritm62 | Danio_rerioD15 | 3.24 | 56 | 1727 | 1 | 0 |
| Umbra_krameritm62 | Limnothrissa_miodon | 0.1 | 1 | 997 | 0 | 0 |
| Umbra_krameritm62 | Mallotus_villosustm016 | 0.09 | 1 | 1142 | 0 | 0 |
| Umbra_krameritm62 | Nansenia_oblitaBPS1997 | 0.11 | 1 | 892 | 0 | 0 |
| Umbra_krameritm62 | Opisthoproctus_soleatus BPS0624 | 0.18 | 2 | 1139 | 0 | 0 |
| Umbra_krameritm62 | Osmerus_eperlanustm785 | 0.18 | 1 | 548 | 0 | 0 |
| Umbra_krameritm62 | Retropinna_tasmanicaCL284a | 0.1 | 1 | 967 | 0 | 0 |
| Umbra_krameritm62 | Rutilus_rutilus2 | 2.85 | 33 | 1157 | 1 | 0 |
| Umbra_krameritm62 | Rutilus_rutilustm70 | 1.75 | 26 | 1484 | 1 | 0 |

[1] Name of contaminated sample
[2] Name of contaminating source
[3] Divide times of occurrence of contamination by times of occurrence of contaminated sample-contaminating source pair among all loci (x 100)
[4] Times of occurrence of contamination between contaminated sample-contaminating source pair among all loci
[5] Times of occurrence of contaminated sample-contaminating source pair among all loci
[6] Times of contaminating source contaminated contaminated sample
[7] Times of contaminated sample contaminated contaminating source

Table S3: Results from orthology check on additional loci using Orthograph [72].

| **Locus[1]** | **Orthology Group[2]** | **Reciprocal blast hit (RBH) samples[3]** | **Enriched samples[4]** | **Reason of samples without RBH** |
|---|---|---|---|---|
| Danio_rerio.1.18296121.18296321 | Danio_rerio.1.18296121.18296321 | 33 | 33 | |
| Danio_rerio.1.18299488.18299648 | Danio_rerio.1.18299488.18299648 | 21 | 21 | |
| Danio_rerio.1.27027674.27028223 | Danio_rerio.1.27027674.27028223 | 22 | 22 | |
| Danio_rerio.1.28199081.28201810 | Danio_rerio.1.28199081.28201810 | 25 | 26 | too short: Trachypterus_trachipterustm119 |
| Danio_rerio.1.31116778.31116951 | Danio_rerio.1.31116778.31116951 | 27 | 27 | |
| Danio_rerio.1.35295365.35295668 | Danio_rerio.1.35295365.35295668 | 17 | 17 | |
| Danio_rerio.1.42241149.42241322 | Danio_rerio.1.42241149.42241322 | 30 | 30 | |
| Danio_rerio.1.44070566.44070840 | Danio_rerio.1.44070566.44070840 | 17 | 17 | |
| Danio_rerio.1.46410167.46410317 | Danio_rerio.1.46410167.46410317 | 33 | 33 | |
| Danio_rerio.1.46425056.46425217 | Danio_rerio.1.46425056.46425217 | 33 | 33 | |
| Danio_rerio.10.11294348.11294150 | Danio_rerio.10.11294348.11294150 | 18 | 18 | |
| Danio_rerio.10.11300795.11300598 | Danio_rerio.10.11300795.11300598 | 34 | 34 | |
| Danio_rerio.10.11775672.11775490 | Danio_rerio.10.11775672.11775490 | 34 | 34 | |
| Danio_rerio.10.1631139.1630985 | Danio_rerio.10.1631139.1630985 | 27 | 27 | |
| Danio_rerio.10.16979013.16979308 | Danio_rerio.10.16979013.16979308 | 34 | 34 | |
| Danio_rerio.10.25116020.25116178 | Danio_rerio.10.25116020.25116178 | 21 | 21 | |
| Danio_rerio.10.32015598.32014865 | Danio_rerio.10.32015598.32014865 | 22 | 22 | |
| Danio_rerio.10.33338435.33338270 | Danio_rerio.10.33338435.33338270 | 20 | 20 | |
| Danio_rerio.10.5548973.5548817 | Danio_rerio.10.5548973.5548817 | 11 | 11 | |

| | | | | |
|---|---|---|---|---|
| Danio_rerio.11.12311921.12312121 | Danio_rerio.11.12311921.12312121 | 31 | 31 | |
| Danio_rerio.11.16233723.16233931 | Danio_rerio.11.16233723.16233931 | 27 | 27 | |
| Danio_rerio.11.16256857.16257008 | Danio_rerio.11.16256857.16257008 | 35 | 35 | |
| Danio_rerio.11.18269387.18269231 | Danio_rerio.11.18269387.18269231 | 16 | 16 | |
| Danio_rerio.11.19924211.19924416 | Danio_rerio.11.19924211.19924416 | 39 | 39 | |
| Danio_rerio.11.23200485.23200305 | Danio_rerio.11.23200485.23200305 | 13 | 13 | |
| Danio_rerio.11.27397745.27398046 | Danio_rerio.11.27397745.27398046 | 25 | 25 | |
| Danio_rerio.11.28697579.28697189 | Danio_rerio.11.28697579.28697189 | 20 | 20 | |
| Danio_rerio.11.3201229.3201384 | Danio_rerio.11.3201229.3201384 | 25 | 25 | |
| Danio_rerio.11.39782316.39782167 | Danio_rerio.11.39782316.39782167 | 27 | 29 | multiple blast hit in proteome: Salanx_acuticepstmT02 |
| Danio_rerio.12.14776680.14776875 | Danio_rerio.12.14776680.14776875 | 14 | 14 | |
| Danio_rerio.12.14887893.14888055 | Danio_rerio.12.14887893.14888055 | 28 | 28 | |
| Danio_rerio.12.21063494.21063674 | Danio_rerio.12.21063494.21063674 | 24 | 24 | |
| Danio_rerio.12.23845778.23845268 | Danio_rerio.12.23845778.23845268 | 19 | 19 | |
| Danio_rerio.12.23917835.23918116 | Danio_rerio.12.23917835.23918116 | 19 | 20 | too short: Alepocephalus_bicolor |
| Danio_rerio.12.23920360.23920569 | Danio_rerio.12.23920360.23920569 | 27 | 27 | |
| Danio_rerio.12.23983879.23984119 | Danio_rerio.12.23983879.23984119 | 18 | 18 | |
| Danio_rerio.12.26434797.26434590 | Danio_rerio.12.26434797.26434590 | 26 | 26 | |
| Danio_rerio.12.28657090.28657269 | Danio_rerio.12.28657090.28657269 | 26 | 26 | |
| Danio_rerio.12.30353886.30354051 | Danio_rerio.12.30353886.30354051 | 15 | 15 | |
| Danio_rerio.12.31929346.31929176 | Danio_rerio.12.31929346.31929176 | 33 | 33 | |
| Danio_rerio.12.31958040.31958252 | Danio_rerio.12.31958040.31958252 | 41 | 41 | |

| | | | | |
|---|---|---|---|---|
| Danio_rerio.12.35305933.35305693 | Danio_rerio.12.35305933.35305693 | 25 | 25 | |
| Danio_rerio.12.35313681.35313889 | Danio_rerio.12.35313681.35313889 | 19 | 19 | |
| Danio_rerio.12.37171331.37171537 | Danio_rerio.12.37171331.37171537 | 15 | 15 | |
| Danio_rerio.12.40250032.40250256 | Danio_rerio.12.40250032.40250256 | 26 | 26 | |
| Danio_rerio.12.9956888.9957060 | Danio_rerio.12.9956888.9957060 | 12 | 12 | |
| Danio_rerio.12.9967973.9968136 | Danio_rerio.12.9967973.9968136 | 40 | 40 | |
| Danio_rerio.13.12685153.12685346 | Danio_rerio.13.12685153.12685346 | 25 | 25 | |
| Danio_rerio.13.12773697.12774412 | Danio_rerio.13.12773697.12774412 | 17 | 19 | too short: Limnothrissa_miodon,Salmo_truttatm20154 |
| Danio_rerio.13.12775856.12776101 | Danio_rerio.13.12775856.12776101 | 17 | 17 | |
| Danio_rerio.13.1277793.1277399 | Danio_rerio.13.1277793.1277399 | 14 | 14 | |
| Danio_rerio.13.12789906.12790132 | Danio_rerio.13.12789906.12790132 | 28 | 28 | |
| Danio_rerio.13.13858786.13858959 | Danio_rerio.13.13858786.13858959 | 23 | 23 | |
| Danio_rerio.13.18463225.18463413 | Danio_rerio.13.18463225.18463413 | 38 | 38 | |
| Danio_rerio.13.19367795.19368185 | Danio_rerio.13.19367795.19368185 | 35 | 35 | |
| Danio_rerio.13.24125183.24125443 | Danio_rerio.13.24125183.24125443 | 20 | 20 | |
| Danio_rerio.13.25809403.25809246 | Danio_rerio.13.25809403.25809246 | 34 | 34 | |
| Danio_rerio.13.25810415.25810236 | Danio_rerio.13.25810415.25810236 | 42 | 42 | |
| Danio_rerio.13.25816686.25816430 | Danio_rerio.13.25816686.25816430 | 36 | 36 | |

| | | | | |
|---|---|---|---|---|
| Danio_rerio.13.30258154.30256712 | Danio_rerio.13.30258154.30256712 | 6 | 19 | too short: Alepocephalus_bicolor,Ateleopus_japonicus133tm,Evermanella_balbotm67eve,Galaxias_fasciatusCL282,Gymnotus_spZSM32021,Hime_formosana137tm,Ijimaia_loppeiBPS2503,Lepidogalaxias_salamandroidesSAM,Limnothrissa_miodon,Mallotus_villosustm016,Retropinna_tasmanicaCL284a,Schilbe_mystustm551b,Synodus_saurustm67syneve |
| Danio_rerio.13.31123940.31124158 | Danio_rerio.13.31123940.31124158 | 23 | 23 | |
| Danio_rerio.13.3616977.3617172 | Danio_rerio.13.3616977.3617172 | 24 | 25 | too short: Pantodon_bucholzi382tm |
| Danio_rerio.13.36212936.36212775 | Danio_rerio.13.36212936.36212775 | 25 | 25 | |
| Danio_rerio.13.36219960.36219803 | Danio_rerio.13.36219960.36219803 | 26 | 26 | |
| Danio_rerio.13.36222647.36222495 | Danio_rerio.13.36222647.36222495 | 26 | 26 | |
| Danio_rerio.13.37194273.37194003 | Danio_rerio.13.37194273.37194003 | 20 | 20 | |
| Danio_rerio.13.4263019.4263174 | Danio_rerio.13.4263019.4263174 | 30 | 30 | |
| Danio_rerio.13.43664682.43664837 | Danio_rerio.13.43664682.43664837 | 26 | 26 | |
| Danio_rerio.13.43863454.43863615 | Danio_rerio.13.43863454.43863615 | 30 | 30 | |
| Danio_rerio.13.4576158.4576320 | Danio_rerio.13.4576158.4576320 | 30 | 30 | |
| Danio_rerio.13.4628267.4628467 | Danio_rerio.13.4628267.4628467 | 25 | 25 | |
| Danio_rerio.13.50080710.50080522 | Danio_rerio.13.50080710.50080522 | 18 | 18 | |
| Danio_rerio.13.6543000.6542806 | Danio_rerio.13.6543000.6542806 | 22 | 22 | |
| Danio_rerio.14.4155878.4155603 | Danio_rerio.14.4155878.4155603 | 26 | 26 | |
| Danio_rerio.14.45858593.45858439 | Danio_rerio.14.45858593.45858439 | 30 | 30 | |
| Danio_rerio.14.47709684.47709855 | Danio_rerio.14.47709684.47709855 | 37 | 37 | |
| Danio_rerio.14.49325852.49325677 | Danio_rerio.14.49325852.49325677 | 23 | 23 | |

| | | | | |
|---|---|---|---|---|
| Danio_rerio.14.9688807.9688616 | Danio_rerio.14.9688807.9688616 | 35 | 35 | |
| Danio_rerio.15.13563118.13564642 | Danio_rerio.15.13563118.13564642 | 23 | 23 | |
| Danio_rerio.15.1509765.1509586 | Danio_rerio.15.1509765.1509586 | 32 | 32 | |
| Danio_rerio.15.1542177.1542341 | Danio_rerio.15.1542177.1542341 | 30 | 30 | |
| Danio_rerio.15.1565400.1565632 | Danio_rerio.15.1565400.1565632 | 29 | 29 | |
| Danio_rerio.15.19335915.19336166 | Danio_rerio.15.19335915.19336166 | 25 | 25 | |
| Danio_rerio.15.23136899.23136641 | Danio_rerio.15.23136899.23136641 | 14 | 14 | |
| Danio_rerio.15.25309293.25309139 | Danio_rerio.15.25309293.25309139 | 21 | 21 | |
| Danio_rerio.15.38759667.38759830 | Danio_rerio.15.38759667.38759830 | 31 | 31 | |
| Danio_rerio.15.43915383.43915174 | Danio_rerio.15.43915383.43915174 | 20 | 20 | |
| Danio_rerio.15.44034117.44033966 | Danio_rerio.15.44034117.44033966 | 24 | 24 | |
| Danio_rerio.16.10486450.10486648 | Danio_rerio.16.10486450.10486648 | 16 | 16 | |
| Danio_rerio.16.14287185.14287389 | Danio_rerio.16.14287185.14287389 | 17 | 18 | too short: Engraulis_encrassicolustm57 |
| Danio_rerio.16.18352845.18353006 | Danio_rerio.16.18352845.18353006 | 37 | 37 | |
| Danio_rerio.16.19755043.19754891 | Danio_rerio.16.19755043.19754891 | 24 | 24 | |
| Danio_rerio.16.34146314.34146491 | Danio_rerio.16.34146314.34146491 | 20 | 20 | |
| Danio_rerio.16.38141401.38141200 | Danio_rerio.16.38141401.38141200 | 16 | 16 | |
| Danio_rerio.16.42715246.42715402 | Danio_rerio.16.42715246.42715402 | 21 | 21 | |
| Danio_rerio.16.44470277.44470092 | Danio_rerio.16.44470277.44470092 | 31 | 31 | |
| Danio_rerio.16.45281103.45280763 | Danio_rerio.16.45281103.45280763 | 26 | 26 | |
| Danio_rerio.16.55608807.55608626 | Danio_rerio.16.55608807.55608626 | 39 | 39 | |
| Danio_rerio.16.56182262.56181994 | Danio_rerio.16.56182262.56181994 | 39 | 39 | |
| Danio_rerio.16.56184365.56184108 | Danio_rerio.16.56184365.56184108 | 23 | 23 | |

| | | | | |
|---|---|---|---|---|
| Danio_rerio.16.6349642.6349466 | Danio_rerio.16.6349642.6349466 | 25 | 25 | |
| Danio_rerio.17.10554466.10554631 | Danio_rerio.17.10554466.10554631 | 33 | 33 | |
| Danio_rerio.17.177244.177091 | Danio_rerio.17.177244.177091 | 37 | 37 | |
| Danio_rerio.17.22402840.22402680 | Danio_rerio.17.22402840.22402680 | 35 | 35 | |
| Danio_rerio.17.22445169.22445362 | Danio_rerio.17.22445169.22445362 | 19 | 19 | |
| Danio_rerio.17.24439918.24440142 | Danio_rerio.17.24439918.24440142 | 20 | 20 | |
| Danio_rerio.17.25457338.25457123 | Danio_rerio.17.25457338.25457123 | 19 | 19 | |
| Danio_rerio.17.28158598.28158424 | Danio_rerio.17.28158598.28158424 | 28 | 28 | |
| Danio_rerio.17.28741125.28741299 | Danio_rerio.17.28741125.28741299 | 38 | 38 | |
| Danio_rerio.17.29364211.29364028 | Danio_rerio.17.29364211.29364028 | 20 | 21 | too short: Lovettia_sealii_CSIROtr |
| Danio_rerio.17.33505893.33506057 | Danio_rerio.17.33505893.33506057 | 37 | 37 | |
| Danio_rerio.17.35525279.35525115 | Danio_rerio.17.35525279.35525115 | 41 | 41 | |
| Danio_rerio.17.35538270.35538503 | Danio_rerio.17.35538270.35538503 | 14 | 14 | |
| Danio_rerio.17.44937719.44937164 | Danio_rerio.17.44937719.44937164 | 26 | 26 | |
| Danio_rerio.17.51607569.51607400 | Danio_rerio.17.51607569.51607400 | 24 | 24 | |
| Danio_rerio.17.51611735.51611568 | Danio_rerio.17.51611735.51611568 | 28 | 28 | |
| Danio_rerio.17.7887549.7887391 | Danio_rerio.17.7887549.7887391 | 10 | 10 | |
| Danio_rerio.18.11695526.11695369 | Danio_rerio.18.11695526.11695369 | 10 | 10 | |
| Danio_rerio.18.11703004.11702767 | Danio_rerio.18.11703004.11702767 | 22 | 22 | |
| Danio_rerio.18.11714356.11714185 | Danio_rerio.18.11714356.11714185 | 22 | 22 | |
| Danio_rerio.18.14950656.14950443 | Danio_rerio.18.14950656.14950443 | 32 | 32 | |
| Danio_rerio.18.14987901.14987739 | Danio_rerio.18.14987901.14987739 | 19 | 19 | |
| Danio_rerio.18.15545791.15545994 | Danio_rerio.18.15545791.15545994 | 35 | 35 | |

| | | | | |
|---|---|---|---|---|
| Danio_rerio.18.15551355.15551549 | Danio_rerio.18.15551355.15551549 | 19 | 20 | multiple blast hit in proteome: Pantodon_bucholzi382tm |
| Danio_rerio.18.16761340.16761155 | Danio_rerio.18.16761340.16761155 | 18 | 18 | |
| Danio_rerio.18.18784958.18784737 | Danio_rerio.18.18784958.18784737 | 33 | 33 | |
| Danio_rerio.18.20275163.20275387 | Danio_rerio.18.20275163.20275387 | 33 | 33 | |
| Danio_rerio.18.20297779.20297582 | Danio_rerio.18.20297779.20297582 | 5 | 26 | multiple blast hit in proteome: Alepocephalus_bicolor,Alepocephalus_brevipinnitm66merge,Argentina_sphyraena029tm,Borostomias_antarcticustm762,Chanos_chanostm1777,Cromeria_occidentalistm583a,Danio_rerioD15,Distichodus_brevipinnitm559,Holtbyrnia_anomalatm117,Lepidogalaxias_salamandroidesSAM,Limnothrissa_miodon,Mallotus_villosustm016,Pantodon_bucholzi382tm,Pecoglossus_altivelistm211,Retropinna_tasmanicaCL284a,Retropinna_tasmanica_CSIROtr,Rutilus_rutilus2,Rutilus_rutilustm70,Schilbe_mystustm551b,Umbra_krameritm62,bait_sequences |
| Danio_rerio.18.20898144.20898343 | Danio_rerio.18.20898144.20898343 | 12 | 12 | |
| Danio_rerio.18.33525911.33526089 | Danio_rerio.18.33525911.33526089 | 32 | 32 | |
| Danio_rerio.18.36679109.36678938 | Danio_rerio.18.36679109.36678938 | 23 | 23 | |
| Danio_rerio.18.6138540.6138356 | Danio_rerio.18.6138540.6138356 | 11 | 11 | |
| Danio_rerio.18.7870276.7870422 | Danio_rerio.18.7870276.7870422 | 36 | 36 | |
| Danio_rerio.18.7973745.7973508 | Danio_rerio.18.7973745.7973508 | 16 | 16 | |
| Danio_rerio.19.27396673.27396461 | Danio_rerio.19.27396673.27396461 | 21 | 21 | |
| Danio_rerio.19.32132282.32132457 | Danio_rerio.19.32132282.32132457 | 19 | 19 | |

| | | | | |
|---|---|---|---|---|
| Danio_rerio.19.32777735.32778436 | Danio_rerio.19.32777735.32778436 | 1 | 23 | multiple blast hit in proteome: Alepocephalus_bicolor,Argentina_sphyraena029tm,Bathylagus_euryopstm112,Borostomias_antarcticustm762,Chanos_chanostm1777,Denticeps_clupeoidestm138,Elops_senegalensistm175,Evermanella_balbotm67eve,Galaxias_fasciatusCL282,Gymnotus_spZSM32021,Hime_formosana137tm,Lestrolepis_japonicustmT03,Lepidogalaxias_salamandroidesSAM,Limnothrissa_miodon,Lovettia_sealii_CSIROtr,Mallotus_villosustm016,Nansenia_oblitaBPS1997,Pantodon_bucholzi382tm,Retropinna_tasmanicaCL284a,Retropinna_tasmanica_CSIROtr,Synodus_saurustm67syneve,bait_sequences |
| Danio_rerio.19.3530431.3530277 | Danio_rerio.19.3530431.3530277 | 33 | 33 | |
| Danio_rerio.19.36689372.36689082 | Danio_rerio.19.36689372.36689082 | 16 | 16 | |
| Danio_rerio.19.38687277.38687552 | Danio_rerio.19.38687277.38687552 | 27 | 27 | |
| Danio_rerio.19.41860989.41860834 | Danio_rerio.19.41860989.41860834 | 11 | 11 | |
| Danio_rerio.19.8398009.8398331 | Danio_rerio.19.8398009.8398331 | 16 | 16 | |
| Danio_rerio.2.10692384.10692036 | Danio_rerio.2.10692384.10692036 | 19 | 19 | |
| Danio_rerio.2.31408501.31408327 | Danio_rerio.2.31408501.31408327 | 20 | 20 | |
| Danio_rerio.2.35317604.35317817 | Danio_rerio.2.35317604.35317817 | 13 | 13 | |
| Danio_rerio.2.36373184.36373421 | Danio_rerio.2.36373184.36373421 | 24 | 24 | |
| Danio_rerio.2.36957008.36957687 | Danio_rerio.2.36957008.36957687 | 22 | 22 | |
| Danio_rerio.2.4329008.4329234 | Danio_rerio.2.4329008.4329234 | 17 | 17 | |
| Danio_rerio.2.44686510.44686671 | Danio_rerio.2.44686510.44686671 | 26 | 26 | |
| Danio_rerio.2.44836306.44836131 | Danio_rerio.2.44836306.44836131 | 25 | 25 | |

| | | | | |
|---|---|---|---|---|
| Danio_rerio.2.45037351.45037511 | Danio_rerio.2.45037351.45037511 | 19 | 19 | |
| Danio_rerio.20.19519698.19519495 | Danio_rerio.20.19519698.19519495 | 19 | 19 | |
| Danio_rerio.20.23866727.23866572 | Danio_rerio.20.23866727.23866572 | 31 | 31 | |
| Danio_rerio.20.24314527.24314765 | Danio_rerio.20.24314527.24314765 | 16 | 16 | |
| Danio_rerio.20.25743795.25743985 | Danio_rerio.20.25743795.25743985 | 15 | 15 | |
| Danio_rerio.20.28311208.28311047 | Danio_rerio.20.28311208.28311047 | 21 | 21 | |
| Danio_rerio.20.30961284.30961102 | Danio_rerio.20.30961284.30961102 | 36 | 36 | |
| Danio_rerio.20.32523380.32523545 | Danio_rerio.20.32523380.32523545 | 42 | 42 | |
| Danio_rerio.20.34205093.34204937 | Danio_rerio.20.34205093.34204937 | 25 | 25 | |
| Danio_rerio.20.39386770.39386588 | Danio_rerio.20.39386770.39386588 | 25 | 25 | |
| Danio_rerio.20.4037479.4035425 | Danio_rerio.20.4037479.4035425 | 21 | 21 | |
| Danio_rerio.20.43707424.43707666 | Danio_rerio.20.43707424.43707666 | 36 | 36 | |
| Danio_rerio.20.49394052.49394235 | Danio_rerio.20.49394052.49394235 | 17 | 17 | |
| Danio_rerio.20.49399914.49400153 | Danio_rerio.20.49399914.49400153 | 22 | 22 | |
| Danio_rerio.20.5473684.5473845 | Danio_rerio.20.5473684.5473845 | 27 | 27 | |
| Danio_rerio.21.11889280.11889116 | Danio_rerio.21.11889280.11889116 | 20 | 20 | |
| Danio_rerio.21.13482741.13482578 | Danio_rerio.21.13482741.13482578 | 23 | 23 | |
| Danio_rerio.21.21817705.21817895 | Danio_rerio.21.21817705.21817895 | 26 | 26 | |
| Danio_rerio.21.21977503.21977310 | Danio_rerio.21.21977503.21977310 | 24 | 24 | |
| Danio_rerio.21.29152691.29152541 | Danio_rerio.21.29152691.29152541 | 17 | 17 | |
| Danio_rerio.21.33792499.33792678 | Danio_rerio.21.33792499.33792678 | 28 | 28 | |
| Danio_rerio.21.3478758.3478937 | Danio_rerio.21.3478758.3478937 | 25 | 25 | |
| Danio_rerio.21.39222598.39222411 | Danio_rerio.21.39222598.39222411 | 17 | 17 | |

| | | | | |
|---|---|---|---|---|
| Danio_rerio.21.40517808.40517649 | Danio_rerio.21.40517808.40517649 | 38 | 38 | |
| Danio_rerio.21.9124967.9124663 | Danio_rerio.21.9124967.9124663 | 41 | 41 | |
| Danio_rerio.21.9251039.9251232 | Danio_rerio.21.9251039.9251232 | 26 | 28 | multiple blast hit in proteome: Danio_rerioD15,Galaxias_fasciatusCL282 |
| Danio_rerio.21.935969.936261 | Danio_rerio.21.935969.936261 | 23 | 23 | |
| Danio_rerio.22.11182114.11181932 | Danio_rerio.22.11182114.11181932 | 29 | 29 | |
| Danio_rerio.22.40625225.40625433 | Danio_rerio.22.40625225.40625433 | 32 | 32 | |
| Danio_rerio.23.20480167.20479926 | Danio_rerio.23.20480167.20479926 | 21 | 21 | |
| Danio_rerio.23.21625189.21625352 | Danio_rerio.23.21625189.21625352 | 25 | 25 | |
| Danio_rerio.23.28770180.28770403 | Danio_rerio.23.28770180.28770403 | 22 | 22 | |
| Danio_rerio.23.28779772.28780125 | Danio_rerio.23.28779772.28780125 | 26 | 26 | |
| Danio_rerio.23.29224430.29224258 | Danio_rerio.23.29224430.29224258 | 33 | 33 | |
| Danio_rerio.23.29246807.29246237 | Danio_rerio.23.29246807.29246237 | 25 | 25 | |
| Danio_rerio.23.29755050.29754876 | Danio_rerio.23.29755050.29754876 | 40 | 40 | |
| Danio_rerio.23.31257191.31257371 | Danio_rerio.23.31257191.31257371 | 39 | 39 | |
| Danio_rerio.23.35444982.35444824 | Danio_rerio.23.35444982.35444824 | 23 | 23 | |
| Danio_rerio.23.512955.512771 | Danio_rerio.23.512955.512771 | 21 | 21 | |
| Danio_rerio.23.9188003.9188283 | Danio_rerio.23.9188003.9188283 | 28 | 28 | |
| Danio_rerio.24.21270375.21270538 | Danio_rerio.24.21270375.21270538 | 19 | 19 | |
| Danio_rerio.24.22337089.22337324 | Danio_rerio.24.22337089.22337324 | 16 | 16 | |
| Danio_rerio.24.24325336.24325570 | Danio_rerio.24.24325336.24325570 | 39 | 39 | |
| Danio_rerio.24.25628043.25627842 | Danio_rerio.24.25628043.25627842 | 14 | 14 | |
| Danio_rerio.24.36473686.36473856 | Danio_rerio.24.36473686.36473856 | 31 | 31 | |

| | | | | |
|---|---|---|---|---|
| Danio_rerio.24.37448217.37448483 | Danio_rerio.24.37448217.37448483 | 20 | 20 | |
| Danio_rerio.24.37481832.37482113 | Danio_rerio.24.37481832.37482113 | 14 | 14 | |
| Danio_rerio.25.17689976.17690216 | Danio_rerio.25.17689976.17690216 | 38 | 38 | |
| Danio_rerio.25.18442403.18442239 | Danio_rerio.25.18442403.18442239 | 31 | 31 | |
| Danio_rerio.25.18497602.18497790 | Danio_rerio.25.18497602.18497790 | 22 | 22 | |
| Danio_rerio.25.29603844.29603690 | Danio_rerio.25.29603844.29603690 | 13 | 13 | |
| Danio_rerio.25.29606624.29606456 | Danio_rerio.25.29606624.29606456 | 33 | 33 | |
| Danio_rerio.25.8313523.8313329 | Danio_rerio.25.8313523.8313329 | 28 | 28 | |
| Danio_rerio.3.16020366.16020173 | Danio_rerio.3.16020366.16020173 | 20 | 20 | |
| Danio_rerio.3.18741377.18742142 | Danio_rerio.3.18741377.18742142 | 33 | 33 | |
| Danio_rerio.3.25182538.25182125 | Danio_rerio.3.25182538.25182125 | 21 | 29 | multiple blast hit in proteome: Argentina_sphyraena029tm |
| Danio_rerio.3.28994856.28995066 | Danio_rerio.3.28994856.28995066 | 21 | 21 | |
| Danio_rerio.3.31281872.31281654 | Danio_rerio.3.31281872.31281654 | 34 | 34 | |
| Danio_rerio.3.32021560.32021284 | Danio_rerio.3.32021560.32021284 | 44 | 44 | |
| Danio_rerio.3.34007978.34007774 | Danio_rerio.3.34007978.34007774 | 31 | 31 | |
| Danio_rerio.3.61690400.61690212 | Danio_rerio.3.61690400.61690212 | 18 | 18 | |
| Danio_rerio.4.11489621.11489392 | Danio_rerio.4.11489621.11489392 | 28 | 28 | |
| Danio_rerio.4.14158946.14158759 | Danio_rerio.4.14158946.14158759 | 24 | 24 | |
| Danio_rerio.4.14678784.14678978 | Danio_rerio.4.14678784.14678978 | 24 | 24 | |
| Danio_rerio.4.14695312.14695549 | Danio_rerio.4.14695312.14695549 | 23 | 26 | too short: Dussumiera_elopsoides037tm,Rutilus_rutilus2,Schilbe_mystustm551b |
| Danio_rerio.4.1520960.1520751 | Danio_rerio.4.1520960.1520751 | 21 | 21 | |
| Danio_rerio.4.1633065.1632884 | Danio_rerio.4.1633065.1632884 | 22 | 22 | |

| | | | | |
|---|---|---|---|---|
| Danio_rerio.4.18428325.18428137 | Danio_rerio.4.18428325.18428137 | 41 | 41 | |
| Danio_rerio.4.21370322.21370143 | Danio_rerio.4.21370322.21370143 | 30 | 30 | |
| Danio_rerio.4.2442747.2442586 | Danio_rerio.4.2442747.2442586 | 11 | 11 | |
| Danio_rerio.4.9953286.9953607 | Danio_rerio.4.9953286.9953607 | 20 | 20 | |
| Danio_rerio.5.17904386.17904553 | Danio_rerio.5.17904386.17904553 | 33 | 33 | |
| Danio_rerio.5.17933857.17933701 | Danio_rerio.5.17933857.17933701 | 34 | 34 | |
| Danio_rerio.5.20758624.20758424 | Danio_rerio.5.20758624.20758424 | 18 | 18 | |
| Danio_rerio.5.24335393.24335203 | Danio_rerio.5.24335393.24335203 | 25 | 25 | |
| Danio_rerio.5.26211472.26210808 | Danio_rerio.5.26211472.26210808 | 19 | 20 | multiple blast hit in proteome: Galaxias_fasciatusCL282 |
| Danio_rerio.5.26790347.26790172 | Danio_rerio.5.26790347.26790172 | 25 | 25 | |
| Danio_rerio.5.27182114.27181911 | Danio_rerio.5.27182114.27181911 | 29 | 29 | |
| Danio_rerio.5.27449614.27449796 | Danio_rerio.5.27449614.27449796 | 10 | 10 | |
| Danio_rerio.5.32228605.32228818 | Danio_rerio.5.32228605.32228818 | 28 | 28 | |
| Danio_rerio.5.32247404.32247571 | Danio_rerio.5.32247404.32247571 | 21 | 21 | |
| Danio_rerio.5.33988949.33989189 | Danio_rerio.5.33988949.33989189 | 14 | 14 | |
| Danio_rerio.5.35254252.35254827 | Danio_rerio.5.35254252.35254827 | 15 | 15 | |
| Danio_rerio.5.38170455.38170229 | Danio_rerio.5.38170455.38170229 | 27 | 27 | |
| Danio_rerio.5.43258539.43258378 | Danio_rerio.5.43258539.43258378 | 40 | 40 | |
| Danio_rerio.5.43261080.43260889 | Danio_rerio.5.43261080.43260889 | 18 | 18 | |
| Danio_rerio.5.50442923.50442538 | Danio_rerio.5.50442923.50442538 | 17 | 17 | |
| Danio_rerio.5.66051445.66051641 | Danio_rerio.5.66051445.66051641 | 11 | 11 | |
| Danio_rerio.5.71821170.71820952 | Danio_rerio.5.71821170.71820952 | 29 | 29 | |

| | | | | |
|---|---|---|---|---|
| Danio_rerio.6.14888366.14888548 | Danio_rerio.6.14888366.14888548 | 16 | 16 | |
| Danio_rerio.6.2264949.2265119 | Danio_rerio.6.2264949.2265119 | 30 | 30 | |
| Danio_rerio.6.2333526.2333690 | Danio_rerio.6.2333526.2333690 | 28 | 28 | |
| Danio_rerio.6.28987678.28987471 | Danio_rerio.6.28987678.28987471 | 19 | 19 | |
| Danio_rerio.6.29101227.29101401 | Danio_rerio.6.29101227.29101401 | 41 | 41 | |
| Danio_rerio.6.29856354.29856205 | Danio_rerio.6.29856354.29856205 | 25 | 25 | |
| Danio_rerio.6.30360290.30360853 | Danio_rerio.6.30360290.30360853 | 20 | 25 | too short: Bathylagus_euryopstm112,Gonorhynchus_abbreviatus098tm,Holtbyrnia_anomalatm117,Lestrolepis_japonicustmT03,Limnothrissa_miodon |
| Danio_rerio.6.3295020.3295183 | Danio_rerio.6.3295020.3295183 | 41 | 41 | |
| Danio_rerio.6.36814228.36814430 | Danio_rerio.6.36814228.36814430 | 17 | 17 | |
| Danio_rerio.6.37734602.37734780 | Danio_rerio.6.37734602.37734780 | 25 | 25 | |
| Danio_rerio.6.37740390.37740604 | Danio_rerio.6.37740390.37740604 | 30 | 30 | |
| Danio_rerio.6.40759925.40759718 | Danio_rerio.6.40759925.40759718 | 16 | 16 | |
| Danio_rerio.6.50351368.50351533 | Danio_rerio.6.50351368.50351533 | 30 | 30 | |
| Danio_rerio.6.51516474.51516288 | Danio_rerio.6.51516474.51516288 | 15 | 15 | |
| Danio_rerio.6.57415342.57415169 | Danio_rerio.6.57415342.57415169 | 18 | 18 | |
| Danio_rerio.6.6198164.6197912 | Danio_rerio.6.6198164.6197912 | 30 | 31 | too short: Gonorhynchus_abbreviatus098tm |
| Danio_rerio.7.10385196.10384961 | Danio_rerio.7.10385196.10384961 | 14 | 14 | |
| Danio_rerio.7.12988024.12987840 | Danio_rerio.7.12988024.12987840 | 30 | 30 | |
| Danio_rerio.7.14523737.14523507 | Danio_rerio.7.14523737.14523507 | 34 | 34 | |
| Danio_rerio.7.21633384.21633548 | Danio_rerio.7.21633384.21633548 | 20 | 20 | |
| Danio_rerio.7.22597903.22598059 | Danio_rerio.7.22597903.22598059 | 15 | 15 | |

| | | | | |
|---|---|---|---|---|
| Danio_rerio.7.33959579.33959351 | Danio_rerio.7.33959579.33959351 | 35 | 35 | |
| Danio_rerio.7.51857227.51857443 | Danio_rerio.7.51857227.51857443 | 24 | 24 | |
| Danio_rerio.7.51861227.51861496 | Danio_rerio.7.51861227.51861496 | 39 | 39 | |
| Danio_rerio.7.54383033.54383225 | Danio_rerio.7.54383033.54383225 | 16 | 16 | |
| Danio_rerio.7.54387879.54388084 | Danio_rerio.7.54387879.54388084 | 24 | 24 | |
| Danio_rerio.7.62631771.62631617 | Danio_rerio.7.62631771.62631617 | 47 | 47 | |
| Danio_rerio.7.67298495.67298751 | Danio_rerio.7.67298495.67298751 | 35 | 35 | |
| Danio_rerio.7.69813677.69813832 | Danio_rerio.7.69813677.69813832 | 26 | 26 | |
| Danio_rerio.8.10280911.10281123 | Danio_rerio.8.10280911.10281123 | 31 | 33 | too short: Lovettia_sealii_CSIROtr,Trachypterus_trachipterustm119 |
| Danio_rerio.8.12264492.12264303 | Danio_rerio.8.12264492.12264303 | 41 | 41 | |
| Danio_rerio.8.21391447.21391131 | Danio_rerio.8.21391447.21391131 | 24 | 24 | |
| Danio_rerio.8.31880701.31880541 | Danio_rerio.8.31880701.31880541 | 24 | 24 | |
| Danio_rerio.8.37676522.37676718 | Danio_rerio.8.37676522.37676718 | 25 | 25 | |
| Danio_rerio.8.41829083.41828907 | Danio_rerio.8.41829083.41828907 | 21 | 21 | |
| Danio_rerio.8.54109062.54108899 | Danio_rerio.8.54109062.54108899 | 31 | 31 | |
| Danio_rerio.8.54357458.54357287 | Danio_rerio.8.54357458.54357287 | 12 | 12 | |
| Danio_rerio.8.9029197.9028817 | Danio_rerio.8.9029197.9028817 | 34 | 34 | |
| Danio_rerio.9.16436007.16436207 | Danio_rerio.9.16436007.16436207 | 26 | 26 | |
| Danio_rerio.9.17569522.17569313 | Danio_rerio.9.17569522.17569313 | 28 | 28 | |
| Danio_rerio.9.17643391.17643219 | Danio_rerio.9.17643391.17643219 | 22 | 22 | |
| Danio_rerio.9.17668575.17668301 | Danio_rerio.9.17668575.17668301 | 38 | 38 | |
| Danio_rerio.9.21889423.21889596 | Danio_rerio.9.21889423.21889596 | 21 | 21 | |

| | | | | |
|---|---|---|---|---|
| Danio_rerio.9.22250638.22250815 | Danio_rerio.9.22250638.22250815 | 35 | 35 | |
| Danio_rerio.9.22380463.22380632 | Danio_rerio.9.22380463.22380632 | 34 | 34 | |
| Danio_rerio.9.28750682.28750235 | Danio_rerio.9.28750682.28750235 | 30 | 30 | |
| Danio_rerio.9.32772715.32772533 | Danio_rerio.9.32772715.32772533 | 33 | 33 | |
| Danio_rerio.9.33208571.33208396 | Danio_rerio.9.33208571.33208396 | 22 | 22 | |
| Danio_rerio.9.33675775.33675616 | Danio_rerio.9.33675775.33675616 | 26 | 26 | |
| Danio_rerio.9.3384683.3384995 | Danio_rerio.9.3384683.3384995 | 38 | 38 | |
| Danio_rerio.9.35991142.35991369 | Danio_rerio.9.35991142.35991369 | 40 | 40 | |
| Danio_rerio.9.39452552.39452871 | Danio_rerio.9.39452552.39452871 | 25 | 25 | |
| Danio_rerio.9.56443793.56443642 | Danio_rerio.9.56443793.56443642 | 15 | 15 | |
| Danio_rerio.9.5939584.5939378 | Danio_rerio.9.5939584.5939378 | 39 | 39 | |
| Danio_rerio.9.6994508.6994717 | Danio_rerio.9.6994508.6994717 | 12 | 12 | |
| Danio_rerio.Zv9_NA985.956.752 | Danio_rerio.Zv9_NA985.956.752 | 13 | 13 | |
| Gasterosteus_aculeatus.groupI.12206723.12206886 | Gasterosteus_aculeatus.groupI.12206723.12206886 | 20 | 20 | |
| Gasterosteus_aculeatus.groupI.15844286.15844486 | Gasterosteus_aculeatus.groupI.15844286.15844486 | 35 | 35 | |
| Gasterosteus_aculeatus.groupI.15866514.15866678 | Gasterosteus_aculeatus.groupI.15866514.15866678 | 41 | 41 | |
| Gasterosteus_aculeatus.groupI.22775202.22775470 | Gasterosteus_aculeatus.groupI.22775202.22775470 | 19 | 19 | |
| Gasterosteus_aculeatus.groupI.5695811.5695565 | Gasterosteus_aculeatus.groupI.5695811.5695565 | 22 | 23 | multiple blast hit in proteome: Schilbe_mystustm551b |
| Gasterosteus_aculeatus.groupI.6463966.6464161 | Gasterosteus_aculeatus.groupI.6463966.6464161 | 22 | 22 | |
| Gasterosteus_aculeatus.groupI.7540320.7540507 | Gasterosteus_aculeatus.groupI.7540320.7540507 | 35 | 35 | |
| Gasterosteus_aculeatus.groupI.7990526.7990700 | Gasterosteus_aculeatus.groupI.7990526.7990700 | 14 | 14 | |

| | | | | |
|---|---|---|---|---|
| Gasterosteus_aculeatus.groupI.9579603.9579921 | Gasterosteus_aculeatus.groupI.9579603.9579921 | 10 | 10 | |
| Gasterosteus_aculeatus.groupII.10483463.10482668 | Gasterosteus_aculeatus.groupII.10483463.10482668 | 23 | 23 | |
| Gasterosteus_aculeatus.groupII.11497785.11498042 | Gasterosteus_aculeatus.groupII.11497785.11498042 | 31 | 31 | |
| Gasterosteus_aculeatus.groupII.19138798.19138960 | Gasterosteus_aculeatus.groupII.19138798.19138960 | 13 | 13 | |
| Gasterosteus_aculeatus.groupII.22647373.22646549 | Gasterosteus_aculeatus.groupII.22647373.22646549 | 25 | 25 | |
| Gasterosteus_aculeatus.groupII.316006.316200 | Gasterosteus_aculeatus.groupII.316006.316200 | 21 | 21 | |
| Gasterosteus_aculeatus.groupII.5730044.5730259 | Gasterosteus_aculeatus.groupII.5730044.5730259 | 22 | 22 | |
| Gasterosteus_aculeatus.groupII.5733532.5733724 | Gasterosteus_aculeatus.groupII.5733532.5733724 | 38 | 38 | |
| Gasterosteus_aculeatus.groupII.7726048.7726212 | Gasterosteus_aculeatus.groupII.7726048.7726212 | 40 | 40 | |
| Gasterosteus_aculeatus.groupII.8576776.8576990 | Gasterosteus_aculeatus.groupII.8576776.8576990 | 29 | 29 | |
| Gasterosteus_aculeatus.groupII.8662069.8661797 | Gasterosteus_aculeatus.groupII.8662069.8661797 | 16 | 16 | |
| Gasterosteus_aculeatus.groupIII.14087422.14087147 | Gasterosteus_aculeatus.groupIII.14087422.14087147 | 29 | 29 | |
| Gasterosteus_aculeatus.groupIII.7534838.7535419 | Gasterosteus_aculeatus.groupIII.7534838.7535419 | 40 | 40 | |
| Gasterosteus_aculeatus.groupIV.10641242.10641074 | Gasterosteus_aculeatus.groupIV.10641242.10641074 | 14 | 14 | |

| | | | | |
|---|---|---|---|---|
| Gasterosteus_aculeatus.groupIV.10934749.10934508 | Gasterosteus_aculeatus.groupIV.10934749.10934508 | 19 | 33 | multiple blast hit in proteome: Alestes_baremozetm285,Arctozensus_risso,Ateleopus_japonicus133tm,Borostomias_antarcticustm762,Gonorhynchus_abbreviatus098tm,Gymnotus_spZSM32021,Ijimaia_loppeiBPS2503,Lestrolepis_japonicustmT03,Lovettia_sealii_CSIROtr,Nansenia_oblitaBPS1997,Salmo_truttatm20154,Synodus_saurustm67syneve,Trachypterus_trachipterustm119,Umbra_krameritm62 |
| Gasterosteus_aculeatus.groupIV.13404607.13404785 | Gasterosteus_aculeatus.groupIV.13404607.13404785 | 28 | 28 | |
| Gasterosteus_aculeatus.groupIV.14516687.14515926 | Gasterosteus_aculeatus.groupIV.14516687.14515926 | 41 | 41 | |
| Gasterosteus_aculeatus.groupIV.16567564.16567741 | Gasterosteus_aculeatus.groupIV.16567564.16567741 | 21 | 21 | |
| Gasterosteus_aculeatus.groupIV.26218502.26218667 | Gasterosteus_aculeatus.groupIV.26218502.26218667 | 11 | 11 | |
| Gasterosteus_aculeatus.groupIV.30968001.30968220 | Gasterosteus_aculeatus.groupIV.30968001.30968220 | 18 | 18 | |
| Gasterosteus_aculeatus.groupIV.3670978.3671531 | Gasterosteus_aculeatus.groupIV.3670978.3671531 | 35 | 35 | |
| Gasterosteus_aculeatus.groupIV.3671571.3672899 | Gasterosteus_aculeatus.groupIV.3671571.3672899 | 27 | 27 | |
| Gasterosteus_aculeatus.groupIV.3817767.3817926 | Gasterosteus_aculeatus.groupIV.3817767.3817926 | 32 | 32 | |
| Gasterosteus_aculeatus.groupIV.7532486.7532130 | Gasterosteus_aculeatus.groupIV.7532486.7532130 | 22 | 22 | |
| Gasterosteus_aculeatus.groupIX.10730774.10730459 | Gasterosteus_aculeatus.groupIX.10730774.10730459 | 24 | 24 | |
| Gasterosteus_aculeatus.groupIX.11195301.11195125 | Gasterosteus_aculeatus.groupIX.11195301.11195125 | 34 | 34 | |
| Gasterosteus_aculeatus.groupIX.11196191.11196036 | Gasterosteus_aculeatus.groupIX.11196191.11196036 | 29 | 29 | |
| Gasterosteus_aculeatus.groupIX.12980024.12979858 | Gasterosteus_aculeatus.groupIX.12980024.12979858 | 39 | 39 | |

| | | | | |
|---|---|---|---|---|
| Gasterosteus_aculeatus.groupIX.13986085.13985872 | Gasterosteus_aculeatus.groupIX.13986085.13985872 | 36 | 36 | |
| Gasterosteus_aculeatus.groupIX.19232781.19232452 | Gasterosteus_aculeatus.groupIX.19232781.19232452 | 32 | 32 | |
| Gasterosteus_aculeatus.groupIX.19237881.19237709 | Gasterosteus_aculeatus.groupIX.19237881.19237709 | 26 | 26 | |
| Gasterosteus_aculeatus.groupIX.19246025.19245863 | Gasterosteus_aculeatus.groupIX.19246025.19245863 | 18 | 18 | |
| Gasterosteus_aculeatus.groupIX.19248905.19248691 | Gasterosteus_aculeatus.groupIX.19248905.19248691 | 35 | 35 | |
| Gasterosteus_aculeatus.groupIX.19278025.19277762 | Gasterosteus_aculeatus.groupIX.19278025.19277762 | 17 | 17 | |
| Gasterosteus_aculeatus.groupIX.2311910.2311755 | Gasterosteus_aculeatus.groupIX.2311910.2311755 | 18 | 18 | |
| Gasterosteus_aculeatus.groupIX.457212.457389 | Gasterosteus_aculeatus.groupIX.457212.457389 | 10 | 10 | |
| Gasterosteus_aculeatus.groupIX.464830.464997 | Gasterosteus_aculeatus.groupIX.464830.464997 | 23 | 23 | |
| Gasterosteus_aculeatus.groupIX.474652.474493 | Gasterosteus_aculeatus.groupIX.474652.474493 | 41 | 41 | |
| Gasterosteus_aculeatus.groupIX.5894096.5896405 | Gasterosteus_aculeatus.groupIX.5894096.5896405 | 41 | 41 | |
| Gasterosteus_aculeatus.groupIX.5960342.5960162 | Gasterosteus_aculeatus.groupIX.5960342.5960162 | 22 | 22 | |
| Gasterosteus_aculeatus.groupIX.6994313.6994140 | Gasterosteus_aculeatus.groupIX.6994313.6994140 | 14 | 14 | |
| Gasterosteus_aculeatus.groupIX.7010124.7009852 | Gasterosteus_aculeatus.groupIX.7010124.7009852 | 27 | 27 | |
| Gasterosteus_aculeatus.groupIX.8227145.8226941 | Gasterosteus_aculeatus.groupIX.8227145.8226941 | 30 | 30 | |
| Gasterosteus_aculeatus.groupV.12103303.12103491 | Gasterosteus_aculeatus.groupV.12103303.12103491 | 21 | 21 | |
| Gasterosteus_aculeatus.groupV.121793.122059 | Gasterosteus_aculeatus.groupV.121793.122059 | 33 | 33 | |
| Gasterosteus_aculeatus.groupV.12234447.12234222 | Gasterosteus_aculeatus.groupV.12234447.12234222 | 21 | 21 | |

| | | | | |
|---|---|---|---|---|
| Gasterosteus_aculeatus.groupV.3673287.3672838 | Gasterosteus_aculeatus.groupV.3673287.3672838 | 22 | 22 | |
| Gasterosteus_aculeatus.groupV.4690797.4691046 | Gasterosteus_aculeatus.groupV.4690797.4691046 | 24 | 24 | |
| Gasterosteus_aculeatus.groupV.6334923.6335076 | Gasterosteus_aculeatus.groupV.6334923.6335076 | 24 | 24 | |
| Gasterosteus_aculeatus.groupVI.10948740.10948904 | Gasterosteus_aculeatus.groupVI.10948740.10948904 | 26 | 26 | |
| Gasterosteus_aculeatus.groupVI.15678593.15678429 | Gasterosteus_aculeatus.groupVI.15678593.15678429 | 22 | 23 | multiple blast hit in proteome: Rutilus_rutilustm70 |
| Gasterosteus_aculeatus.groupVI.2442413.2443057 | Gasterosteus_aculeatus.groupVI.2442413.2443057 | 24 | 24 | |
| Gasterosteus_aculeatus.groupVI.2544394.2544560 | Gasterosteus_aculeatus.groupVI.2544394.2544560 | 12 | 30 | multiple blast hit in proteome: Arctozensus_risso,Argentina_sphyraena029tm,Astronesthes_luciferT04tm,Ateleopus_japonicus133tm,Bathylagus_euryopstm112,Borostomias_antarcticustm762,Coregonus_spZSM13380,Evermanella_balbotm67eve,Galaxias_fasciatusCL282,Gonorhynchus_abbreviatus098tm,Ijimaia_loppeiBPS2503,Lepidogalaxias_salamandroidesSAM,Lovettia_sealii_CSIROtr,Mallotus_villosustm016,Osmerus_eperlanustm785,Retropinna_tasmanicaCL284a,Salanx_acuticepstmT02,Synodus_saurustm67syneve |
| Gasterosteus_aculeatus.groupVI.3173429.3173258 | Gasterosteus_aculeatus.groupVI.3173429.3173258 | 36 | 36 | |
| Gasterosteus_aculeatus.groupVI.3372181.3372369 | Gasterosteus_aculeatus.groupVI.3372181.3372369 | 23 | 23 | |
| Gasterosteus_aculeatus.groupVI.3395485.3395674 | Gasterosteus_aculeatus.groupVI.3395485.3395674 | 28 | 28 | |
| Gasterosteus_aculeatus.groupVI.3396192.3396411 | Gasterosteus_aculeatus.groupVI.3396192.3396411 | 24 | 24 | |
| Gasterosteus_aculeatus.groupVI.6302987.6302825 | Gasterosteus_aculeatus.groupVI.6302987.6302825 | 24 | 24 | |

| | | | | |
|---|---|---|---|---|
| Gasterosteus_aculeatus.groupVI.6998907.6998287 | Gasterosteus_aculeatus.groupVI.6998907.6998287 | 19 | 19 | |
| Gasterosteus_aculeatus.groupVI.7638826.7639019 | Gasterosteus_aculeatus.groupVI.7638826.7639019 | 32 | 32 | |
| Gasterosteus_aculeatus.groupVI.7657257.7657465 | Gasterosteus_aculeatus.groupVI.7657257.7657465 | 27 | 27 | |
| Gasterosteus_aculeatus.groupVI.7852646.7852828 | Gasterosteus_aculeatus.groupVI.7852646.7852828 | 22 | 22 | |
| Gasterosteus_aculeatus.groupVI.7856797.7856978 | Gasterosteus_aculeatus.groupVI.7856797.7856978 | 23 | 23 | |
| Gasterosteus_aculeatus.groupVI.8376580.8376736 | Gasterosteus_aculeatus.groupVI.8376580.8376736 | 28 | 28 | |
| Gasterosteus_aculeatus.groupVI.9079474.9079650 | Gasterosteus_aculeatus.groupVI.9079474.9079650 | 32 | 32 | |
| Gasterosteus_aculeatus.groupVI.9206727.9206919 | Gasterosteus_aculeatus.groupVI.9206727.9206919 | 15 | 15 | |
| Gasterosteus_aculeatus.groupVII.13512188.13511784 | Gasterosteus_aculeatus.groupVII.13512188.13511784 | 35 | 35 | |
| Gasterosteus_aculeatus.groupVII.14003445.14003269 | Gasterosteus_aculeatus.groupVII.14003445.14003269 | 23 | 23 | |
| Gasterosteus_aculeatus.groupVII.14988531.14988863 | Gasterosteus_aculeatus.groupVII.14988531.14988863 | 22 | 22 | |
| Gasterosteus_aculeatus.groupVII.15811359.15811123 | Gasterosteus_aculeatus.groupVII.15811359.15811123 | 18 | 18 | |
| Gasterosteus_aculeatus.groupVII.20975145.20974921 | Gasterosteus_aculeatus.groupVII.20975145.20974921 | 36 | 36 | |
| Gasterosteus_aculeatus.groupVII.2670509.2670358 | Gasterosteus_aculeatus.groupVII.2670509.2670358 | 16 | 16 | |

| | | | | |
|---|---|---|---|---|
| Gasterosteus_aculeatus.groupVII.3940452.3941056 | Gasterosteus_aculeatus.groupVII.3940452.3941056 | 3 | 24 | multiple blast hit in proteome: Alestes_baremozetm285,Anguilla_anguillatm125,Arctozensus_risso,Argentina_sphyraena029tm,Borostomias_antarcticustm762,Coregonus_spZSM13380,Cromeria_occidentalistm583a,Dallia_pectoralisUW150693-5,Danio_rerioD15,Denticeps_clupeoidestm138,Gonorhynchus_abbreviatus098tm,Gymnotus_spZSM32021,Lepidogalaxias_salamandroidesSAM,Lovettia_sealii_CSIROtr,Mallotus_villosustm016,Nansenia_oblitaBPS1997,Rutilus_rutilus2,Rutilus_rutilustm70,Synodus_saurustm67syneve,Umbra_krameritm62,bait_sequences |
| Gasterosteus_aculeatus.groupVII.4299616.4299823 | Gasterosteus_aculeatus.groupVII.4299616.4299823 | 26 | 26 | |
| Gasterosteus_aculeatus.groupVII.7907687.7907442 | Gasterosteus_aculeatus.groupVII.7907687.7907442 | 12 | 12 | |
| Gasterosteus_aculeatus.groupVIII.11687338.11687159 | Gasterosteus_aculeatus.groupVIII.11687338.11687159 | 19 | 19 | |
| Gasterosteus_aculeatus.groupVIII.12812343.12812532 | Gasterosteus_aculeatus.groupVIII.12812343.12812532 | 13 | 13 | |
| Gasterosteus_aculeatus.groupVIII.14212893.14213111 | Gasterosteus_aculeatus.groupVIII.14212893.14213111 | 36 | 36 | |
| Gasterosteus_aculeatus.groupVIII.1734231.1734479 | Gasterosteus_aculeatus.groupVIII.1734231.1734479 | 32 | 32 | |
| Gasterosteus_aculeatus.groupVIII.17977107.17976839 | Gasterosteus_aculeatus.groupVIII.17977107.17976839 | 29 | 29 | |
| Gasterosteus_aculeatus.groupVIII.18040326.18040132 | Gasterosteus_aculeatus.groupVIII.18040326.18040132 | 29 | 29 | |
| Gasterosteus_aculeatus.groupVIII.18317606.18317823 | Gasterosteus_aculeatus.groupVIII.18317606.18317823 | 24 | 24 | |
| Gasterosteus_aculeatus.groupVIII.270118.270284 | Gasterosteus_aculeatus.groupVIII.270118.270284 | 37 | 37 | |
| Gasterosteus_aculeatus.groupX.12196749.12197054 | Gasterosteus_aculeatus.groupX.12196749.12197054 | 12 | 12 | |

| | | | | |
|---|---|---|---|---|
| Gasterosteus_aculeatus.groupX.1334192.1334033 | Gasterosteus_aculeatus.groupX.1334192.1334033 | 24 | 24 | |
| Gasterosteus_aculeatus.groupX.1334634.1334441 | Gasterosteus_aculeatus.groupX.1334634.1334441 | 21 | 21 | |
| Gasterosteus_aculeatus.groupX.8943500.8943342 | Gasterosteus_aculeatus.groupX.8943500.8943342 | 32 | 32 | |
| Gasterosteus_aculeatus.groupXI.14949685.14949481 | Gasterosteus_aculeatus.groupXI.14949685.14949481 | 42 | 42 | |
| Gasterosteus_aculeatus.groupXI.574474.574263 | Gasterosteus_aculeatus.groupXI.574474.574263 | 29 | 29 | |
| Gasterosteus_aculeatus.groupXI.591033.590815 | Gasterosteus_aculeatus.groupXI.591033.590815 | 21 | 22 | multiple blast hit in proteome: Harpadon_microchir067tm |
| Gasterosteus_aculeatus.groupXI.8161486.8161680 | Gasterosteus_aculeatus.groupXI.8161486.8161680 | 26 | 26 | |
| Gasterosteus_aculeatus.groupXII.10898024.10898206 | Gasterosteus_aculeatus.groupXII.10898024.10898206 | 35 | 35 | |
| Gasterosteus_aculeatus.groupXII.16970098.16969748 | Gasterosteus_aculeatus.groupXII.16970098.16969748 | 16 | 16 | |
| Gasterosteus_aculeatus.groupXII.171460.171266 | Gasterosteus_aculeatus.groupXII.171460.171266 | 28 | 28 | |
| Gasterosteus_aculeatus.groupXII.18252817.18253017 | Gasterosteus_aculeatus.groupXII.18252817.18253017 | 28 | 28 | |
| Gasterosteus_aculeatus.groupXII.3698994.3699162 | Gasterosteus_aculeatus.groupXII.3698994.3699162 | 17 | 17 | |
| Gasterosteus_aculeatus.groupXII.5365207.5364847 | Gasterosteus_aculeatus.groupXII.5365207.5364847 | 26 | 26 | |
| Gasterosteus_aculeatus.groupXII.5655174.5655336 | Gasterosteus_aculeatus.groupXII.5655174.5655336 | 25 | 25 | |
| Gasterosteus_aculeatus.groupXII.600852.600646 | Gasterosteus_aculeatus.groupXII.600852.600646 | 13 | 13 | |
| Gasterosteus_aculeatus.groupXII.7053581.7054279 | Gasterosteus_aculeatus.groupXII.7053581.7054279 | 31 | 31 | |
| Gasterosteus_aculeatus.groupXII.7785483.7785323 | Gasterosteus_aculeatus.groupXII.7785483.7785323 | 43 | 43 | |
| Gasterosteus_aculeatus.groupXII.7803234.7803001 | Gasterosteus_aculeatus.groupXII.7803234.7803001 | 15 | 15 | |

| | | | | |
|---|---|---|---|---|
| Gasterosteus_aculeatus.groupXII.7804594.7804403 | Gasterosteus_aculeatus.groupXII.7804594.7804403 | 32 | 32 | |
| Gasterosteus_aculeatus.groupXII.8008167.8008356 | Gasterosteus_aculeatus.groupXII.8008167.8008356 | 26 | 26 | |
| Gasterosteus_aculeatus.groupXII.9458992.9458767 | Gasterosteus_aculeatus.groupXII.9458992.9458767 | 18 | 18 | |
| Gasterosteus_aculeatus.groupXIII.12946489.12948571 | Gasterosteus_aculeatus.groupXIII.12946489.12948571 | 15 | 15 | |
| Gasterosteus_aculeatus.groupXIII.12970150.12969980 | Gasterosteus_aculeatus.groupXIII.12970150.12969980 | 39 | 39 | |
| Gasterosteus_aculeatus.groupXIII.13364869.13365070 | Gasterosteus_aculeatus.groupXIII.13364869.13365070 | 15 | 15 | |
| Gasterosteus_aculeatus.groupXIII.14813118.14813619 | Gasterosteus_aculeatus.groupXIII.14813118.14813619 | 39 | 39 | |
| Gasterosteus_aculeatus.groupXIII.14856612.14856366 | Gasterosteus_aculeatus.groupXIII.14856612.14856366 | 4 | 16 | multiple blast hit in proteome: Anguilla_anguillatm125,Astronesthes_luciferT04tm,Coregonus_spZSM13380,Distichodus_brevipinnitm559,Elops_senegalensistm175,Engraulis_encrassicolustm57,Lepidogalaxias_salamandroidesSAM,Opisthoproctus_soleatusBPS0624,Retropinna_tasmanicaCL284a,Retropinna_tasmanica_CSIROtr,Synodus_saurussn10,Umbra_krameritm62 |
| Gasterosteus_aculeatus.groupXIII.1515533.1515706 | Gasterosteus_aculeatus.groupXIII.1515533.1515706 | 10 | 10 | |
| Gasterosteus_aculeatus.groupXIII.15370097.15370261 | Gasterosteus_aculeatus.groupXIII.15370097.15370261 | 25 | 25 | |
| Gasterosteus_aculeatus.groupXIII.15446784.15446619 | Gasterosteus_aculeatus.groupXIII.15446784.15446619 | 33 | 33 | |
| Gasterosteus_aculeatus.groupXIII.3945716.3945555 | Gasterosteus_aculeatus.groupXIII.3945716.3945555 | 20 | 20 | |
| Gasterosteus_aculeatus.groupXIII.9052677.9052492 | Gasterosteus_aculeatus.groupXIII.9052677.9052492 | 39 | 39 | |
| Gasterosteus_aculeatus.groupXIII.9053121.9052949 | Gasterosteus_aculeatus.groupXIII.9053121.9052949 | 30 | 30 | |

| | | | | |
|---|---|---|---|---|
| Gasterosteus_aculeatus.groupXIII.9239398.9239602 | Gasterosteus_aculeatus.groupXIII.9239398.9239602 | 36 | 36 | |
| Gasterosteus_aculeatus.groupXIII.9253949.9254153 | Gasterosteus_aculeatus.groupXIII.9253949.9254153 | 30 | 30 | |
| Gasterosteus_aculeatus.groupXIII.9818443.9818267 | Gasterosteus_aculeatus.groupXIII.9818443.9818267 | 35 | 36 | too short: Bathylagus_euryopstm112 |
| Gasterosteus_aculeatus.groupXIV.14052768.14052605 | Gasterosteus_aculeatus.groupXIV.14052768.14052605 | 30 | 30 | |
| Gasterosteus_aculeatus.groupXIV.14997880.14997606 | Gasterosteus_aculeatus.groupXIV.14997880.14997606 | 26 | 26 | |
| Gasterosteus_aculeatus.groupXIV.3144480.3144306 | Gasterosteus_aculeatus.groupXIV.3144480.3144306 | 41 | 41 | |
| Gasterosteus_aculeatus.groupXIV.3430390.3430001 | Gasterosteus_aculeatus.groupXIV.3430390.3430001 | 16 | 30 | multiple blast hit in proteome: Alestes_baremozetm285,Anguilla_anguillatm125,Borostomias_antarcticustm762,Cromeria_occidentalistm583a,Denticeps_clupeoidestm138,Distichodus_brevipinnitm559,Elops_senegalensistm175,Limnothrissa_miodon,Nansenia_oblitaBPS1997,Opisthoproctus_soleatusBPS0624,Pecoglossus_altivelistm211,Pellonula_leonensistm238,Retropinna_tasmanicaCL284a,Retropinna_tasmanica_CSIROtr |
| Gasterosteus_aculeatus.groupXIV.5834956.5834673 | Gasterosteus_aculeatus.groupXIV.5834956.5834673 | 32 | 33 | too short: Limnothrissa_miodon |
| Gasterosteus_aculeatus.groupXIV.622775.622977 | Gasterosteus_aculeatus.groupXIV.622775.622977 | 25 | 25 | |
| Gasterosteus_aculeatus.groupXIV.7366748.7366585 | Gasterosteus_aculeatus.groupXIV.7366748.7366585 | 46 | 46 | |
| Gasterosteus_aculeatus.groupXIX.11468297.11468494 | Gasterosteus_aculeatus.groupXIX.11468297.11468494 | 28 | 28 | |
| Gasterosteus_aculeatus.groupXIX.13003493.13003645 | Gasterosteus_aculeatus.groupXIX.13003493.13003645 | 31 | 39 | multiple blast hit in proteome: Anguilla_anguillatm125,Astronesthes_luciferT04tm,Borostomias_antarcticustm762,Elops_senegalensistm175,Harpadon_microchir067tm,Hime_formosana137tm,Pantodon_bucholzi382tm,Rutilus_rutilustm70 |

| | | | | |
|---|---|---|---|---|
| Gasterosteus_aculeatus.groupXIX.14489909.14491510 | Gasterosteus_aculeatus.groupXIX.14489909.14491510 | 25 | 25 | |
| Gasterosteus_aculeatus.groupXIX.14978742.14978587 | Gasterosteus_aculeatus.groupXIX.14978742.14978587 | 34 | 34 | |
| Gasterosteus_aculeatus.groupXIX.18021050.18021246 | Gasterosteus_aculeatus.groupXIX.18021050.18021246 | 38 | 38 | |
| Gasterosteus_aculeatus.groupXIX.2223508.2223329 | Gasterosteus_aculeatus.groupXIX.2223508.2223329 | 25 | 25 | |
| Gasterosteus_aculeatus.groupXIX.2856528.2856365 | Gasterosteus_aculeatus.groupXIX.2856528.2856365 | 18 | 18 | |
| Gasterosteus_aculeatus.groupXIX.4368519.4368740 | Gasterosteus_aculeatus.groupXIX.4368519.4368740 | 25 | 26 | too short: Coregonus_spZSM13380 |
| Gasterosteus_aculeatus.groupXIX.4472606.4471968 | Gasterosteus_aculeatus.groupXIX.4472606.4471968 | 24 | 24 | |
| Gasterosteus_aculeatus.groupXIX.514639.514801 | Gasterosteus_aculeatus.groupXIX.514639.514801 | 41 | 41 | |
| Gasterosteus_aculeatus.groupXIX.6655020.6654830 | Gasterosteus_aculeatus.groupXIX.6655020.6654830 | 19 | 19 | |
| Gasterosteus_aculeatus.groupXIX.7096256.7096079 | Gasterosteus_aculeatus.groupXIX.7096256.7096079 | 24 | 24 | |
| Gasterosteus_aculeatus.groupXIX.7413196.7412922 | Gasterosteus_aculeatus.groupXIX.7413196.7412922 | 30 | 30 | |
| Gasterosteus_aculeatus.groupXV.12177379.12177549 | Gasterosteus_aculeatus.groupXV.12177379.12177549 | 33 | 33 | |
| Gasterosteus_aculeatus.groupXV.15970829.15971071 | Gasterosteus_aculeatus.groupXV.15970829.15971071 | 32 | 32 | |
| Gasterosteus_aculeatus.groupXV.298005.297825 | Gasterosteus_aculeatus.groupXV.298005.297825 | 20 | 20 | |
| Gasterosteus_aculeatus.groupXV.314584.315141 | Gasterosteus_aculeatus.groupXV.314584.315141 | 35 | 35 | |
| Gasterosteus_aculeatus.groupXV.5417677.5417494 | Gasterosteus_aculeatus.groupXV.5417677.5417494 | 18 | 18 | |
| Gasterosteus_aculeatus.groupXV.9696816.9696604 | Gasterosteus_aculeatus.groupXV.9696816.9696604 | 30 | 30 | |
| Gasterosteus_aculeatus.groupXVI.10656059.10655902 | Gasterosteus_aculeatus.groupXVI.10656059.10655902 | 27 | 27 | |

| | | | | |
|---|---|---|---|---|
| Gasterosteus_aculeatus.groupXVI.10867687.10867848 | Gasterosteus_aculeatus.groupXVI.10867687.10867848 | 31 | 31 | |
| Gasterosteus_aculeatus.groupXVI.10906239.10905548 | Gasterosteus_aculeatus.groupXVI.10906239.10905548 | 20 | 20 | |
| Gasterosteus_aculeatus.groupXVI.12886392.12886215 | Gasterosteus_aculeatus.groupXVI.12886392.12886215 | 19 | 19 | |
| Gasterosteus_aculeatus.groupXVI.15356340.15356174 | Gasterosteus_aculeatus.groupXVI.15356340.15356174 | 12 | 12 | |
| Gasterosteus_aculeatus.groupXVI.16912971.16913135 | Gasterosteus_aculeatus.groupXVI.16912971.16913135 | 10 | 10 | |
| Gasterosteus_aculeatus.groupXVI.17881136.17881303 | Gasterosteus_aculeatus.groupXVI.17881136.17881303 | 40 | 40 | |
| Gasterosteus_aculeatus.groupXVI.2437743.2437958 | Gasterosteus_aculeatus.groupXVI.2437743.2437958 | 29 | 29 | |
| Gasterosteus_aculeatus.groupXVI.2476903.2477059 | Gasterosteus_aculeatus.groupXVI.2476903.2477059 | 40 | 40 | |
| Gasterosteus_aculeatus.groupXVI.6356286.6355819 | Gasterosteus_aculeatus.groupXVI.6356286.6355819 | 20 | 20 | |
| Gasterosteus_aculeatus.groupXVI.6441779.6441954 | Gasterosteus_aculeatus.groupXVI.6441779.6441954 | 25 | 25 | |
| Gasterosteus_aculeatus.groupXVI.7739035.7738841 | Gasterosteus_aculeatus.groupXVI.7739035.7738841 | 32 | 32 | |
| Gasterosteus_aculeatus.groupXVII.447443.447600 | Gasterosteus_aculeatus.groupXVII.447443.447600 | 10 | 10 | |
| Gasterosteus_aculeatus.groupXVIII.10859578.10859415 | Gasterosteus_aculeatus.groupXVIII.10859578.10859415 | 27 | 27 | |
| Gasterosteus_aculeatus.groupXVIII.11082912.11082460 | Gasterosteus_aculeatus.groupXVIII.11082912.11082460 | 25 | 25 | |
| Gasterosteus_aculeatus.groupXVIII.11212752.11212952 | Gasterosteus_aculeatus.groupXVIII.11212752.11212952 | 17 | 17 | |
| Gasterosteus_aculeatus.groupXVIII.11496186.11496400 | Gasterosteus_aculeatus.groupXVIII.11496186.11496400 | 10 | 10 | |
| Gasterosteus_aculeatus.groupXVIII.12492229.12492059 | Gasterosteus_aculeatus.groupXVIII.12492229.12492059 | 39 | 39 | |
| Gasterosteus_aculeatus.groupXVIII.15272055.15272271 | Gasterosteus_aculeatus.groupXVIII.15272055.15272271 | 22 | 22 | |

| | | | | |
|---|---|---|---|---|
| Gasterosteus_aculeatus.groupXVIII.15542408.15542592 | Gasterosteus_aculeatus.groupXVIII.15542408.15542592 | 27 | 27 | |
| Gasterosteus_aculeatus.groupXVIII.3245898.3245650 | Gasterosteus_aculeatus.groupXVIII.3245898.3245650 | 21 | 21 | |
| Gasterosteus_aculeatus.groupXVIII.5505446.5505699 | Gasterosteus_aculeatus.groupXVIII.5505446.5505699 | 14 | 14 | |
| Gasterosteus_aculeatus.groupXVIII.5506526.5506933 | Gasterosteus_aculeatus.groupXVIII.5506526.5506933 | 30 | 30 | |
| Gasterosteus_aculeatus.groupXVIII.9374575.9374747 | Gasterosteus_aculeatus.groupXVIII.9374575.9374747 | 23 | 23 | |
| Gasterosteus_aculeatus.groupXX.10526127.10525955 | Gasterosteus_aculeatus.groupXX.10526127.10525955 | 40 | 40 | |
| Gasterosteus_aculeatus.groupXX.12725449.12725273 | Gasterosteus_aculeatus.groupXX.12725449.12725273 | 37 | 37 | |
| Gasterosteus_aculeatus.groupXX.13427022.13426822 | Gasterosteus_aculeatus.groupXX.13427022.13426822 | 31 | 32 | too short: Rutilus_rutilustm70 |
| Gasterosteus_aculeatus.groupXX.14253717.14253497 | Gasterosteus_aculeatus.groupXX.14253717.14253497 | 22 | 22 | |
| Gasterosteus_aculeatus.groupXX.15630217.15630386 | Gasterosteus_aculeatus.groupXX.15630217.15630386 | 28 | 28 | |
| Gasterosteus_aculeatus.groupXX.158613.159330 | Gasterosteus_aculeatus.groupXX.158613.159330 | 25 | 26 | multiple blast hit in proteome: Gonorhynchus_abbreviatus098tm |
| Gasterosteus_aculeatus.groupXX.164552.164755 | Gasterosteus_aculeatus.groupXX.164552.164755 | 15 | 15 | |
| Gasterosteus_aculeatus.groupXX.17377835.17377617 | Gasterosteus_aculeatus.groupXX.17377835.17377617 | 11 | 11 | |
| Gasterosteus_aculeatus.groupXX.17383619.17383395 | Gasterosteus_aculeatus.groupXX.17383619.17383395 | 32 | 32 | |
| Gasterosteus_aculeatus.groupXX.17781392.17780290 | Gasterosteus_aculeatus.groupXX.17781392.17780290 | 22 | 22 | |
| Gasterosteus_aculeatus.groupXX.18653582.18653731 | Gasterosteus_aculeatus.groupXX.18653582.18653731 | 20 | 20 | |
| Gasterosteus_aculeatus.groupXX.3258847.3258688 | Gasterosteus_aculeatus.groupXX.3258847.3258688 | 23 | 23 | |
| Gasterosteus_aculeatus.groupXX.8138072.8138252 | Gasterosteus_aculeatus.groupXX.8138072.8138252 | 43 | 43 | |

| | | | | |
|---|---|---|---|---|
| Gasterosteus_aculeatus.groupXX.9099651.9099845 | Gasterosteus_aculeatus.groupXX.9099651.9099845 | 24 | 24 | |
| Gasterosteus_aculeatus.groupXXI.11189417.11189598 | Gasterosteus_aculeatus.groupXXI.11189417.11189598 | 27 | 27 | |
| Gasterosteus_aculeatus.groupXXI.11663956.11664125 | Gasterosteus_aculeatus.groupXXI.11663956.11664125 | 36 | 36 | |
| Gasterosteus_aculeatus.groupXXI.2138619.2138416 | Gasterosteus_aculeatus.groupXXI.2138619.2138416 | 14 | 14 | |
| Gasterosteus_aculeatus.groupXXI.3882897.3882724 | Gasterosteus_aculeatus.groupXXI.3882897.3882724 | 24 | 24 | |
| Gasterosteus_aculeatus.groupXXI.5769896.5769739 | Gasterosteus_aculeatus.groupXXI.5769896.5769739 | 20 | 20 | |
| Gasterosteus_aculeatus.groupXXI.9664054.9664211 | Gasterosteus_aculeatus.groupXXI.9664054.9664211 | 23 | 23 | |
| Gasterosteus_aculeatus.scaffold_1238.3012.2848 | Gasterosteus_aculeatus.scaffold_1238.3012.2848 | 41 | 41 | |
| Gasterosteus_aculeatus.scaffold_126.152577.152801 | Gasterosteus_aculeatus.scaffold_126.152577.152801 | 29 | 29 | |
| Gasterosteus_aculeatus.scaffold_211.77905.77750 | Gasterosteus_aculeatus.scaffold_211.77905.77750 | 38 | 38 | |
| Gasterosteus_aculeatus.scaffold_27.1038639.1038391 | Gasterosteus_aculeatus.scaffold_27.1038639.1038391 | 39 | 39 | |
| Gasterosteus_aculeatus.scaffold_27.2151985.2152141 | Gasterosteus_aculeatus.scaffold_27.2151985.2152141 | 28 | 28 | |
| Gasterosteus_aculeatus.scaffold_324.12639.12418 | Gasterosteus_aculeatus.scaffold_324.12639.12418 | 28 | 28 | |
| Gasterosteus_aculeatus.scaffold_48.1209822.1209595 | Gasterosteus_aculeatus.scaffold_48.1209822.1209595 | 26 | 26 | |
| Gasterosteus_aculeatus.scaffold_56.59563.59761 | Gasterosteus_aculeatus.scaffold_56.59563.59761 | 38 | 38 | |
| Gasterosteus_aculeatus.scaffold_74.312198.312040 | Gasterosteus_aculeatus.scaffold_74.312198.312040 | 17 | 17 | |
| Gasterosteus_aculeatus.scaffold_76.225290.225088 | Gasterosteus_aculeatus.scaffold_76.225290.225088 | 17 | 17 | |
| Gasterosteus_aculeatus.scaffold_80.409886.409231 | Gasterosteus_aculeatus.scaffold_80.409886.409231 | 21 | 21 | |

| | | | | |
|---|---|---|---|---|
| Gasterosteus_aculeatus.scaffold_84.66826.67115 | Gasterosteus_aculeatus.scaffold_84.66826.67115 | 28 | 28 | |
| Gasterosteus_aculeatus.scaffold_88.93192.93019 | Gasterosteus_aculeatus.scaffold_88.93192.93019 | 28 | 28 | |
| Gasterosteus_aculeatus.scaffold_89.284328.284518 | Gasterosteus_aculeatus.scaffold_89.284328.284518 | 27 | 27 | |
| Gasterosteus_aculeatus.scaffold_98.36345.36113 | Gasterosteus_aculeatus.scaffold_98.36345.36113 | 28 | 28 | |
| Oryzias_latipes.1.15026039.15026243 | Oryzias_latipes.1.15026039.15026243 | 30 | 30 | |
| Oryzias_latipes.1.19523985.19522215 | Oryzias_latipes.1.19523985.19522215 | 21 | 21 | |
| Oryzias_latipes.1.19526200.19525852 | Oryzias_latipes.1.19526200.19525852 | 25 | 25 | |
| Oryzias_latipes.1.2307747.2307541 | Oryzias_latipes.1.2307747.2307541 | 23 | 23 | |
| Oryzias_latipes.1.2473136.2472909 | Oryzias_latipes.1.2473136.2472909 | 22 | 22 | |
| Oryzias_latipes.1.34004068.34003856 | Oryzias_latipes.1.34004068.34003856 | 28 | 28 | |
| Oryzias_latipes.1.37192141.37192333 | Oryzias_latipes.1.37192141.37192333 | 25 | 25 | |
| Oryzias_latipes.10.12010077.12009892 | Oryzias_latipes.10.12010077.12009892 | 31 | 31 | |
| Oryzias_latipes.10.13882797.13882960 | Oryzias_latipes.10.13882797.13882960 | 40 | 40 | |
| Oryzias_latipes.10.6811316.6812001 | Oryzias_latipes.10.6811316.6812001 | 36 | 36 | |
| Oryzias_latipes.10.6812140.6812383 | Oryzias_latipes.10.6812140.6812383 | 33 | 33 | |
| Oryzias_latipes.11.16322344.16322156 | Oryzias_latipes.11.16322344.16322156 | 25 | 25 | |
| Oryzias_latipes.11.17363490.17363654 | Oryzias_latipes.11.17363490.17363654 | 38 | 38 | |
| Oryzias_latipes.11.18479350.18479156 | Oryzias_latipes.11.18479350.18479156 | 33 | 33 | |
| Oryzias_latipes.11.21534604.21534305 | Oryzias_latipes.11.21534604.21534305 | 19 | 20 | multiple blast hit in proteome: Galaxias_fasciatusCL282 |

| | | | | |
|---|---|---|---|---|
| Oryzias_latipes.11.22842523.22842024 | Oryzias_latipes.11.22842523.22842024 | 11 | 18 | multiple blast hit in proteome: Alepocephalus_bicolor,Alepocephalus_brevipinnitm66merge,Denticeps_clupeoidestm138,Evermanella_balbotm67eve,Galaxias_fasciatusCL282,Pantodon_bucholzi382tm,Synodus_saurustm67syneve |
| Oryzias_latipes.11.9811901.9811544 | Oryzias_latipes.11.9811901.9811544 | 37 | 37 | |
| Oryzias_latipes.12.2022363.2022540 | Oryzias_latipes.12.2022363.2022540 | 21 | 21 | |
| Oryzias_latipes.12.20251518.20251853 | Oryzias_latipes.12.20251518.20251853 | 24 | 24 | |
| Oryzias_latipes.12.20473412.20473606 | Oryzias_latipes.12.20473412.20473606 | 24 | 24 | |
| Oryzias_latipes.12.3724187.3723980 | Oryzias_latipes.12.3724187.3723980 | 0 | 38 | multiple blast hit in proteome: Alepocephalus_bicolor,Alestes_baremozetm285,Bathylagus_euryopstm112,Borostomias_antarcticustm762,Chanos_chanostm1777,Coregonus_spZSM13380,Cromeria_occidentalistm583a,Dallia_pectoralisUW150693-5,Danio_rerioD15,Denticeps_clupeoidestm138,Distichodus_brevipinnitm559,Engraulis_encrassicolustm57,Evermanella_balbotm67eve,Galaxias_fasciatusCL282,Gonorhynchus_abbreviatus098tm,Gymnotus_spZSM32021,Harpadon_microchir067tm,Holtbyrnia_anomalatm117,Ijimaia_loppeiBPS2503,Lepidogalaxias_salamandroidesSAM,Limnothrissa_miodon,Lovettia_sealii_CSIROtr,Mallotus_villosustm016,Maurolicus_muelleritm2013W,Nansenia_oblitaBPS1997,Opisthoproctus_soleatusBPS0624,Pantodon_bucholzi382tm,Pecoglossus_altivelistm211,Retropinna_tasmanicaCL284a,Retropinna_tasmanica_CSIROtr,Rutilus_rutilus2,Rutilus_rutilustm70,Salanx_acuticepstmT02,Schilbe_mystustm551b,Synodus_saurussn10,Synodus_saurustm67syneve,Umbra_krameritm62,bait_sequences |

| | | | | |
|---|---|---|---|---|
| Oryzias_latipes.12.6932069.6932758 | Oryzias_latipes.12.6932069.6932758 | 37 | 37 | |
| Oryzias_latipes.12.8987342.8987554 | Oryzias_latipes.12.8987342.8987554 | 28 | 29 | too short: Rutilus_rutilustm70 |
| Oryzias_latipes.12.9727776.9727941 | Oryzias_latipes.12.9727776.9727941 | 18 | 41 | multiple blast hit in proteome: Arctozensus_risso,Astronesthes_luciferT04tm,Borostomias_antarcticustm762,Coregonus_spZSM13380,Cromeria_occidentalistm583a,Dallia_pectoralisUW150693-5,Engraulis_encrassicolustm57,Galaxias_fasciatusCL282,Gymnotus_spZSM32021,Hime_formosana137tm,Lepidogalaxias_salamandroidesSAM,Limnothrissa_miodon,Mallotus_villosustm016,Nansenia_oblitaBPS1997,Opisthoproctus_soleatusBPS0624,Osmerus_eperlanustm785,Pantodon_bucholzi382tm,Pecoglossus_altivelistm211,Retropinna_tasmanicaCL284a,Retropinna_tasmanica_CSIROtr,Salanx_acuticepstmT02,Salmo_truttatm20154,Synodus_saurussn10 |
| Oryzias_latipes.12.9993416.9993621 | Oryzias_latipes.12.9993416.9993621 | 18 | 18 | |
| Oryzias_latipes.13.18786381.18786603 | Oryzias_latipes.13.18786381.18786603 | 22 | 22 | |
| Oryzias_latipes.13.2720656.2720856 | Oryzias_latipes.13.2720656.2720856 | 40 | 40 | |
| Oryzias_latipes.13.29951718.29951519 | Oryzias_latipes.13.29951718.29951519 | 19 | 19 | |
| Oryzias_latipes.13.4205418.4205155 | Oryzias_latipes.13.4205418.4205155 | 26 | 26 | |
| Oryzias_latipes.13.9526103.9525880 | Oryzias_latipes.13.9526103.9525880 | 18 | 18 | |
| Oryzias_latipes.14.12776522.12776244 | Oryzias_latipes.14.12776522.12776244 | 24 | 24 | |
| Oryzias_latipes.14.13156289.13156047 | Oryzias_latipes.14.13156289.13156047 | 32 | 32 | |
| Oryzias_latipes.14.13233375.13232708 | Oryzias_latipes.14.13233375.13232708 | 37 | 37 | |
| Oryzias_latipes.14.28402133.28402310 | Oryzias_latipes.14.28402133.28402310 | 29 | 29 | |
| Oryzias_latipes.15.11955940.11956249 | Oryzias_latipes.15.11955940.11956249 | 38 | 38 | |

| | | | | |
|---|---|---|---|---|
| Oryzias_latipes.15.13689346.13689181 | Oryzias_latipes.15.13689346.13689181 | 17 | 17 | |
| Oryzias_latipes.15.21752333.21752150 | Oryzias_latipes.15.21752333.21752150 | 10 | 10 | |
| Oryzias_latipes.15.22908869.22908652 | Oryzias_latipes.15.22908869.22908652 | 25 | 25 | |
| Oryzias_latipes.15.28637630.28637811 | Oryzias_latipes.15.28637630.28637811 | 23 | 23 | |
| Oryzias_latipes.15.29322568.29322720 | Oryzias_latipes.15.29322568.29322720 | 18 | 18 | |
| Oryzias_latipes.15.29399660.29400785 | Oryzias_latipes.15.29399660.29400785 | 24 | 25 | multiple blast hit in proteome: Rutilus_rutilustm70 |
| Oryzias_latipes.15.3044406.3044774 | Oryzias_latipes.15.3044406.3044774 | 16 | 16 | |
| Oryzias_latipes.15.3640508.3640297 | Oryzias_latipes.15.3640508.3640297 | 25 | 25 | |
| Oryzias_latipes.15.8773287.8772959 | Oryzias_latipes.15.8773287.8772959 | 21 | 21 | |
| Oryzias_latipes.16.13676115.13675816 | Oryzias_latipes.16.13676115.13675816 | 38 | 38 | |
| Oryzias_latipes.16.16989291.16989131 | Oryzias_latipes.16.16989291.16989131 | 12 | 12 | |
| Oryzias_latipes.16.20848349.20848161 | Oryzias_latipes.16.20848349.20848161 | 21 | 21 | |
| Oryzias_latipes.16.5768531.5768378 | Oryzias_latipes.16.5768531.5768378 | 11 | 11 | |
| Oryzias_latipes.16.6252990.6253995 | Oryzias_latipes.16.6252990.6253995 | 33 | 33 | |
| Oryzias_latipes.17.12644643.12644494 | Oryzias_latipes.17.12644643.12644494 | 36 | 36 | |
| Oryzias_latipes.17.23751616.23751831 | Oryzias_latipes.17.23751616.23751831 | 28 | 28 | |
| Oryzias_latipes.17.28361251.28361049 | Oryzias_latipes.17.28361251.28361049 | 22 | 22 | |
| Oryzias_latipes.17.28760022.28760420 | Oryzias_latipes.17.28760022.28760420 | 21 | 21 | |
| Oryzias_latipes.17.29352543.29353108 | Oryzias_latipes.17.29352543.29353108 | 21 | 28 | multiple blast hit in proteome: Argentina_sphyraena029tm,Coregonus_spZSM13380,Hime_formosana137tm,Lepidogalaxias_salamandroidesSAM,Mallotus_villosustm016,Retropinna_tasmanicaCL284a,Salmo_truttatm20154 |
| Oryzias_latipes.18.11242846.11242627 | Oryzias_latipes.18.11242846.11242627 | 18 | 18 | |

| | | | | |
|---|---|---|---|---|
| Oryzias_latipes.18.19040432.19039986 | Oryzias_latipes.18.19040432.19039986 | 21 | 21 | |
| Oryzias_latipes.18.22521969.22522200 | Oryzias_latipes.18.22521969.22522200 | 25 | 25 | |
| Oryzias_latipes.19.17937482.17937658 | Oryzias_latipes.19.17937482.17937658 | 16 | 16 | |
| Oryzias_latipes.19.1963504.1963217 | Oryzias_latipes.19.1963504.1963217 | 19 | 19 | |
| Oryzias_latipes.19.4267754.4267948 | Oryzias_latipes.19.4267754.4267948 | 30 | 30 | |
| Oryzias_latipes.19.8964514.8964728 | Oryzias_latipes.19.8964514.8964728 | 28 | 28 | |
| Oryzias_latipes.19.9754042.9754206 | Oryzias_latipes.19.9754042.9754206 | 26 | 26 | |
| Oryzias_latipes.2.5013211.5013059 | Oryzias_latipes.2.5013211.5013059 | 26 | 26 | |
| Oryzias_latipes.20.15269857.15268841 | Oryzias_latipes.20.15269857.15268841 | 25 | 25 | |
| Oryzias_latipes.20.15626365.15626204 | Oryzias_latipes.20.15626365.15626204 | 38 | 38 | |
| Oryzias_latipes.20.17308022.17307696 | Oryzias_latipes.20.17308022.17307696 | 30 | 30 | |
| Oryzias_latipes.20.1884685.1884503 | Oryzias_latipes.20.1884685.1884503 | 29 | 29 | |
| Oryzias_latipes.20.19959910.19959756 | Oryzias_latipes.20.19959910.19959756 | 25 | 25 | |
| Oryzias_latipes.20.22296673.22296892 | Oryzias_latipes.20.22296673.22296892 | 23 | 23 | |
| Oryzias_latipes.21.10045878.10045720 | Oryzias_latipes.21.10045878.10045720 | 26 | 26 | |
| Oryzias_latipes.21.13499256.13499411 | Oryzias_latipes.21.13499256.13499411 | 28 | 28 | |
| Oryzias_latipes.21.13512823.13513027 | Oryzias_latipes.21.13512823.13513027 | 20 | 20 | |
| Oryzias_latipes.21.24051290.24051460 | Oryzias_latipes.21.24051290.24051460 | 21 | 21 | |
| Oryzias_latipes.21.24592238.24591830 | Oryzias_latipes.21.24592238.24591830 | 31 | 31 | |
| Oryzias_latipes.21.26530244.26530047 | Oryzias_latipes.21.26530244.26530047 | 22 | 22 | |
| Oryzias_latipes.21.30715701.30715535 | Oryzias_latipes.21.30715701.30715535 | 25 | 25 | |
| Oryzias_latipes.21.30979450.30979680 | Oryzias_latipes.21.30979450.30979680 | 14 | 14 | |
| Oryzias_latipes.21.6683195.6682965 | Oryzias_latipes.21.6683195.6682965 | 27 | 28 | too short: Rutilus_rutilus2 |

| | | | | |
|---|---|---|---|---|
| Oryzias_latipes.22.11783180.11783001 | Oryzias_latipes.22.11783180.11783001 | 11 | 11 | |
| Oryzias_latipes.22.12394164.12393881 | Oryzias_latipes.22.12394164.12393881 | 23 | 23 | |
| Oryzias_latipes.22.13195804.13195195 | Oryzias_latipes.22.13195804.13195195 | 30 | 30 | |
| Oryzias_latipes.22.1863645.1863170 | Oryzias_latipes.22.1863645.1863170 | 17 | 17 | |
| Oryzias_latipes.22.3621848.3621225 | Oryzias_latipes.22.3621848.3621225 | 25 | 25 | |
| Oryzias_latipes.22.5282860.5282654 | Oryzias_latipes.22.5282860.5282654 | 12 | 12 | |
| Oryzias_latipes.22.7044219.7044460 | Oryzias_latipes.22.7044219.7044460 | 31 | 31 | |
| Oryzias_latipes.22.9003489.9003670 | Oryzias_latipes.22.9003489.9003670 | 16 | 16 | |
| Oryzias_latipes.23.13312579.13312349 | Oryzias_latipes.23.13312579.13312349 | 46 | 46 | |
| Oryzias_latipes.23.17635035.17635198 | Oryzias_latipes.23.17635035.17635198 | 28 | 28 | |

| | | | | |
|---|---|---|---|---|
| Oryzias_latipes.23.17652060.17651641 | Oryzias_latipes.23.17652060.17651641 | 3 | 40 | multiple blast hit in proteome: Alepocephalus_bicolor,Alepocephalus_brevipinnitm66merge,Alestes_baremozetm285,Astronesthes_luciferT04tm,Ateleopus_japonicus133tm,Bathylagus_euryopstm112,Borostomias_antarcticustm762,Chanos_chanostm1777,Clupea_harengustm100,Coregonus_spZSM13380,Cromeria_occidentalistm583a,Dallia_pectoralisUW150693-5,Danio_rerioD15,Denticeps_clupeoidestm138,Dussumiera_elopsoides037tm,Esox_luciustm052,Gonorhynchus_abbreviatus098tm,Gymnotus_spZSM32021,Harpadon_microchir067tm,Hime_formosana137tm,Ijimaia_loppeiBPS2503,Lestrolepis_japonicustmT03,Lepidogalaxias_salamandroidesSAM,Limnothrissa_miodon,Lovettia_sealii_CSIROtr,Mallotus_villosustm016,Maurolicus_muelleritm2013W,Opisthoproctus_soleatusBPS0624,Osmerus_eperlanustm785,Pantodon_bucholzi382tm,Pecoglossus_altivelistm211,Pellonula_leonensistm238,Retropinna_tasmanicaCL284a,Salanx_acuticepstmT02,Synodus_saurussn10,Synodus_saurustm67syneve,bait_sequences |
| Oryzias_latipes.24.19556613.19556080 | Oryzias_latipes.24.19556613.19556080 | 26 | 26 | |
| Oryzias_latipes.24.21067167.21066969 | Oryzias_latipes.24.21067167.21066969 | 25 | 25 | |
| Oryzias_latipes.24.21099756.21099484 | Oryzias_latipes.24.21099756.21099484 | 32 | 32 | |
| Oryzias_latipes.24.2341263.2341413 | Oryzias_latipes.24.2341263.2341413 | 21 | 21 | |
| Oryzias_latipes.24.2576369.2576940 | Oryzias_latipes.24.2576369.2576940 | 27 | 27 | |
| Oryzias_latipes.24.3902152.3901865 | Oryzias_latipes.24.3902152.3901865 | 16 | 16 | |
| Oryzias_latipes.24.6871007.6870826 | Oryzias_latipes.24.6871007.6870826 | 17 | 17 | |
| Oryzias_latipes.24.9598065.9598309 | Oryzias_latipes.24.9598065.9598309 | 21 | 21 | |

| | | | | |
|---|---|---|---|---|
| Oryzias_latipes.3.13211829.13212028 | Oryzias_latipes.3.13211829.13212028 | 36 | 36 | |
| Oryzias_latipes.3.14907968.14908163 | Oryzias_latipes.3.14907968.14908163 | 21 | 21 | |
| Oryzias_latipes.3.14952723.14952551 | Oryzias_latipes.3.14952723.14952551 | 34 | 34 | |
| Oryzias_latipes.3.15769992.15770233 | Oryzias_latipes.3.15769992.15770233 | 21 | 21 | |
| Oryzias_latipes.3.16126437.16126196 | Oryzias_latipes.3.16126437.16126196 | 35 | 35 | |
| Oryzias_latipes.3.18497594.18497421 | Oryzias_latipes.3.18497594.18497421 | 22 | 22 | |
| Oryzias_latipes.3.19258428.19258194 | Oryzias_latipes.3.19258428.19258194 | 25 | 25 | |
| Oryzias_latipes.3.19970055.19970421 | Oryzias_latipes.3.19970055.19970421 | 37 | 37 | |
| Oryzias_latipes.3.24539913.24540096 | Oryzias_latipes.3.24539913.24540096 | 17 | 17 | |
| Oryzias_latipes.3.32577926.32577701 | Oryzias_latipes.3.32577926.32577701 | 20 | 20 | |
| Oryzias_latipes.3.35145903.35145738 | Oryzias_latipes.3.35145903.35145738 | 17 | 17 | |
| Oryzias_latipes.4.12032215.12032372 | Oryzias_latipes.4.12032215.12032372 | 21 | 21 | |

| | | | | |
|---|---|---|---|---|
| Oryzias_latipes.4.18480485.18480720 | Oryzias_latipes.4.18480485.18480720 | 6 | 45 | multiple blast hit in proteome: Alepocephalus_bicolor,Alepocephalus_brevipinnitm66merge,Alestes_baremozetm285,Arctozensus_risso,Argentina_sphyraena029tm,Astronesthes_luciferT04tm,Ateleopus_japonicus133tm,Borostomias_antarcticustm762,Chanos_chanostm1777,Clupea_harengustm100,Coregonus_spZSM13380,Dallia_pectoralisUW150693-5,Dussumiera_elopsoides037tm,Elops_senegalensistm175,Engraulis_encrassicolustm57,Esox_luciustm052,Evermanella_balbotm67eve,Galaxias_fasciatusCL282,Harpadon_microchir067tm,Hime_formosana137tm,Ijimaia_loppeiBPS2503,Lestrolepis_japonicustmT03,Lepidogalaxias_salamandroidesSAM,Limnothrissa_miodon,Lovettia_sealii_CSIROtr,Mallotus_villosustm016,Maurolicus_muelleritm2013W,Nansenia_oblitaBPS1997,Opisthoproctus_soleatusBPS0624,Pellonula_leonensistm238,Retropinna_tasmanicaCL284a,Retropinna_tasmanica_CSIROtr,Rutilus_rutilus2,Rutilus_rutilustm70,Salanx_acuticepstmT02,Schilbe_mystustm551b,Synodus_saurussn10,Synodus_saurustm67syneve,Umbra_krameritm62 |
| Oryzias_latipes.4.1998697.1998882 | Oryzias_latipes.4.1998697.1998882 | 20 | 20 | |
| Oryzias_latipes.4.30225238.30225046 | Oryzias_latipes.4.30225238.30225046 | 26 | 26 | |
| Oryzias_latipes.4.3513243.3513064 | Oryzias_latipes.4.3513243.3513064 | 22 | 23 | multiple blast hit in proteome: Rutilus_rutilustm70 |
| Oryzias_latipes.4.5823361.5823512 | Oryzias_latipes.4.5823361.5823512 | 34 | 34 | |
| Oryzias_latipes.5.14676645.14676811 | Oryzias_latipes.5.14676645.14676811 | 35 | 35 | |
| Oryzias_latipes.5.25915342.25915513 | Oryzias_latipes.5.25915342.25915513 | 26 | 26 | |
| Oryzias_latipes.5.26580699.26580187 | Oryzias_latipes.5.26580699.26580187 | 19 | 19 | |

| | | | | |
|---|---|---|---|---|
| Oryzias_latipes.5.29836259.29835240 | Oryzias_latipes.5.29836259.29835240 | 19 | 19 | |
| Oryzias_latipes.6.11042569.11042370 | Oryzias_latipes.6.11042569.11042370 | 26 | 26 | |
| Oryzias_latipes.6.11602750.11603351 | Oryzias_latipes.6.11602750.11603351 | 29 | 29 | |
| Oryzias_latipes.6.14272976.14273169 | Oryzias_latipes.6.14272976.14273169 | 30 | 30 | |
| Oryzias_latipes.6.16667564.16667764 | Oryzias_latipes.6.16667564.16667764 | 23 | 23 | |
| Oryzias_latipes.6.20527840.20528047 | Oryzias_latipes.6.20527840.20528047 | 25 | 25 | |
| Oryzias_latipes.6.21146270.21145984 | Oryzias_latipes.6.21146270.21145984 | 24 | 24 | |
| Oryzias_latipes.6.26475530.26475719 | Oryzias_latipes.6.26475530.26475719 | 28 | 28 | |
| Oryzias_latipes.6.2850371.2850522 | Oryzias_latipes.6.2850371.2850522 | 32 | 32 | |
| Oryzias_latipes.6.2850670.2850876 | Oryzias_latipes.6.2850670.2850876 | 16 | 16 | |
| Oryzias_latipes.6.286110.286304 | Oryzias_latipes.6.286110.286304 | 14 | 14 | |
| Oryzias_latipes.6.584465.584276 | Oryzias_latipes.6.584465.584276 | 36 | 36 | |
| Oryzias_latipes.6.6538732.6539544 | Oryzias_latipes.6.6538732.6539544 | 25 | 25 | |
| Oryzias_latipes.7.10498742.10498445 | Oryzias_latipes.7.10498742.10498445 | 30 | 30 | |

| | | | | |
|---|---|---|---|---|
| Oryzias_latipes.7.11945142.11945428 | Oryzias_latipes.7.11945142.11945428 | 6 | 28 | multiple blast hit in proteome: Arctozensus_risso,Argentina_sphyraena029tm,Chanos_chanostm1777,Clupea_harengustm100,Coregonus_spZSM13380,Cromeria_occidentalistm583a,Distichodus_brevipinnitm559,Elops_senegalensistm175,Engraulis_encrassicolustm57,Evermanella_balbotm67eve,Galaxias_fasciatusCL282,Gymnotus_spZSM32021,Harpadon_microchir067tm,Lepidogalaxias_salamandroidesSAM,Limnothrissa_miodon,Nansenia_oblitaBPS1997,Opisthoproctus_soleatusBPS0624,Osmerus_eperlanustm785,Pecoglossus_altivelistm211,Retropinna_tasmanicaCL284a,Retropinna_tasmanica_CSIROtr,Umbra_krameritm62 |
| Oryzias_latipes.7.21423243.21422780 | Oryzias_latipes.7.21423243.21422780 | 28 | 28 | |
| Oryzias_latipes.7.5706062.5706217 | Oryzias_latipes.7.5706062.5706217 | 42 | 42 | |
| Oryzias_latipes.7.635525.635250 | Oryzias_latipes.7.635525.635250 | 43 | 43 | |
| Oryzias_latipes.7.636033.635833 | Oryzias_latipes.7.636033.635833 | 34 | 34 | |
| Oryzias_latipes.8.11158294.11158047 | Oryzias_latipes.8.11158294.11158047 | 27 | 27 | |
| Oryzias_latipes.8.12232553.12232783 | Oryzias_latipes.8.12232553.12232783 | 25 | 25 | |
| Oryzias_latipes.8.4463787.4463192 | Oryzias_latipes.8.4463787.4463192 | 10 | 25 | multiple blast hit in proteome: Alestes_baremozetm285,Argentina_sphyraena029tm,Chanos_chanostm1777,Coregonus_spZSM13380,Denticeps_clupeoidestm138,Engraulis_encrassicolustm57,Gymnotus_spZSM32021,Lepidogalaxias_salamandroidesSAM,Lovettia_sealii_CSIROtr,Mallotus_villosustm016,Nansenia_oblitaBPS1997,Osmerus_eperlanustm785,Rutilus_rutilus2,Rutilus_rutilustm70 |
| Oryzias_latipes.9.11144262.11144683 | Oryzias_latipes.9.11144262.11144683 | 25 | 25 | |

| | | | | |
|---|---|---|---|---|
| Oryzias_latipes.9.12477894.12477076 | Oryzias_latipes.9.12477894.12477076 | 16 | 16 | |
| Oryzias_latipes.9.13209053.13209258 | Oryzias_latipes.9.13209053.13209258 | 17 | 17 | |
| Oryzias_latipes.9.18696088.18695938 | Oryzias_latipes.9.18696088.18695938 | 32 | 32 | |
| Oryzias_latipes.9.2024905.2024732 | Oryzias_latipes.9.2024905.2024732 | 40 | 40 | |
| Oryzias_latipes.9.23006487.23006750 | Oryzias_latipes.9.23006487.23006750 | 29 | 29 | |
| Oryzias_latipes.9.23034084.23033912 | Oryzias_latipes.9.23034084.23033912 | 17 | 17 | |
| Oryzias_latipes.9.23337195.23337830 | Oryzias_latipes.9.23337195.23337830 | 19 | 19 | |
| Oryzias_latipes.9.28835081.28835244 | Oryzias_latipes.9.28835081.28835244 | 22 | 22 | |
| Oryzias_latipes.9.2899473.2900962 | Oryzias_latipes.9.2899473.2900962 | 20 | 20 | |
| Oryzias_latipes.9.510018.510221 | Oryzias_latipes.9.510018.510221 | 18 | 18 | |
| Oryzias_latipes.9.9039709.9039257 | Oryzias_latipes.9.9039709.9039257 | 34 | 34 | |
| Oryzias_latipes.scaffold1376.15570.15771 | Oryzias_latipes.scaffold1376.15570.15771 | 10 | 10 | |
| Oryzias_latipes.scaffold2378.12948.12787 | Oryzias_latipes.scaffold2378.12948.12787 | 20 | 20 | |
| Oryzias_latipes.scaffold604.113603.113853 | Oryzias_latipes.scaffold604.113603.113853 | 7 | 30 | multiple blast hit in proteome: Alepocephalus_bicolor,Alepocephalus_brevipinnitm66merge,Anguilla_anguillatm125,Arctozensus_risso,Argentina_sphyraena029tm,Astronesthes_luciferT04tm,Borostomias_antarcticustm762,Coregonus_spZSM13380,Dallia_pectoralisUW150693-5,Danio_rerioD15,Evermanella_balbotm67eve,Galaxias_fasciatusCL282,Gonorhynchus_abbreviatus098tm,Hime_formosana137tm,Limnothrissa_miodon,Maurolicus_muelleritm2013W,Opisthoproctus_soleatusBPS0624,Pellonula_leonensistm238,Retropinna_tasmanica_CSIROtr,Rutilus_rutilus2,Rutilus_rutilustm70,Salmo_truttatm20154,Umbra_krameritm62 |

| | | | | |
|---|---|---|---|---|
| Oryzias_latipes.scaffold860.78177.79007 | Oryzias_latipes.scaffold860.78177.79007 | 18 | 18 | |
| Oryzias_latipes.scaffold869.51442.51606 | Oryzias_latipes.scaffold869.51442.51606 | 37 | 37 | |
| Oryzias_latipes.scaffold960.21590.21428 | Oryzias_latipes.scaffold960.21590.21428 | 35 | 35 | |
| Oryzias_latipes.ultracontig182.2208146.2207968 | Oryzias_latipes.ultracontig182.2208146.22 07968 | 14 | 14 | |
| Oryzias_latipes.ultracontig236.1451066.1450863 | Oryzias_latipes.ultracontig236.1451066.14 50863 | 10 | 10 | |
| Oryzias_latipes.ultracontig90.1005190.1005405 | Oryzias_latipes.ultracontig90.1005190.100 5405 | 30 | 30 | |
| Tetraodon_nigroviridis.1.10737224.10736912 | Tetraodon_nigroviridis.1.10737224.10736 912 | 19 | 19 | |
| Tetraodon_nigroviridis.1.10950272.10950097 | Tetraodon_nigroviridis.1.10950272.10950 097 | 27 | 27 | |
| Tetraodon_nigroviridis.1.10950974.10950784 | Tetraodon_nigroviridis.1.10950974.10950 784 | 16 | 16 | |
| Tetraodon_nigroviridis.1.1137430.1137269 | Tetraodon_nigroviridis.1.1137430.113726 9 | 13 | 13 | |
| Tetraodon_nigroviridis.1.11645919.11645671 | Tetraodon_nigroviridis.1.11645919.11645 671 | 26 | 26 | |
| Tetraodon_nigroviridis.1.11923866.11924049 | Tetraodon_nigroviridis.1.11923866.11924 049 | 33 | 33 | |
| Tetraodon_nigroviridis.1.12164607.12164454 | Tetraodon_nigroviridis.1.12164607.12164 454 | 21 | 21 | |
| Tetraodon_nigroviridis.1.14898664.14898945 | Tetraodon_nigroviridis.1.14898664.14898 945 | 19 | 19 | |
| Tetraodon_nigroviridis.1.14918943.14919171 | Tetraodon_nigroviridis.1.14918943.14919 171 | 15 | 15 | |
| Tetraodon_nigroviridis.1.15645428.15645231 | Tetraodon_nigroviridis.1.15645428.15645 231 | 32 | 32 | |
| Tetraodon_nigroviridis.1.15793367.15793639 | Tetraodon_nigroviridis.1.15793367.15793 639 | 29 | 29 | |
| Tetraodon_nigroviridis.1.4338542.4338845 | Tetraodon_nigroviridis.1.4338542.433884 5 | 13 | 13 | |

| | | | | |
|---|---|---|---|---|
| Tetraodon_nigroviridis.1.7217684.7217379 | Tetraodon_nigroviridis.1.7217684.7217379 | 37 | 37 | |
| Tetraodon_nigroviridis.1.8160511.8160890 | Tetraodon_nigroviridis.1.8160511.8160890 | 27 | 40 | multiple blast hit in proteome: Coregonus_spZSM13380,Dallia_pectoralisUW150693-5,Denticeps_clupeoidestm138,Engraulis_encrassicolustm57,Harpadon_microchir067tm,Hime_formosana137tm,Ijimaia_loppeiBPS2503,Lovettia_sealii_CSIROtr,Mallotus_villosustm016,Osmerus_eperlanustm785,Pecoglossus_altivelistm211,Retropinna_tasmanicaCL284a,Schilbe_mystustm551b |
| Tetraodon_nigroviridis.10.10552375.10552186 | Tetraodon_nigroviridis.10.10552375.10552186 | 27 | 27 | |
| Tetraodon_nigroviridis.10.12724961.12724768 | Tetraodon_nigroviridis.10.12724961.12724768 | 30 | 30 | |
| Tetraodon_nigroviridis.10.12726251.12726067 | Tetraodon_nigroviridis.10.12726251.12726067 | 28 | 28 | |
| Tetraodon_nigroviridis.10.1908663.1908283 | Tetraodon_nigroviridis.10.1908663.1908283 | 24 | 24 | |
| Tetraodon_nigroviridis.10.2492814.2492579 | Tetraodon_nigroviridis.10.2492814.2492579 | 29 | 29 | |
| Tetraodon_nigroviridis.10.4993447.4993203 | Tetraodon_nigroviridis.10.4993447.4993203 | 21 | 21 | |
| Tetraodon_nigroviridis.10.5837504.5837191 | Tetraodon_nigroviridis.10.5837504.5837191 | 24 | 24 | |
| Tetraodon_nigroviridis.10.606531.606275 | Tetraodon_nigroviridis.10.606531.606275 | 20 | 20 | |
| Tetraodon_nigroviridis.10.7818671.7818886 | Tetraodon_nigroviridis.10.7818671.7818886 | 40 | 40 | |
| Tetraodon_nigroviridis.10.9714335.9714123 | Tetraodon_nigroviridis.10.9714335.9714123 | 33 | 33 | |
| Tetraodon_nigroviridis.11.11125510.11125703 | Tetraodon_nigroviridis.11.11125510.11125703 | 16 | 16 | |
| Tetraodon_nigroviridis.11.2083785.2083983 | Tetraodon_nigroviridis.11.2083785.2083983 | 20 | 20 | |

| | | | | |
|---|---|---|---|---|
| Tetraodon_nigroviridis.11.2323116.2322893 | Tetraodon_nigroviridis.11.2323116.2322893 | 24 | 24 | |
| Tetraodon_nigroviridis.11.3046273.3046090 | Tetraodon_nigroviridis.11.3046273.3046090 | 27 | 27 | |
| Tetraodon_nigroviridis.11.6042394.6042193 | Tetraodon_nigroviridis.11.6042394.6042193 | 34 | 37 | multiple blast hit in proteome: Retropinna_tasmanicaCL284a,Retropinna_tasmanica_CSIROtr,Salanx_acuticepstmT02 |
| Tetraodon_nigroviridis.12.10805059.10805365 | Tetraodon_nigroviridis.12.10805059.10805365 | 30 | 30 | |
| Tetraodon_nigroviridis.12.10807658.10807874 | Tetraodon_nigroviridis.12.10807658.10807874 | 39 | 39 | |
| Tetraodon_nigroviridis.12.11834359.11834181 | Tetraodon_nigroviridis.12.11834359.11834181 | 37 | 37 | |
| Tetraodon_nigroviridis.12.2784042.2783890 | Tetraodon_nigroviridis.12.2784042.2783890 | 33 | 33 | |
| Tetraodon_nigroviridis.12.624348.624505 | Tetraodon_nigroviridis.12.624348.624505 | 23 | 23 | |
| Tetraodon_nigroviridis.12.6958819.6958526 | Tetraodon_nigroviridis.12.6958819.6958526 | 20 | 20 | |
| Tetraodon_nigroviridis.13.12428179.12428358 | Tetraodon_nigroviridis.13.12428179.12428358 | 1 | 23 | multiple blast hit in proteome: Alepocephalus_bicolor,Alepocephalus_brevipinnitm66merge,Argentina_sphyraena029tm,Cromeria_occidentalistm583a,Engraulis_encrassicolustm57,Evermanella_balbotm67eve,Galaxias_fasciatusCL282,Gonorhynchus_abbreviatus098tm,Ijimaia_loppeiBPS2503,Lepidogalaxias_salamandroidesSAM,Limnothrissa_miodon,Lovettia_sealii_CSIROtr,Mallotus_villosustm016,Opisthoproctus_soleatusBPS0624,Pantodon_bucholzi382tm,Pellonula_leonensistm238,Retropinna_tasmanicaCL284a, Retropinna_tasmanica_CSIROtr,Salanx_acuticepstmT02,Synodus_saurussn10,Umbra_krameritm62,bait_sequences |
| Tetraodon_nigroviridis.13.5229998.5230908 | Tetraodon_nigroviridis.13.5229998.5230908 | 16 | 16 | |

| | | | | |
|---|---|---|---|---|
| Tetraodon_nigroviridis.13.5969653.5969898 | Tetraodon_nigroviridis.13.5969653.5969898 | 24 | 24 | |
| Tetraodon_nigroviridis.13.6490366.6490193 | Tetraodon_nigroviridis.13.6490366.6490193 | 13 | 13 | |
| Tetraodon_nigroviridis.13.7229990.7230145 | Tetraodon_nigroviridis.13.7229990.7230145 | 37 | 38 | too short: Umbra_krameritm62 |
| Tetraodon_nigroviridis.13.7895200.7894972 | Tetraodon_nigroviridis.13.7895200.7894972 | 28 | 28 | |
| Tetraodon_nigroviridis.14.2733367.2733158 | Tetraodon_nigroviridis.14.2733367.2733158 | 38 | 38 | |
| Tetraodon_nigroviridis.14.3282493.3282323 | Tetraodon_nigroviridis.14.3282493.3282323 | 13 | 13 | |
| Tetraodon_nigroviridis.14.3743844.3743352 | Tetraodon_nigroviridis.14.3743844.3743352 | 19 | 20 | too short: Arctozensus_risso |
| Tetraodon_nigroviridis.14.4997368.4997616 | Tetraodon_nigroviridis.14.4997368.4997616 | 13 | 13 | |
| Tetraodon_nigroviridis.14.5003764.5004693 | Tetraodon_nigroviridis.14.5003764.5004693 | 29 | 29 | |
| Tetraodon_nigroviridis.14.6014338.6014173 | Tetraodon_nigroviridis.14.6014338.6014173 | 36 | 36 | |
| Tetraodon_nigroviridis.14.6747216.6746840 | Tetraodon_nigroviridis.14.6747216.6746840 | 31 | 31 | |
| Tetraodon_nigroviridis.14.6769493.6769699 | Tetraodon_nigroviridis.14.6769493.6769699 | 14 | 14 | |
| Tetraodon_nigroviridis.14.9520621.9520376 | Tetraodon_nigroviridis.14.9520621.9520376 | 25 | 25 | |
| Tetraodon_nigroviridis.14.9531497.9531711 | Tetraodon_nigroviridis.14.9531497.9531711 | 13 | 13 | |
| Tetraodon_nigroviridis.15.2856796.2856985 | Tetraodon_nigroviridis.15.2856796.2856985 | 15 | 15 | |
| Tetraodon_nigroviridis.16.1199095.1199265 | Tetraodon_nigroviridis.16.1199095.1199265 | 32 | 32 | |
| Tetraodon_nigroviridis.16.3516613.3516028 | Tetraodon_nigroviridis.16.3516613.3516028 | 23 | 24 | too short: Danio_rerioD15 |
| Tetraodon_nigroviridis.16.3836165.3836462 | Tetraodon_nigroviridis.16.3836165.3836462 | 11 | 11 | |

| | | | | |
|---|---|---|---|---|
| Tetraodon_nigroviridis.16.3836777.3836983 | Tetraodon_nigroviridis.16.3836777.3836983 | 24 | 24 | |
| Tetraodon_nigroviridis.16.4027481.4026885 | Tetraodon_nigroviridis.16.4027481.4026885 | 21 | 21 | |
| Tetraodon_nigroviridis.17.12048904.12049087 | Tetraodon_nigroviridis.17.12048904.12049087 | 21 | 21 | |
| Tetraodon_nigroviridis.17.4091888.4091700 | Tetraodon_nigroviridis.17.4091888.4091700 | 38 | 38 | |
| Tetraodon_nigroviridis.17.5587047.5587238 | Tetraodon_nigroviridis.17.5587047.5587238 | 35 | 35 | |
| Tetraodon_nigroviridis.17.6022906.6022656 | Tetraodon_nigroviridis.17.6022906.6022656 | 31 | 31 | |
| Tetraodon_nigroviridis.17.6600380.6600160 | Tetraodon_nigroviridis.17.6600380.6600160 | 30 | 30 | |
| Tetraodon_nigroviridis.18.10815524.10815717 | Tetraodon_nigroviridis.18.10815524.10815717 | 9 | 31 | multiple blast hit in proteome: Alepocephalus_brevipinnitm66merge,Alestes_baremozetm285,Argentina_sphyraena029tm,Astronesthes_luciferT04tm,Bathylagus_euryopstm112,Borostomias_antarcticustm762,Coregonus_spZSM13380,Cromeria_occidentalistm583a,Danio_rerioD15,Denticeps_clupeoidestm138,Dussumiera_elopsoides037tm,Gonorhynchus_abbreviatus098tm,Gymnotus_spZSM32021,Ijimaia_loppeiBPS2503,Limnothrissa_miodon,Opisthoproctus_soleatusBPS0624,Retropinna_tasmanica_CSIROtr,Rutilus_rutilustm70,Salanx_acuticepstmT02,Salmo_truttatm20154,Synodus_saurustm67syneve,bait_sequences |
| Tetraodon_nigroviridis.18.2451038.2450849 | Tetraodon_nigroviridis.18.2451038.2450849 | 24 | 24 | |
| Tetraodon_nigroviridis.18.274610.273545 | Tetraodon_nigroviridis.18.274610.273545 | 25 | 25 | |
| Tetraodon_nigroviridis.18.2823185.2823376 | Tetraodon_nigroviridis.18.2823185.2823376 | 28 | 28 | |
| Tetraodon_nigroviridis.18.2856457.2856631 | Tetraodon_nigroviridis.18.2856457.2856631 | 24 | 24 | |

| | | | | |
|---|---|---|---|---|
| Tetraodon_nigroviridis.18.3870739.3870548 | Tetraodon_nigroviridis.18.3870739.3870548 | 17 | 17 | |
| Tetraodon_nigroviridis.18.4211448.4211215 | Tetraodon_nigroviridis.18.4211448.4211215 | 26 | 26 | |
| Tetraodon_nigroviridis.18.9142290.9142066 | Tetraodon_nigroviridis.18.9142290.9142066 | 13 | 13 | |
| Tetraodon_nigroviridis.19.1646077.1646302 | Tetraodon_nigroviridis.19.1646077.1646302 | 33 | 33 | |
| Tetraodon_nigroviridis.19.2032318.2032148 | Tetraodon_nigroviridis.19.2032318.2032148 | 29 | 29 | |
| Tetraodon_nigroviridis.19.2225567.2225724 | Tetraodon_nigroviridis.19.2225567.2225724 | 25 | 25 | |
| Tetraodon_nigroviridis.19.2487255.2487052 | Tetraodon_nigroviridis.19.2487255.2487052 | 25 | 25 | |
| Tetraodon_nigroviridis.19.3422239.3422020 | Tetraodon_nigroviridis.19.3422239.3422020 | 33 | 33 | |
| Tetraodon_nigroviridis.19.561266.561497 | Tetraodon_nigroviridis.19.561266.561497 | 32 | 32 | |
| Tetraodon_nigroviridis.2.10526544.10526320 | Tetraodon_nigroviridis.2.10526544.10526320 | 32 | 32 | |
| Tetraodon_nigroviridis.2.10541460.10541254 | Tetraodon_nigroviridis.2.10541460.10541254 | 28 | 28 | |
| Tetraodon_nigroviridis.2.10549078.10548874 | Tetraodon_nigroviridis.2.10549078.10548874 | 28 | 28 | |
| Tetraodon_nigroviridis.2.10555584.10555342 | Tetraodon_nigroviridis.2.10555584.10555342 | 30 | 30 | |
| Tetraodon_nigroviridis.2.1170368.1170619 | Tetraodon_nigroviridis.2.1170368.1170619 | 26 | 26 | |
| Tetraodon_nigroviridis.2.15425986.15426315 | Tetraodon_nigroviridis.2.15425986.15426315 | 30 | 30 | |
| Tetraodon_nigroviridis.2.19609152.19609367 | Tetraodon_nigroviridis.2.19609152.19609367 | 12 | 12 | |
| Tetraodon_nigroviridis.2.20016378.20016552 | Tetraodon_nigroviridis.2.20016378.20016552 | 26 | 26 | |
| Tetraodon_nigroviridis.2.20016623.20016799 | Tetraodon_nigroviridis.2.20016623.20016799 | 33 | 33 | |

| | | | | |
|---|---|---|---|---|
| Tetraodon_nigroviridis.2.2344868.2344708 | Tetraodon_nigroviridis.2.2344868.2344708 | 27 | 27 | |
| Tetraodon_nigroviridis.2.5819018.5819225 | Tetraodon_nigroviridis.2.5819018.5819225 | 20 | 20 | |
| Tetraodon_nigroviridis.2.6840789.6840614 | Tetraodon_nigroviridis.2.6840789.6840614 | 35 | 35 | |
| Tetraodon_nigroviridis.2.8835022.8834718 | Tetraodon_nigroviridis.2.8835022.8834718 | 20 | 20 | |
| Tetraodon_nigroviridis.2.9375060.9374864 | Tetraodon_nigroviridis.2.9375060.9374864 | 36 | 36 | |
| Tetraodon_nigroviridis.2.9862405.9862088 | Tetraodon_nigroviridis.2.9862405.9862088 | 1 | 21 | multiple blast hit in proteome: Alepocephalus_bicolor,Alepocephalus_brevipinnitm66merge,Alestes_baremozetm285,Arctozensus_risso,Argentina_sphyraena029tm,Ateleopus_japonicus133tm,Bathylagus_euryopstm112,Coregonus_spZSM13380,Evermanella_balbotm67eve,Galaxias_fasciatusCL282,Gonorhynchus_abbreviatus098tm,Harpadon_microchir067tm,Hime_formosana137tm,Ijimaia_loppeiBPS2503,Lestrolepis_japonicustmT03,Lovettia_sealii_CSIROtr,Nansenia_oblitaBPS1997,Rutilus_rutilus2,Synodus_saurussn10,bait_sequences |
| Tetraodon_nigroviridis.20.506247.506095 | Tetraodon_nigroviridis.20.506247.506095 | 30 | 30 | |
| Tetraodon_nigroviridis.20.569620.569802 | Tetraodon_nigroviridis.20.569620.569802 | 34 | 34 | |
| Tetraodon_nigroviridis.21.4521965.4522122 | Tetraodon_nigroviridis.21.4521965.4522122 | 25 | 25 | |
| Tetraodon_nigroviridis.21.4849258.4848375 | Tetraodon_nigroviridis.21.4849258.4848375 | 30 | 30 | |
| Tetraodon_nigroviridis.21.5083783.5084055 | Tetraodon_nigroviridis.21.5083783.5084055 | 11 | 11 | |
| Tetraodon_nigroviridis.21.5512171.5512383 | Tetraodon_nigroviridis.21.5512171.5512383 | 25 | 25 | |
| Tetraodon_nigroviridis.21.743323.743146 | Tetraodon_nigroviridis.21.743323.743146 | 13 | 13 | |

| | | | | |
|---|---|---|---|---|
| Tetraodon_nigroviridis.21_random.399167.399322 | Tetraodon_nigroviridis.21_random.399167.399322 | 27 | 27 | |
| Tetraodon_nigroviridis.3.11217920.11217764 | Tetraodon_nigroviridis.3.11217920.11217764 | 14 | 14 | |
| Tetraodon_nigroviridis.3.11591528.11591827 | Tetraodon_nigroviridis.3.11591528.11591827 | 24 | 24 | |
| Tetraodon_nigroviridis.3.12406720.12407208 | Tetraodon_nigroviridis.3.12406720.12407208 | 24 | 24 | |
| Tetraodon_nigroviridis.3.12541672.12541505 | Tetraodon_nigroviridis.3.12541672.12541505 | 20 | 20 | |
| Tetraodon_nigroviridis.3.12542113.12541934 | Tetraodon_nigroviridis.3.12542113.12541934 | 20 | 20 | |
| Tetraodon_nigroviridis.3.1537072.1536461 | Tetraodon_nigroviridis.3.1537072.1536461 | 42 | 42 | |
| Tetraodon_nigroviridis.3.8678081.8677902 | Tetraodon_nigroviridis.3.8678081.8677902 | 25 | 25 | |
| Tetraodon_nigroviridis.3.8980133.8979949 | Tetraodon_nigroviridis.3.8980133.8979949 | 30 | 31 | multiple blast hit in proteome: Galaxias_fasciatusCL282 |
| Tetraodon_nigroviridis.3.9460089.9460559 | Tetraodon_nigroviridis.3.9460089.9460559 | 32 | 32 | |
| Tetraodon_nigroviridis.4.1173303.1173135 | Tetraodon_nigroviridis.4.1173303.1173135 | 22 | 22 | |
| Tetraodon_nigroviridis.4.6802443.6802612 | Tetraodon_nigroviridis.4.6802443.6802612 | 16 | 16 | |
| Tetraodon_nigroviridis.4.6908907.6908735 | Tetraodon_nigroviridis.4.6908907.6908735 | 31 | 31 | |
| Tetraodon_nigroviridis.5.1284444.1284729 | Tetraodon_nigroviridis.5.1284444.1284729 | 36 | 36 | |
| Tetraodon_nigroviridis.5.5093556.5093763 | Tetraodon_nigroviridis.5.5093556.5093763 | 15 | 15 | |
| Tetraodon_nigroviridis.5.5095363.5095560 | Tetraodon_nigroviridis.5.5095363.5095560 | 34 | 34 | |
| Tetraodon_nigroviridis.5.5362815.5362598 | Tetraodon_nigroviridis.5.5362815.5362598 | 41 | 41 | |
| Tetraodon_nigroviridis.5.7845285.7845440 | Tetraodon_nigroviridis.5.7845285.7845440 | 28 | 28 | |

| | | | | |
|---|---|---|---|---|
| Tetraodon_nigroviridis.6.2366076.2365884 | Tetraodon_nigroviridis.6.2366076.2365884 | 39 | 39 | |
| Tetraodon_nigroviridis.7.1869628.1869444 | Tetraodon_nigroviridis.7.1869628.1869444 | 24 | 24 | |
| Tetraodon_nigroviridis.7.2774803.2774609 | Tetraodon_nigroviridis.7.2774803.2774609 | 13 | 13 | |
| Tetraodon_nigroviridis.7.6053517.6053242 | Tetraodon_nigroviridis.7.6053517.6053242 | 30 | 30 | |
| Tetraodon_nigroviridis.7.9865920.9865748 | Tetraodon_nigroviridis.7.9865920.9865748 | 31 | 31 | |
| Tetraodon_nigroviridis.8.10176215.10176396 | Tetraodon_nigroviridis.8.10176215.10176396 | 17 | 17 | |
| Tetraodon_nigroviridis.8.2413296.2413502 | Tetraodon_nigroviridis.8.2413296.2413502 | 22 | 22 | |
| Tetraodon_nigroviridis.8.7008945.7009118 | Tetraodon_nigroviridis.8.7008945.7009118 | 14 | 14 | |
| Tetraodon_nigroviridis.8.7731385.7731559 | Tetraodon_nigroviridis.8.7731385.7731559 | 24 | 24 | |
| Tetraodon_nigroviridis.8.9124044.9124240 | Tetraodon_nigroviridis.8.9124044.9124240 | 22 | 22 | |
| Tetraodon_nigroviridis.9.10434476.10434252 | Tetraodon_nigroviridis.9.10434476.10434252 | 18 | 18 | |
| Tetraodon_nigroviridis.9.1221010.1220801 | Tetraodon_nigroviridis.9.1221010.1220801 | 36 | 36 | |
| Tetraodon_nigroviridis.9.1221943.1221775 | Tetraodon_nigroviridis.9.1221943.1221775 | 20 | 20 | |
| Tetraodon_nigroviridis.9.3077427.3077242 | Tetraodon_nigroviridis.9.3077427.3077242 | 29 | 29 | |
| Tetraodon_nigroviridis.9.3584659.3584877 | Tetraodon_nigroviridis.9.3584659.3584877 | 23 | 23 | |
| Tetraodon_nigroviridis.9.3588928.3589197 | Tetraodon_nigroviridis.9.3588928.3589197 | 23 | 23 | |
| Tetraodon_nigroviridis.9.5241075.5240907 | Tetraodon_nigroviridis.9.5241075.5240907 | 21 | 21 | |
| Tetraodon_nigroviridis.9.5723174.5722998 | Tetraodon_nigroviridis.9.5723174.5722998 | 40 | 40 | |

| | | | | |
|---|---|---|---|---|
| Tetraodon_nigroviridis.Un_random.12774827.12774582 | Tetraodon_nigroviridis.Un_random.12774827.12774582 | 28 | 28 | |
| Tetraodon_nigroviridis.Un_random.13372107.13371920 | Tetraodon_nigroviridis.Un_random.13372107.13371920 | 32 | 32 | |
| Tetraodon_nigroviridis.Un_random.16051748.16051943 | Tetraodon_nigroviridis.Un_random.16051748.16051943 | 32 | 32 | |
| Tetraodon_nigroviridis.Un_random.16158015.16158174 | Tetraodon_nigroviridis.Un_random.16158015.16158174 | 20 | 20 | |
| Tetraodon_nigroviridis.Un_random.19675650.19675847 | Tetraodon_nigroviridis.Un_random.19675650.19675847 | 41 | 41 | |
| Tetraodon_nigroviridis.Un_random.21080959.21081207 | Tetraodon_nigroviridis.Un_random.21080959.21081207 | 35 | 35 | |
| Tetraodon_nigroviridis.Un_random.22227359.22227187 | Tetraodon_nigroviridis.Un_random.22227359.22227187 | 27 | 27 | |
| Tetraodon_nigroviridis.Un_random.23009819.23009460 | Tetraodon_nigroviridis.Un_random.23009819.23009460 | 26 | 26 | |
| Tetraodon_nigroviridis.Un_random.27811605.27811794 | Tetraodon_nigroviridis.Un_random.27811605.27811794 | 31 | 31 | |
| Tetraodon_nigroviridis.Un_random.30449827.30449639 | Tetraodon_nigroviridis.Un_random.30449827.30449639 | 30 | 30 | |
| Tetraodon_nigroviridis.Un_random.30592448.30592657 | Tetraodon_nigroviridis.Un_random.30592448.30592657 | 27 | 27 | |
| Tetraodon_nigroviridis.Un_random.31223845.31224002 | Tetraodon_nigroviridis.Un_random.31223845.31224002 | 24 | 24 | |
| Tetraodon_nigroviridis.Un_random.33206035.33205750 | Tetraodon_nigroviridis.Un_random.33206035.33205750 | 19 | 19 | |
| Tetraodon_nigroviridis.Un_random.34922753.34922572 | Tetraodon_nigroviridis.Un_random.34922753.34922572 | 19 | 19 | |
| Tetraodon_nigroviridis.Un_random.35055718.35056321 | Tetraodon_nigroviridis.Un_random.35055718.35056321 | 25 | 25 | |
| Tetraodon_nigroviridis.Un_random.35061094.35062504 | Tetraodon_nigroviridis.Un_random.35061094.35062504 | 45 | 45 | |
| Tetraodon_nigroviridis.Un_random.35433468.35433272 | Tetraodon_nigroviridis.Un_random.35433468.35433272 | 33 | 33 | |
| Tetraodon_nigroviridis.Un_random.38209794.38209586 | Tetraodon_nigroviridis.Un_random.38209794.38209586 | 24 | 24 | |

| | | | | |
|---|---|---|---|---|
| Tetraodon_nigroviridis.Un_random.39526571.39526367 | Tetraodon_nigroviridis.Un_random.39526571.39526367 | 35 | 35 | |
| Tetraodon_nigroviridis.Un_random.44328644.44328808 | Tetraodon_nigroviridis.Un_random.44328644.44328808 | 24 | 24 | |
| Tetraodon_nigroviridis.Un_random.55926161.55926338 | Tetraodon_nigroviridis.Un_random.55926161.55926338 | 27 | 27 | |
| Tetraodon_nigroviridis.Un_random.56239230.56239406 | Tetraodon_nigroviridis.Un_random.56239230.56239406 | 21 | 21 | |
| Tetraodon_nigroviridis.Un_random.5801278.5801710 | Tetraodon_nigroviridis.Un_random.5801278.5801710 | 21 | 21 | |
| Tetraodon_nigroviridis.Un_random.65948553.65948341 | Tetraodon_nigroviridis.Un_random.65948553.65948341 | 43 | 43 | |
| Tetraodon_nigroviridis.Un_random.67565861.67565588 | Tetraodon_nigroviridis.Un_random.67565861.67565588 | 25 | 25 | |
| Tetraodon_nigroviridis.Un_random.67566514.67566276 | Tetraodon_nigroviridis.Un_random.67566514.67566276 | 30 | 30 | |
| Tetraodon_nigroviridis.Un_random.68091178.68091359 | Tetraodon_nigroviridis.Un_random.68091178.68091359 | 0 | 13 | multiple blast hit in proteome: Alepocephalus_brevipinnitm66merge,Alestes_baremozetm285,Argentina_sphyraena029tm,Coregonus_spZSM13380,Cromeria_occidentalistm583a,Danio_rerioD15,Denticeps_clupeoidestm138,Gonorhynchus_abbreviatus098tm,Lepidogalaxias_salamandroidesSAM,Lovettia_sealii_CSIROtr,Nansenia_oblitaBPS1997,Salmo_truttatm20154,bait_sequences |
| Tetraodon_nigroviridis.Un_random.71614613.71614823 | Tetraodon_nigroviridis.Un_random.71614613.71614823 | 11 | 11 | |
| Tetraodon_nigroviridis.Un_random.71759584.71759414 | Tetraodon_nigroviridis.Un_random.71759584.71759414 | 22 | 22 | |
| Tetraodon_nigroviridis.Un_random.72724049.72724201 | Tetraodon_nigroviridis.Un_random.72724049.72724201 | 42 | 42 | |
| Tetraodon_nigroviridis.Un_random.73107685.73107840 | Tetraodon_nigroviridis.Un_random.73107685.73107840 | 35 | 35 | |
| Tetraodon_nigroviridis.Un_random.78089505.78089667 | Tetraodon_nigroviridis.Un_random.78089505.78089667 | 33 | 33 | |

| | | | | |
|---|---|---|---|---|
| Tetraodon_nigroviridis.Un_random.7929823.7929618 | Tetraodon_nigroviridis.Un_random.7929823.7929618 | 26 | 26 | |
| Tetraodon_nigroviridis.Un_random.81044266.81044574 | Tetraodon_nigroviridis.Un_random.81044266.81044574 | 28 | 28 | |
| Tetraodon_nigroviridis.Un_random.86178802.86178608 | Tetraodon_nigroviridis.Un_random.86178802.86178608 | 19 | 19 | |
| Tetraodon_nigroviridis.Un_random.91569384.91569213 | Tetraodon_nigroviridis.Un_random.91569384.91569213 | 38 | 38 | |
| Tetraodon_nigroviridis.Un_random.96042125.96042295 | Tetraodon_nigroviridis.Un_random.96042125.96042295 | 38 | 38 | |

[1] Name of locus
[2] Name of orthology group. Sequences of orthology group come from the baits-designing sequences
[3] Number of samples have reciprocal blast hit in this locus
[4] Number of enriched samples in this locus

Analysis protocol for the Bayesian inference using Phylobayes 3.3f [43, 78, 79]:

**1. Concatenated amino acid dataset:**

bpcomp diff:

maxdiff : 0.101187

meandiff : 0.00188709

Tracecomp.contdiff:

| name | effsize | rel_diff |
|---|---|---|
| loglik | 1791 | 0.0366979 |
| length | 4101 | 0.00560435 |
| alpha | 2693 | 0.0808239 |
| Nmode | 1280 | 0.0324392 |
| statent | 1428 | 0.0611291 |
| statalpha | 1342 | 0.0143586 |

**2. Concatenated DNA dataset:**

bpcomp diff:

maxdiff : 0.0877491

meandiff : 0.00241954

Tracecomp.contdiff:

| name | effsize | rel_diff |
|---|---|---|
| loglik | 471 | 0.548605 |
| length | 409 | 0.274967 |
| alpha | 222 | 0.136775 |
| Nmode | 238 | 0.782935 |
| statent | 610 | 0.274705 |
| statalpha | 143 | 0.130307 |

Table S4: results from CONSEL AU test [42, 48, 49, 50, 51].

| rank | item | dataset description | obs | au | np | bp | pp | kh | sh | wkh | wsh |
|---|---|---|---|---|---|---|---|---|---|---|---|
| **1** | 6 | DNA_MARE_concatenated_partitioned_RAxML best tree | 0 | 0.776 | 0.55 | 0.567 | 0.5 | 0.71 | 0.946 | 0.71 | 0.946 |
| **2** | 5 | DNA_MARE_concatenated_non-partitioned_RAxML best tree | 0 | 0.398 | 0.241 | 0.225 | 0.5 | 0.29 | 0.863 | 0.29 | 0.863 |
| **3** | 1 | DNA_speciestree | 29.9 | 0.201 | 0.212 | 0.209 | 5.00E-14 | 0.215 | 0.48 | 0.215 | 0.383 |
| **4** | 2 | AA_speciestree | 210.1 | 9.00E-26 | 7.00E-12 | 0 | 3.00E-92 | 0 | 0 | 0 | 0 |
| **5** | 4 | AA_MARE_concatenated_non_partitioned_RAxML best tree | 416.4 | 6.00E-69 | 1.00E-19 | 0 | 7.00E-182 | 0 | 0 | 0 | 0 |
| **6** | 3 | AA_MARE_concatenated_partitioned_RAxML best tree | 416.4 | 2.00E-69 | 8.00E-20 | 0 | 7.00E-182 | 0 | 0 | 0 | 0 |

Fig. S1: RAxML Maximum Likelihhod estimate based on the concatenated amino acid alignments of 829 most informative loci identified by MARE. Data partinoned based on results from PartitionFinderProtein.

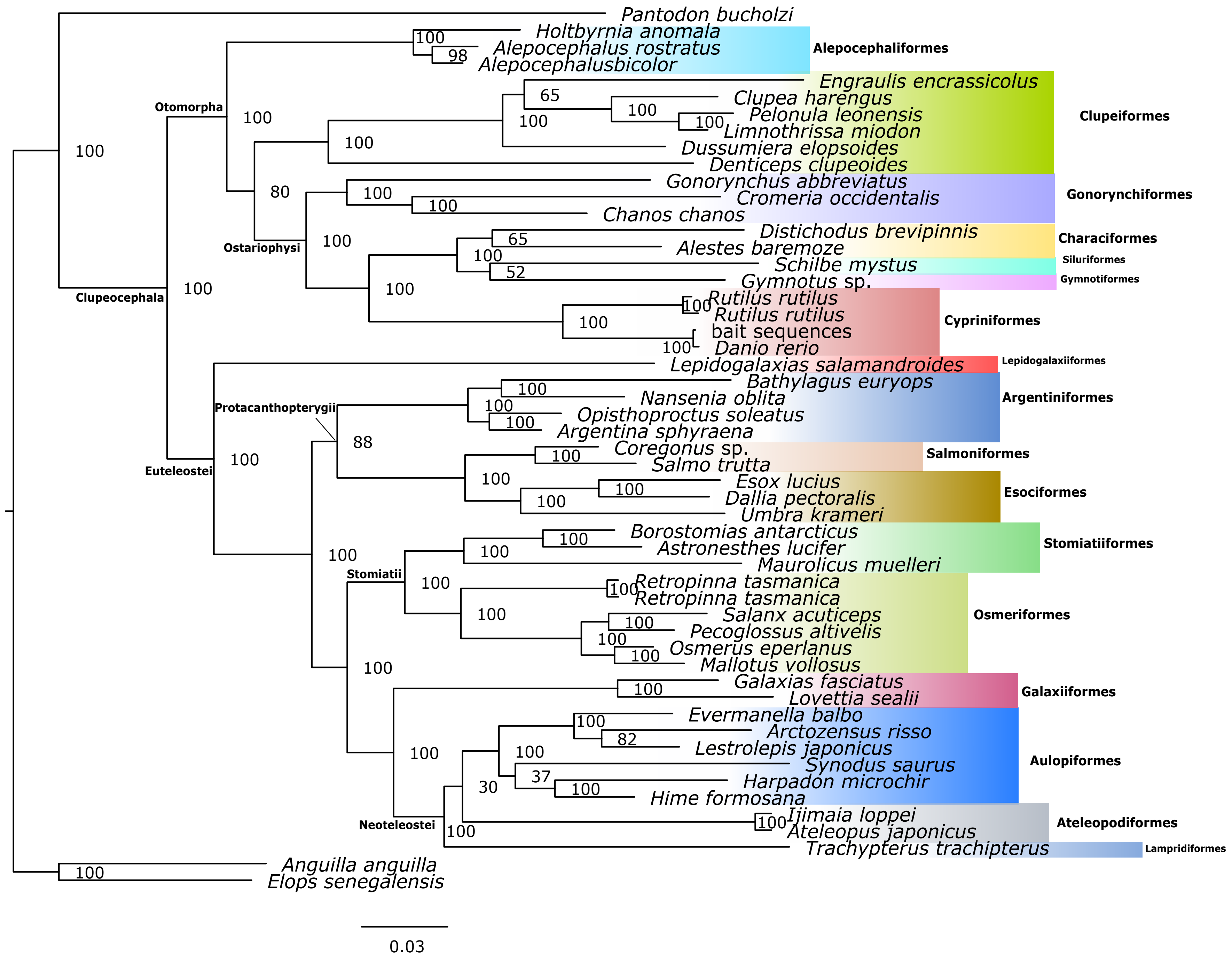

Fig. S2: RAxML Maximum Likelihhod estimate based on the
concatenated amino acid alignments of 829 most informative loci identified by MARE.
Data not partinoned.

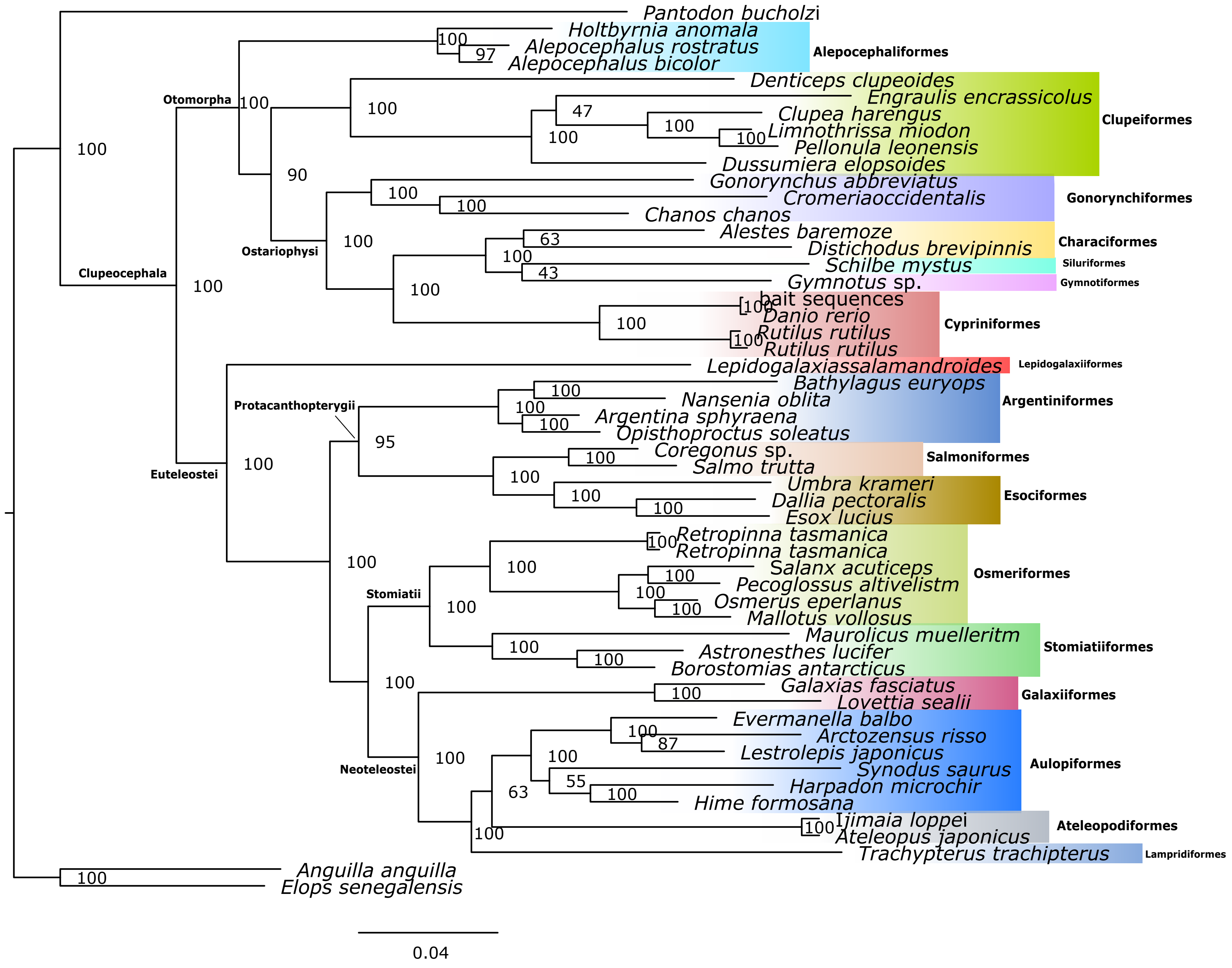

Fig. S3: RAxML Maximum Likelihhod estimate based on the concatenated nucleotide alignments of 829 most informative loci identified by MARE. Data partinoned based on results from PartitionFinder..

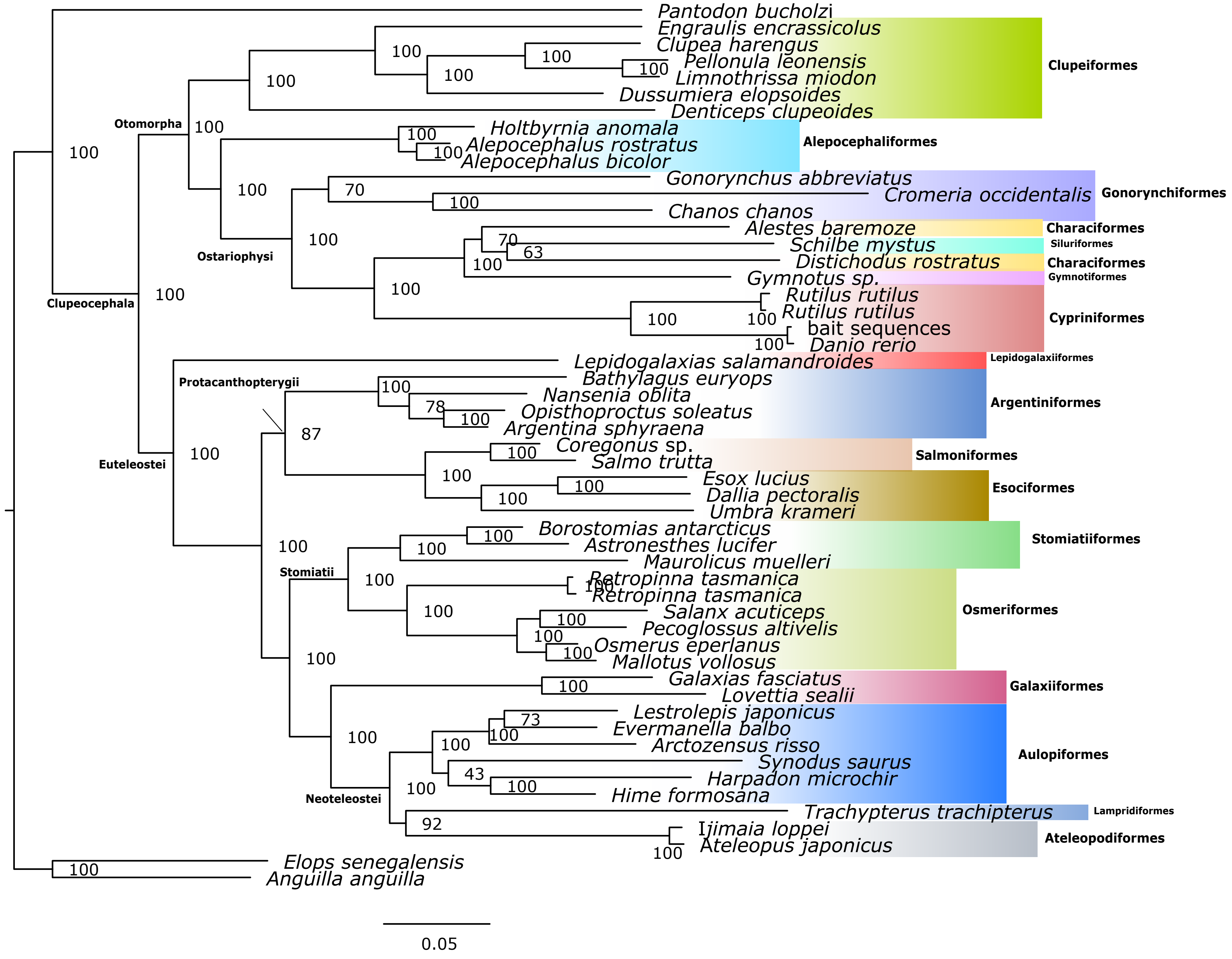

Fig. S4: RAxML Maximum Likelihood estimate based on the concatenated nucleotide alignments of 829 most informative loci identified by MARE. Data not partinoned.

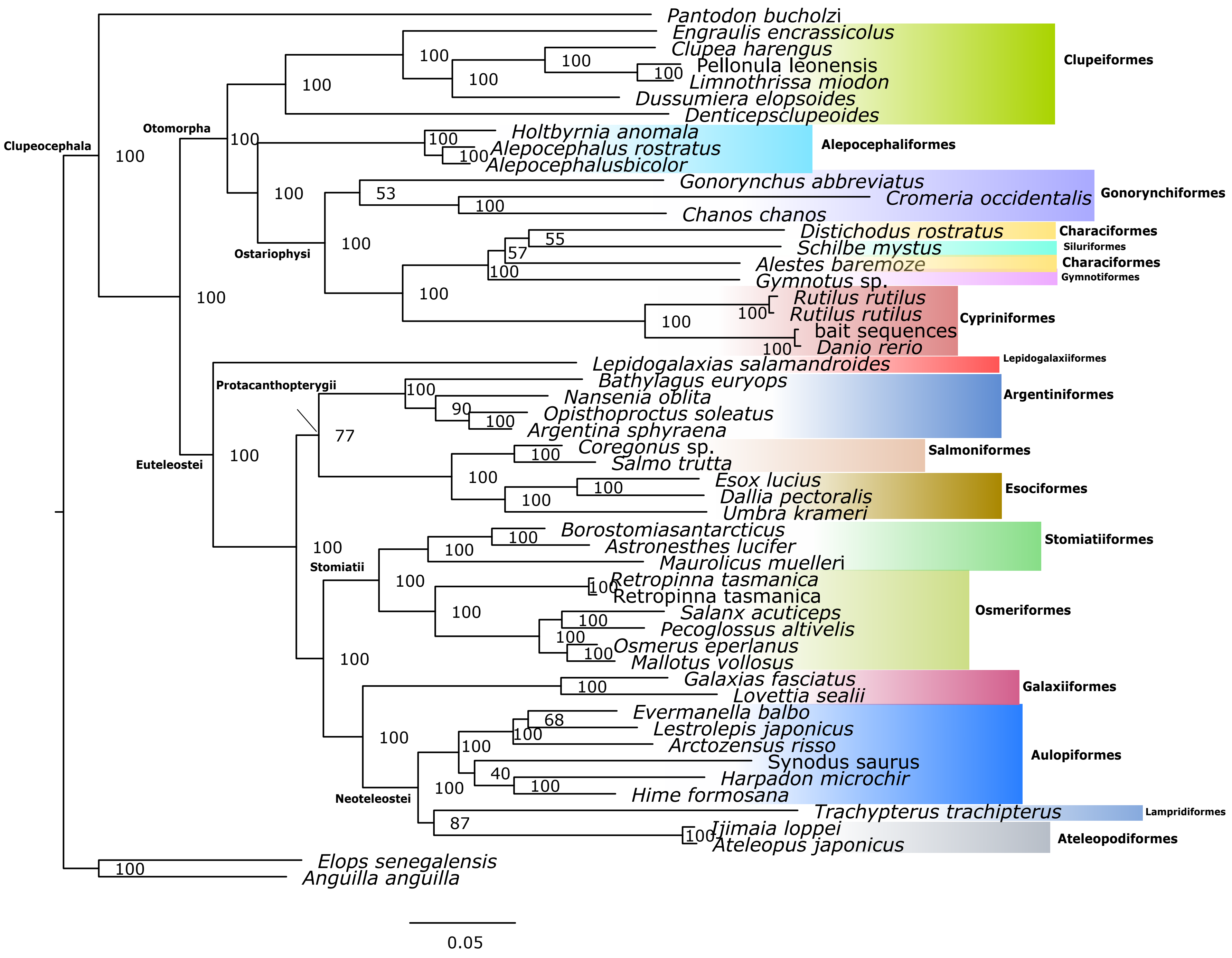

Fig. S5: Bayesian inference using the CAT Dirichlet process implemented in PhyloBayes on the concatenated amino acid alignments of 829 phylogenetically most informative loci identified with MARE.

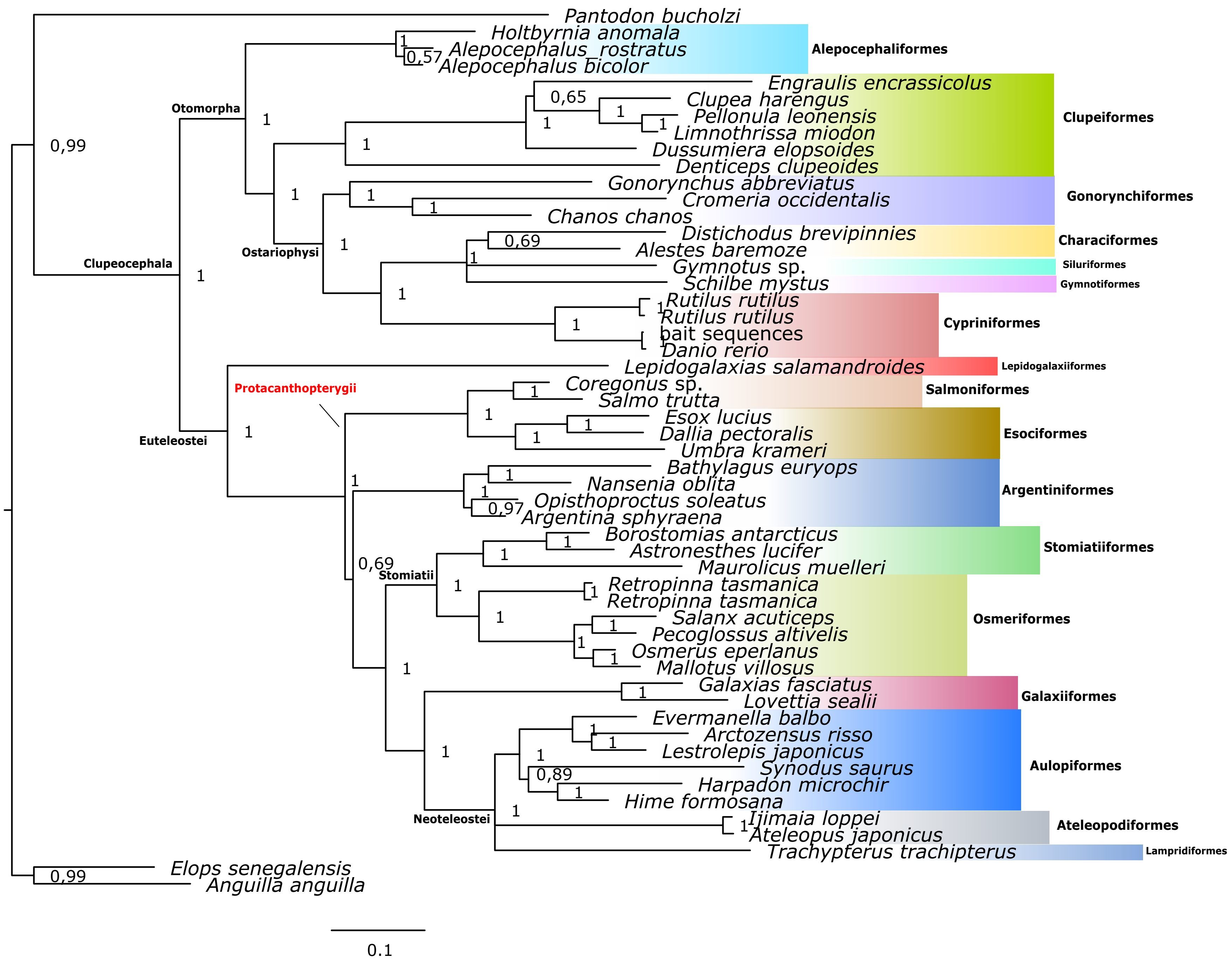

Fig. S6: Bayesian inference using the CAT Dirichlet process implemented in PhyloBayes on the concatenated nucleotide alignments of 829 phylogenetically most informative loci identified with MARE.

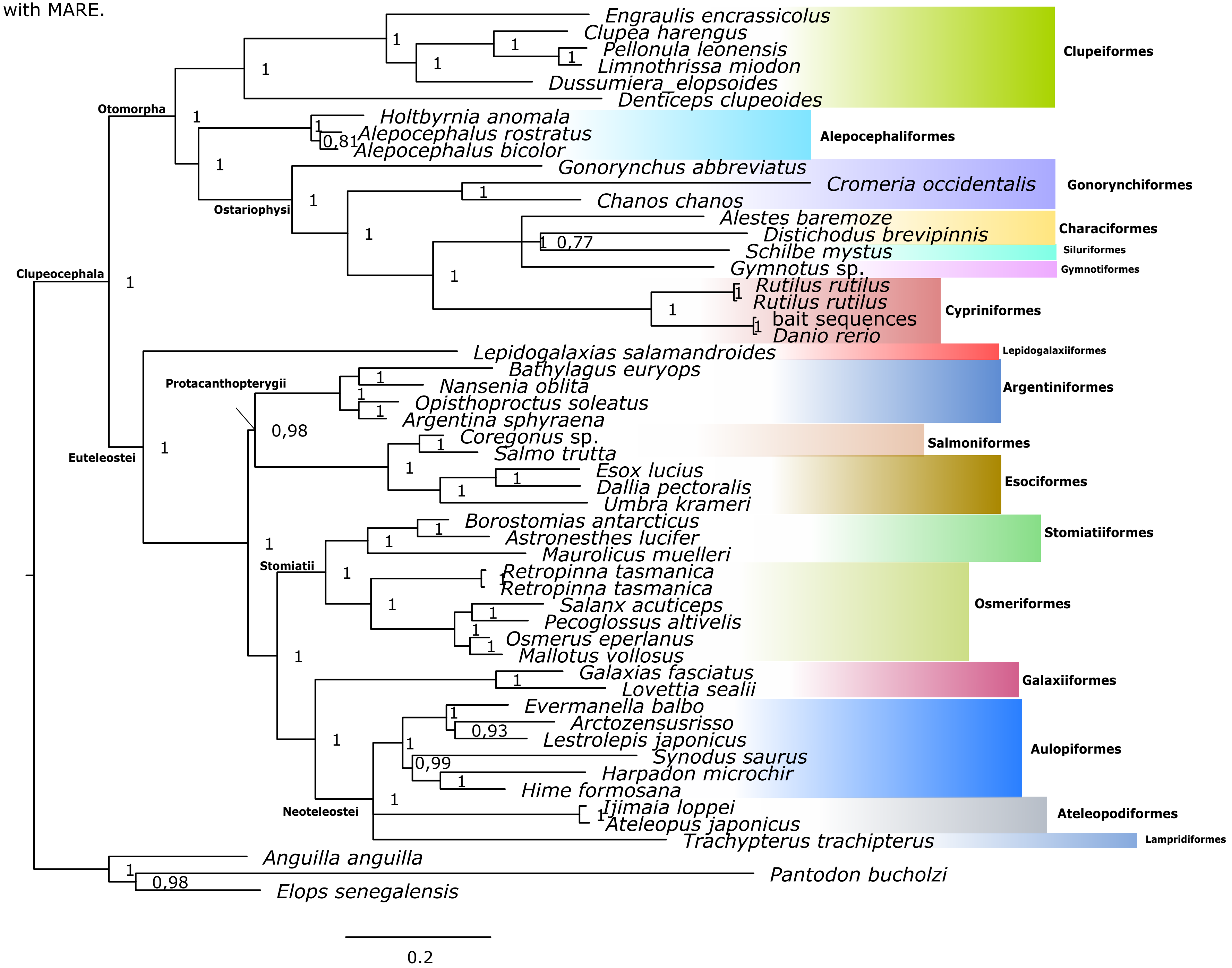

Fig. S7: Speciestree computed using ASTRAL from 829 individual maximum likelihood gene trees based on single loci amino acid alignments..

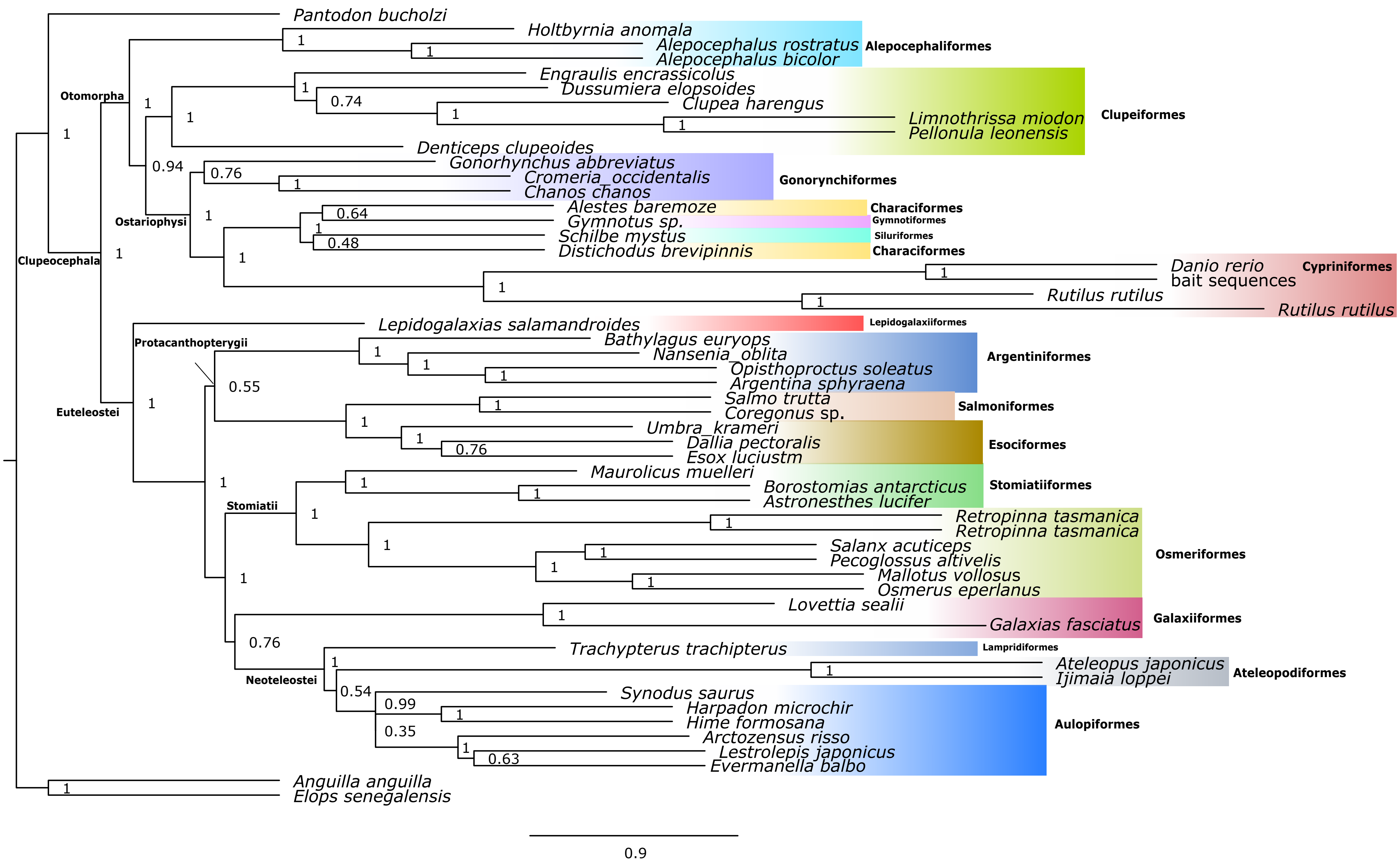

Fig. S8: Speciestree computed using ASTRAL from 829 individual maximum likelihood gene trees based on single loci DNA alignments.

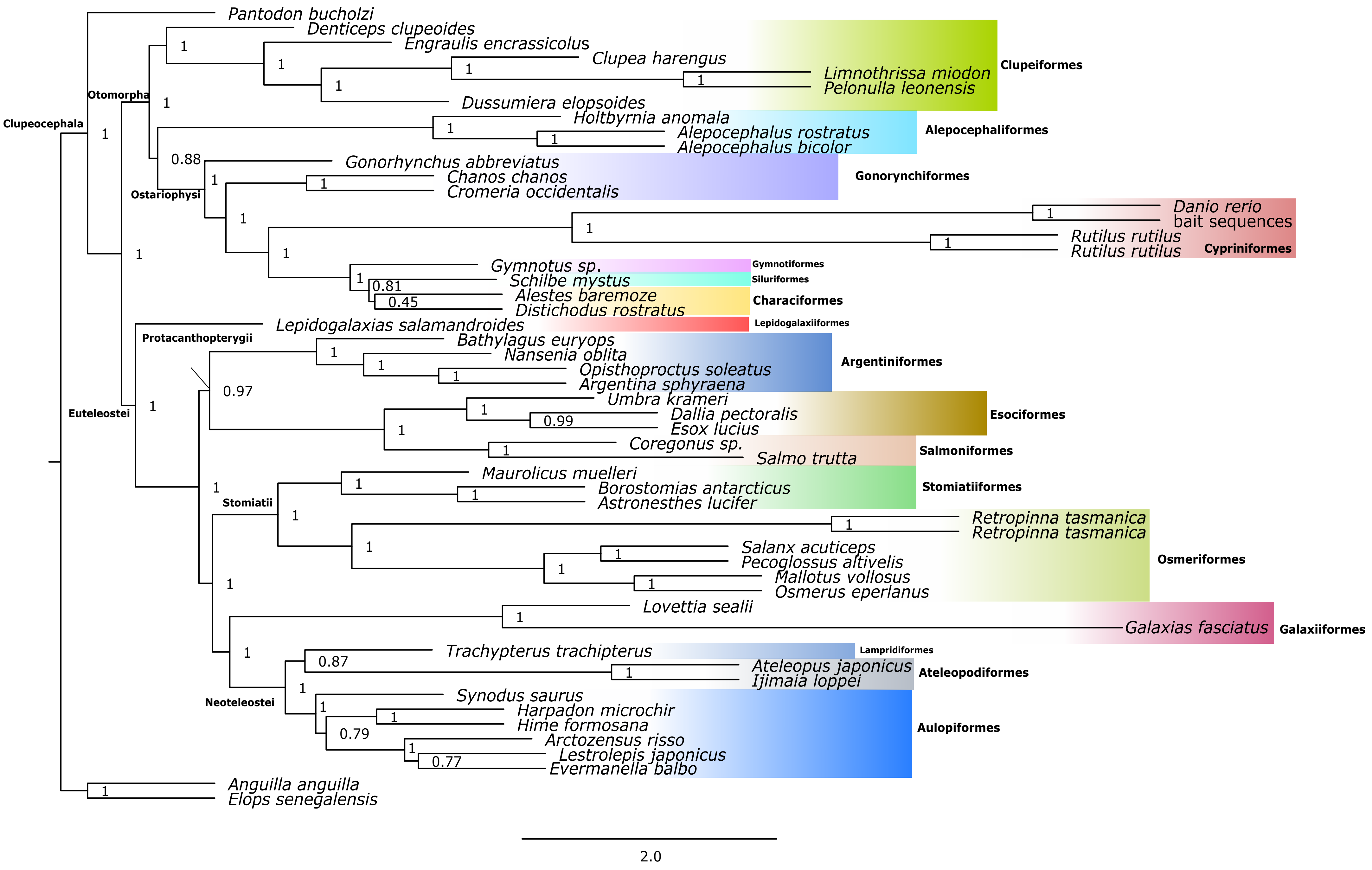

Supplement: Supplementary file 1 — Overview of samples, analyses protocols, and tree Figures S1-S8. (PDF 772 kb) [file 12862_2018_1267_MOESM1_ESM.pdf]
